# Supplementary material for: Automated detection of patients with dementia whose symptoms have been identified in primary care but have no formal diagnosis: a retrospective case–control study using electronic primary care records
Source: BMJ Open. 2021 Jan 22;11(1):e039248. doi: 10.1136/bmjopen-2020-039248 (PMC7831719; doi:10.1136/bmjopen-2020-039248)
Supplement: Supplementary data [file bmjopen-2020-039248supp001.pdf]

ANNEX 1 - DEMENTIA DIAGNOSTIC READ CODES

Read Description	Read Code	Medical Code
**Alzheimers codes**		
Dementia in Alzheimer's disease	Eu00	7664
Dementia in Alzheimer's disease with early onset	Eu000	49263
Dementia in Alzheimer's disease with late onset	Eu001	38678
Dementia in Alzheimer's disease, atypical or mixed type	Eu002	30706
Dementia in Alzheimer's disease unspecified	Eu00z	29386
Alzheimer's disease	F110	1917
Alzheimer's disease with early onset	F1100	16797
Alzheimer's disease with late onset	F1101	32057
[X]Presenile dementia,Alzheimer's type	Eu00011	25704
[X]Primary degen dementia, Alzheimer's type, presenile or	Eu00012	60059
[X]Alzheimer's disease type 2	Eu00013	61528
[X]Alzheimer's disease type 1	Eu00111	46762
[X]Senile dementia,Alzheimer's type	Eu00112	11379
[X]Primary degen dementia of Alzheimer's type, senile ons	Eu00113	43346
[X]Alzheimer's dementia unspec	Eu00z11	8195
	15	
**Vascular Dementia Codes**		
Vascular dementia	Eu01	6578
Arteriosclerotic dementia	E004	19477
Multi-infarct dementia	Eu011	8634
Subcortical vascular dementia	Eu012	8934
Mixed cortical and subcortical vascular dementia	Eu013	31016
Other vascular dementia	Eu01y	55313
Vascular dementia unspecified	Eu01z	19393
Uncomplicated arteriosclerotic dementia	Eu0040	43089
Arteriosclerotic dementia with delirium	Eu0041	56912
Arteriosclerotic dementia with paranoia	Eu0042	55467
Arteriosclerotic dementia with depression	Eu0043	43292
Arteriosclerotic dementia NOS	Eu004z	42279
[X]Vascular dementia of acute onset	Eu01000	46488
[X]Predominantly cortical dementia	Eu01111	55838
[X] Multi-infarct dementia	Eu01100	11175
	15	
**Dementia in other diseases**		
[X] Dementia in other diseases classified elsewhere	Eu02y00	64267
Dementia in Pick's disease	Eu020	28402
Circumscribed brain atrophy including Pick's disease	F111	?
Dementia in Creutzfeldt-Jackob disease	Eu021	54106
Dementia in Huntingdon's disease	Eu022	37014
Dementia in Parkinson's disease	Eu023	9509
Dementia in HIV disease	Eu024	4485
Dementia in conditions EC	E041.00	25386
Alcoholic dementia NOS	Eu10711	27342
[X] Alcoholic dementia NOS	Eu10711	26323
Other alcoholic dementia	E012	54505
Drug-induced dementia	E02y100	62132
Lewy body disease	F116	7572
Lewy body dementia	Eu025	26270
	14	
**Dementia not otherwise classified**		
Unspecified dementia	Eu02z	4693
Senile/Presenile dementia	Eu00..12	1350

Presenile dementia	E001	15165
Uncomplicated presenile dementia	E0010	46202
Presenile dementia with delirium	E0011	49513
Presenile dementia with paranoia	E0012	30032
Presenile dementia with depression	E0013	27677
Presenile dementia NOS	E0014	38438
[X] Presenile dementia NOS	Eu02z11	48501
Uncomplicated senile dementia	E000	7323
Senile dementia	E00..11	1916
Senile dementia with depressive or paranoid features	E002	44674
Senile dementia with paranoia	E0020	18386
Senile dementia with depression	E0021	21887
Senile dementia with depressive or paranoid features NOS	E002z	41089
Delirium superimposed on dementia	Eu041	53446
Senile dementia with delirium	E003	37015
Senile degeneration of the brain, not elsewhere classified	F112	29512
	18	
**Total N codes**	**62**	
