# Supplementary material for: Automated detection of patients with dementia whose symptoms have been identified in primary care but have no formal diagnosis: a retrospective case–control study using electronic primary care records
Source: BMJ Open. 2021 Jan 22;11(1):e039248. doi: 10.1136/bmjopen-2020-039248 (PMC7831719; doi:10.1136/bmjopen-2020-039248)
Supplement: Supplementary data [file bmjopen-2020-039248supp002.pdf]

Cognitive Decline

Read term	Read code	Medcode
Cognitive decline	28E..00	7674
GDS level 2 - very mild cognitive decline	3AE1.00	65856
GDS level 3 - mild cognitive decline	3AE2.00	60263
GDS level 4 - moderate cognitive decline	3AE3.00	60726
GDS level 5 - moderately severe cognitive decline	3AE4.00	70057
GDS level 6 - severe cognitive decline	3AE5.00	94717
GDS level 7 - very severe cognitive decline	3AE6.00	72520
[X]Mild cognitive disorder	Eu05700	11936
[X]Oth & unspec symptom/sign involv cognit funct/awareness	Ryu5100	52939
Health of the Nation Outcome Scale item 4 - cognitive probl	ZRLfE00	35194
Impaired cognition	Z7C1.00	10822
Cognitive function observations	Z7C..00	18906

Cognitive Screening Tests

Read term	Read code	Medcode
Cognitive assessment	311B.00	95853
GPCOG - general practitioner assessment of cognition	38Dv.00	101074
GPCOG (GP assessment of cognition) patient examination	38Dv000	103076
GPCOG (GP assessment of cognition) informant interview	38Dv100	101710
Six item cognitive impairment test	3AD3.00	35305
Cognitions questionnaire	ZR3a.00	36650
Cognitive failures questionnaire	ZR3b.00	66080
Microcog - assessment of cognitive function	ZRa2.00	22257
Neurobehavioural cognitive status examination	ZRaY.00	42059
Rancho scale - levels of cognitive functioning	ZRd..00	92756
Recognition memory test	ZRh6.00	96881

RMT - Recognition memory test	ZRh6.11	93236
Smith cognitive questionnaire	ZRkL.00	96828
Health of the Nation Outcome Scale item 4 - cognitive probl	ZRLfE00	35194
Kendrick cognitive tests for the elderly	ZRV9.11	63604
Lowenstein OT cognitive assessment	ZRVa.00	26264
LOTCA - Lowenstein OT cognitive assessment	ZRVa.11	98256
Measurement of cognitive linguistic ability	ZRVt.00	62114
MCLA - Measurement of cognitive linguistic ability	ZRVt.11	93194

Dementia Annual Review

**Read term**	**Read code**	**Medcode**
Dementia annual review	6AB..00	12710

Memory Loss Codes

**Read term**	**Read code**	**Medcode**
Memory loss - amnesia	1B1A.00	1993
Poor auditory sequential memory	1B1a.00	40821
Amnesia symptom	1B1A.11	3639
Memory loss symptom	1B1A.12	5777
Memory disturbance	1B1A.13	2908
Temporary loss of memory	1B1A000	27788
Short-term memory loss	1B1A100	103453
Transient global amnesia	1B1S.00	28278
Poor visual sequential memory	1B1Y.00	39507
Disturbance of memory for order of events	1S21.00	67163
Forgetful	28G..00	7742
Memory: own age not known	3A10.00	52947
Memory: present time not known	3A20.00	53146
Memory: present place not knwn	3A30.00	53014
Memory: present year not known	3A40.00	52948
Memory: own DOB not known	3A50.00	53125
Memory: present month not knwn	3A60.00	52825
Memory: important event not kn	3A70.00	52800

Memory: import.person not knwn	3A80.00	52801
Memory: count down unsuccess.	3A91.00	52805
Memory: address recall unsucc.	3AA1.00	53016
Patient forgets to take medication	8BIk.00	68703
Non-alcoholic amnestic syndrome	E040.00	27414
Hysterical amnesia	E201700	4269
Mild memory disturbance	E2A1000	6387
Organic memory impairment	E2A1100	6061
[X]Organic amnesic synd not induced alc/oth psychoact subs	Eu03.00	37072
[X]Dissociative amnesia	Eu44000	40994
Transient global amnesia	G655.00	6489
[D]Amnesia (retrograde)	R00z000	4284
[D]Memory deficit	R00z011	7711
[D]Anterograde amnesia	R00z500	54882
[X]Other amnesia	Ryu5000	70677
Memory disturbance (& amnesia (& symptom))	Z7CE400	51379
Memory loss symptom	Z7CE412	67838
Memory loss - amnesia	Z7CE413	103375
Memory disturbance	Z7CE414	105538
Loss of memory	Z7CE415	102880
Forgetful	Z7CE500	24952
Amnesia	Z7CE600	28406
Memory loss	Z7CE611	10123
Memory gone	Z7CE612	68230
Dysmnesia	Z7CE613	66001
Memory loss - amnesia	Z7CE614	12805
Loss of memory	Z7CE615	19297
LOM - Loss of memory	Z7CE616	12277
Transient global amnesia	Z7CE700	18996
TGA - Transient global amnesia	Z7CE711	19004
Anterograde amnesia	Z7CE800	36767
Antegrade amnesia	Z7CE811	43905
Retrograde amnesia	Z7CE900	35538
RA - Retrograde amnesia	Z7CE911	70703
Impairment of registration	Z7CEA00	49897
Impairment of working memory	Z7CEA11	32367
Impairment of immediate recall	Z7CEA12	59844
Impairment of primary memory	Z7CEA13	65696
Amnesia for remote events	Z7CEB00	66144

Poor memory for remote events	Z7CEB12	37191
Amnesia for recent events	Z7CEC00	61816
Loss of memory for recent events	Z7CEC11	9786
No memory for recent events	Z7CEC12	67802
Amnesia for day to day facts	Z7CED00	68392
Amnesia for important personal information	Z7CEE00	57812
Temporary loss of memory	Z7CEF00	67998
Transient memory loss	Z7CEG00	47882
Memory impairment	Z7CEH00	10514
Memory dysfunction	Z7CEH11	39915
Memory deficit	Z7CEH12	50418
Bad memory	Z7CEH13	26434
Memory problem	Z7CEH14	12057
Poor memory	Z7CEH15	12583
Mixes past with present	Z7CEI00	103922
Memory lapses	Z7CEJ00	19073
Minor memory lapses	Z7CEK00	53507
Mild memory disturbance	Z7CEL00	51724
Distortion of memory	Z7CEM00	51739
Invents experiences to compensate for loss of memory	Z7CEN11	64892
Poor short-term memory	Z7CF800	11410
Short-term memory loss	Z7CF811	10571
Unable to recall random address at five minutes	Z7CFA00	106055
Unable to recall five digit number at five minutes	Z7CFC00	104514
Unable to remember name of current prime minister	Z7CFe00	93856
Forgets what was going to do	Z7CFF00	59515
Forgets what was going to say	Z7CFG00	60600
Cannot remember names of intimates	Z7CFg00	105793
Forgets recent activities	Z7CFH00	41366
Cannot remember birth dates of children	Z7CFh00	54430
Forgets what has just done	Z7CFI00	40091
Cannot remember wedding anniversary	Z7CFi00	62749
Forgets what has just said	Z7CFJ00	93319
Forgets what has just read	Z7CFK00	70895
Forgets what has just seen	Z7CFL00	103479
Forgets what has just heard	Z7CFM00	47994
Poor long-term memory	Z7CFO00	53978
Long-term memory loss	Z7CFO11	47581

Unable to remember motor skills	Z7CFq00	46314
Unable to remember own date of birth	Z7CFQ00	63942
Unable to remember own age	Z7CFS00	32599
Unable to remember new motor skills	Z7CFs00	57750
Cannot remember own age	Z7CFS11	69118
Unable to remember day of the week	Z7CFU00	63849
Memory aided by use of diary	Z7CFw00	59830
Unable to remember today's date	Z7CFW00	66684
Memory aided by use of labels	Z7CFx00	98287
Memory aided by use of lists	Z7CFz00	67951
Delayed verbal memory	Z7CGP00	98798

MMSE

**Read term**	**Read code**	**Medcode**
Mini-mental state examination	388m.00	82481
MMSE score	388m.11	83484
Mini mental state score	388V.00	11862
Mini-mental state examination	ZRaA.00	10503
MMSE - Mini-mental state examination	ZRaA.11	10493
Modified mini-mental state 3MS examination	ZRaA100	59387
Modified mini-mental state examination	ZRaA200	36637

Referral to memory assessment services

**Read term**	**Read code**	**Medcode**
Referral to memory clinic	8HTY.00	82481
Seen in memory clinic	9Nk1.00	83484

Referral to Psychiatrist, Neurologist or Geriatrician

**Read term**	**Read code**	**Medcode**
Neurological referral	8H46.00	2964
Geriatric referral	8H47.00	1511
Referral to community geriatrician	8H47000	105235
Psychiatric referral	8H49.00	2189
Referral to psychogeriatrician	8H4D.00	5338
Referral to neurologist	8H4h.00	95859

Referral to neurology special interest general practitioner	8H4Y.00	55362
Psychiatric self-referral	8HJ3.00	27640
Geriatric self-referral	8HJ4.00	68924
Neurology self-referral	8HJE.00	40890
Urgent referral to psychiatrist	8HlB.00	95936
Private referral to neurologist	8HVL.00	13653
Private referral to geriatrician	8HVM.00	29200
Private referral to psychiatrist	8HVO.00	13677
Private referral to psychogeriatrician	8HVS.00	34532
Referral to care of the elderly physician	ZL5A200	12077
Referral to geriatrician	ZL5A211	11634
Referral to neurologist	ZL5AO00	10448
Referral to psychiatrist	ZL5B.00	10002

Alzheimer's prescription Product Codes (specific to CPRD)

Code	Product Description	Molecule	Dose, format and route
7329	Galantamine 20mg/5ml oral solution sugar free	Galantamine hydrobromide	4mg/1ml, Oral solution, Oral
36976	Rivastigmine 4.6mg/24hours transdermal patches	Rivastigmine	4.6mg/24 Hours, Transdermal Patch, Transdermal
33007	RIVASTIGMINE	Unknown	Unknown
56771	Rivastigmine 3mg capsules (Dr Reddy's Laboratories (UK) Ltd)	Rivastigmine hydrogen tartrate	3mg, Capsule, Oral
11751	Rivastigmine 3mg capsules	Rivastigmine hydrogen tartrate	3mg, Capsule, Oral
53922	Donepezil 10mg orodispersible tablets (Consilient Health Ltd)	Donepezil hydrochloride	10mg, Orodispersible tablet, Oral
10255	Galantamine 8mg modified-release capsules	Galantamine Hydrobromide	8mg, Modified Release Capsules, Oral
38976	Memantine 5mg+10mg+15mg+20mg Tablet	Memantine Hydrochloride	5mg+10mg+15mg+20mg, Tablet, Oral
33008	RIVASTIGMINE	Unknown	Unknown
33009	RIVASTIGMINE	Unknown	Unknown
60723	Rivastigmine 6mg capsules (Waymade Healthcare Plc)	Rivastigmine hydrogen tartrate	6mg, Capsule, Oral
56600	Donepezil 5mg tablets (Zentiva)	Donepezil hydrochloride	5mg, Tablet, Oral
11752	Rivastigmine 4.5mg capsules	Rivastigmine hydrogen tartrate	4.5mg, Capsule, Oral
11635	Galantamine 12mg tablets	Galantamine hydrobromide	12mg, Tablet, Oral
48443	Donepezil 10mg orodispersible tablets	Donepezil hydrochloride	10mg, Orodispersible tablet, Oral

53882	Rivastigmine 2mg/ml oral solution	Rivastigmine hydrogen tartrate	2mg/1ml, Oral solution, Oral
2931	Donepezil 10mg tablets	Donepezil hydrochloride	10mg, Tablet, Oral
6225	Memantine 10mg tablets	Memantine hydrochloride	10mg, Tablet, Oral
63217	Donepezil 5mg tablets (A A H Pharmaceuticals Ltd)	Donepezil hydrochloride	5mg, Tablet, Oral
11654	Galantamine 8mg tablets	Galantamine hydrobromide	8mg, Tablet, Oral
56631	Rivastigmine 13.3mg/24hours transdermal patches	Rivastigmine	13.3mg/24hour, Transdermal patch, Transdermal
39240	Memantine 20mg tablets	Memantine hydrochloride	20mg, Tablet, Oral
63951	Rivastigmine 9.5mg/24hours transdermal patches (Actavis UK Ltd)	Rivastigmine	9.5mg/24hour, Transdermal patch, Transdermal
11827	Rivastigmine 2mg/ml oral solution sugar free	Rivastigmine hydrogen tartrate	2mg/1ml, Oral solution, Oral
35088	Donepezil 10mg orodispersible tablets sugar free	Donepezil Hydrochloride	10mg, Orodispersible Tablet, Oral
60107	Donepezil 5mg tablets (Alliance Healthcare (Distribution) Ltd)	Donepezil Hydrochloride	5mg, tablet, Oral
10187	Galantamine 4mg tablets	Galantamine hydrobromide	4mg, Tablet, Oral
58969	Rivastigmine 4.6mg/24hours transdermal patches (A A H Pharmaceuticals Ltd)	Rivastigmine	4.6mg/24hour, Transdermal patch, Transdermal
59871	Donepezil 10mg/5ml oral suspension	Donepezil hydrochloride	2mg/1ml, Oral suspension, Oral
2930	Donepezil 5mg tablets	Donepezil hydrochloride	5mg, Tablet, Oral
35179	Donepezil 5mg orodispersible tablets sugar free	Donepezil hydrochloride	5mg, Orodispersible tablet, Oral
7361	Galantamine 24mg modified-release capsules	Galantamine hydrobromide	24mg, Modified-release capsule, Oral
4597	Rivastigmine 1.5mg capsules	Rivastigmine hydrogen tartrate	1.5mg, Capsule, Oral
61676	Donepezil 1mg/ml oral solution sugar free	Donepezil hydrochloride	1mg/1ml, Oral solution, Oral
58947	Donepezil 10mg tablets (Accord Healthcare Ltd)	Donepezil hydrochloride	10mg, Tablet, Oral
14309	Galantamine 16mg modified-release capsules	Galantamine Hydrobromide	16mg, Modified Release Capsules, Oral
11837	Memantine 10mg/ml oral solution sugar free	Memantine hydrochloride	10mg/1ml, Oral drops, Oral
58709	Donepezil 10mg tablets (A A H Pharmaceuticals Ltd)	Donepezil hydrochloride	10mg, Tablet, Oral

9786	Rivastigmine 6mg capsules	Rivastigmine hydrogen tartrate	6mg, Capsule, Oral
37132	Rivastigmine 9.5mg/24hours transdermal patches	Rivastigmine	9.5mg/24hour, Transdermal patch, Transdermal
48442	Donepezil 5mg orodispersible tablets	Donepezil hydrochloride	5mg, Orodispersible tablet, Oral -
