# Supplementary material for: Automated detection of patients with dementia whose symptoms have been identified in primary care but have no formal diagnosis: a retrospective case–control study using electronic primary care records
Source: BMJ Open. 2021 Jan 22;11(1):e039248. doi: 10.1136/bmjopen-2020-039248 (PMC7831719; doi:10.1136/bmjopen-2020-039248)
Supplement: Supplementary data [file bmjopen-2020-039248supp003.pdf]

Keyword	Stem	misspellings	
**Memory**	memory		
	memori*	memroy	
		memeory	
		memmory	
		meory	
		memort	
		memrory	
		memorry	
**cognition**	cognition	cogition	
	cognitive	cogntion	
	cognit*	cngition	
		congtion	
		cogniton	
		congit*	
		cgnit*	
**forgetfulness**	forgetting	foget*	
	forgetful*	forgot*	
	forget*	forgte	
		fforget*	
		froget*	
**confusion**	confusion	confudes	
	confused	condusef	
	confus*	cofnused	
**dementia**	dementia		
	dement*		
	Alzheimer's	alzheimers	
		alzhemer	
		alzhiemer	
		alzhiemers	
		alzhimer	
		alzhimers	
		alzimer	
		alz*	
**behaviour change**	behaviour	behavour	
	behavior	bahaviour	
	behav*	bahavior	
		bahavioe	
		behavoir	
		behaveour	
**family concerned**	family + concerned	**for family substitute:**	**for concerned substitute**
		relative	conserned
		wife	concern*
		husband	worried
		son	
		daughter	
		partner	
		spouse	
		niece	

		nephew
		sister
		brother
		relative
		neighbour
**3rd Party**	third party consultation	third party consulation
		3rd party consultation
		3rd party consulation
**MMSE**	MMSE	
	mini mental state	
	mini-mental state	
	GP Cog	
