# Supplementary material for: Automated detection of patients with dementia whose symptoms have been identified in primary care but have no formal diagnosis: a retrospective case–control study using electronic primary care records
Source: BMJ Open. 2021 Jan 22;11(1):e039248. doi: 10.1136/bmjopen-2020-039248 (PMC7831719; doi:10.1136/bmjopen-2020-039248)

Appendix 4: Sensitivity analysis.

We removed controls with Dementia annual review codes (N = 146), and Alzheimer's medical prescription codes (154 additional patients).

We re-ran the models as before with the predictors of Alzheimer's medical prescription and dementia annual review removed. We show below that the model performance (based on AUC) dropped in the codes-only models, but was largely maintained in the codes and keywords models.

Table 4.1: Model performance with Alzheimer's medical prescription and Dementia annual review removed from control group and model

	Codes only				Codes and Keywords			
Classifier	AUC	Sensitivity	Specificity	PPV at 7.1% prevalence	AUC	Sensitivity	Specificity	PPV at 7.1% prevalence
Random Forest	0.83	0.69	0.95	0.61	0.93	0.85	0.93	0.49
Logistic Regression	0.83	0.69	0.95	0.61	0.93	0.84	0.92	0.48
Naive Bayes	0.83	0.69	0.95	0.60	0.89	0.79	0.90	0.41

Table 4.2: Feature weights with Alzheimer's medical prescription and Dementia annual review removed from control group and model

	Coded data only			Codes and Keywords		
	Logistic Regression	Random Forest	Naive Bayes	Logistic Regression	Random Forest	Naive Bayes
Cognitive Decline	0.320	0.091	0.529	0.244	0.071	0.529
Cognitive Screening Test	0.008	0.007	0.571	0.083	0.007	0.571
Memory Loss Codes	1	0.799	1	1	0.888	1
MMSE	0.380	1	0.321	0.286	0.994	0.321
Referral To Memory Assessment	0.263	0.269	0.420	0.147	0.132	0.42
Referral To Psych/Geri/Neuro	0.246	0.0776	0.578	0.104	0.022	0.578
Keyword Dementia	-	-	-	0.311	0.923	0.258
Keyword Memory	-	-	-	0.171	1	0.232
Keyword Confusion	-	-	-	0.161	0.34	0.202
Keyword Behaviour	-	-	-	0.028	0.085	0.281
Keyword Cognition	-	-	-	0.025	0.071	0.279
Keyword Family	-	-	-	0.093	0.066	0.064
Keyword MMSE	-	-	-	0.025	0.108	0.280
Keyword Forget	-	-	-	0.033	0.089	0.242
Keyword Third party consultation	-	-	-	0.714	0.251	0.292

Figure 4.1: ROC curves for model with Alzheimer's medical prescription and Dementia annual review removed, codes only.

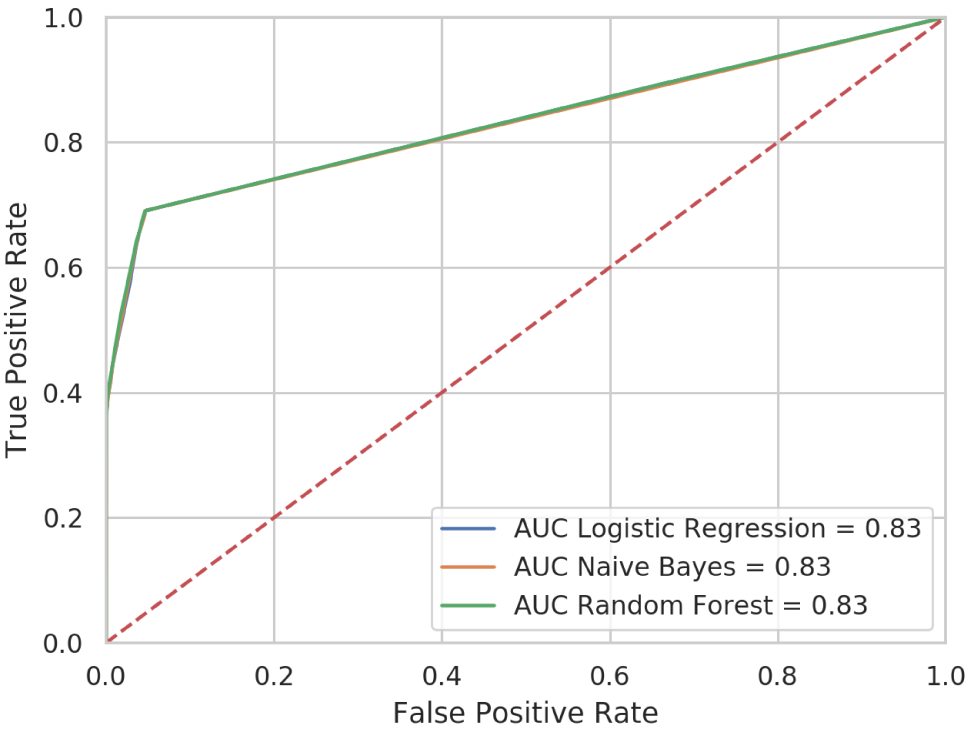

Figure 4.2: ROC curves for model with Alzheimer's medical prescription and Dementia annual review removed, codes and keywords.

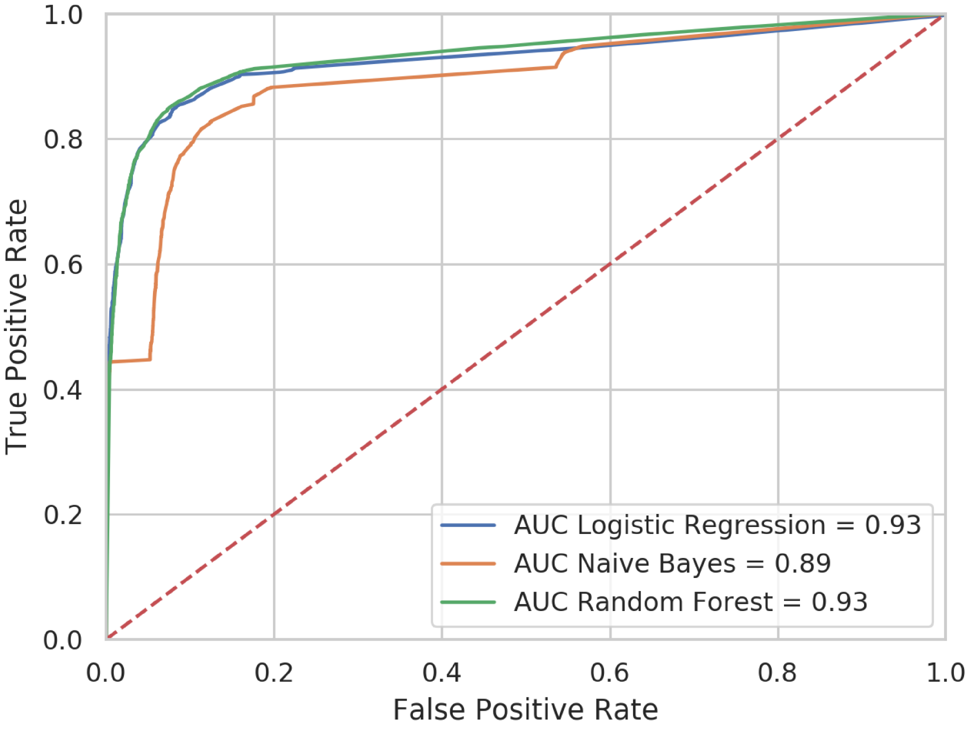

Supplement: Supplementary data [file bmjopen-2020-039248supp004.pdf]
