# Supplementary material for: Automated detection of patients with dementia whose symptoms have been identified in primary care but have no formal diagnosis: a retrospective case–control study using electronic primary care records
Source: BMJ Open. 2021 Jan 22;11(1):e039248. doi: 10.1136/bmjopen-2020-039248 (PMC7831719; doi:10.1136/bmjopen-2020-039248)
Supplement: Author's manuscript [file bmjopen-2020-039248.draft_revisions.pdf]

BMJ Open is committed to open peer review. As part of this commitment we make the peer review history of every article we publish publicly available.

When an article is published we post the peer reviewers' comments and the authors' responses online. We also post the versions of the paper that were used during peer review. These are the versions that the peer review comments apply to.

The versions of the paper that follow are the versions that were submitted during the peer review process. They are not the versions of record or the final published versions. They should not be cited or distributed as the published version of this manuscript.

BMJ Open is an open access journal and the full, final, typeset and author-corrected version of record of the manuscript is available on our site with no access controls, subscription charges or pay-per-view fees (http://bmjopen.bmj.com).

If you have any questions on BMJ Open's open peer review process please email info.bmjopen@bmj.com

BMJ Open

Automated detection of patients with dementia whose symptoms have been identified in primary care but have no formal diagnosis: a retrospective case-control study using electronic primary care records

Journal:	*BMJ Open*
Manuscript ID	bmjopen-2020-039248
Article Type:	Original research
Date Submitted by the Author:	08-Apr-2020
Complete List of Authors:	Ford, Elizabeth; Brighton & Sussex Medical School,<br>Sheppard, Joanne; University of Sussex School of Mathematical and Physical Sciences, Department of Physics and Astronomy; UCL, Medical Physics and Biomedical Engineering<br>Oliver, Seb; University of Sussex School of Mathematical and Physical Sciences, Department of Physics and Astronomy<br>Rooney, Philip; University of Sussex School of Mathematical and Physical Sciences, Department of Physics and Astronomy<br>Banerjee, Sube; University of Plymouth, Faculty of Health<br>Cassell, Jackie; Brighton and Sussex Medical School, Department of Primary Care and Public Health
Keywords:	Dementia < NEUROLOGY, Health informatics < BIOTECHNOLOGY & BIOINFORMATICS, GERIATRIC MEDICINE, PRIMARY CARE, Delirium & cognitive disorders < PSYCHIATRY, Old age psychiatry < PSYCHIATRY

SCHOLARONE™
Manuscripts

I, the Submitting Author has the right to grant and does grant on behalf of all authors of the Work (as defined in the below author licence), an exclusive licence and/or a non-exclusive licence for contributions from authors who are: i) UK Crown employees; ii) where BMJ has agreed a CC-BY licence shall apply, and/or iii) in accordance with the terms applicable for US Federal Government officers or employees acting as part of their official duties; on a worldwide, perpetual, irrevocable, royalty-free basis to BMJ Publishing Group Ltd ("BMJ") its licensees and where the relevant Journal is co-owned by BMJ to the co-owners of the Journal, to publish the Work in this journal and any other BMJ products and to exploit all rights, as set out in our licence.

The Submitting Author accepts and understands that any supply made under these terms is made by BMJ to the Submitting Author unless you are acting as an employee on behalf of your employer or a postgraduate student of an affiliated institution which is paying any applicable article publishing charge ("APC") for Open Access articles. Where the Submitting Author wishes to make the Work available on an Open Access basis (and intends to pay the relevant APC), the terms of reuse of such Open Access shall be governed by a Creative Commons licence – details of these licences and which Creative Commons licence will apply to this Work are set out in our licence referred to above.

Other than as permitted in any relevant BMJ Author's Self Archiving Policies, I confirm this Work has not been accepted for publication elsewhere, is not being considered for publication elsewhere and does not duplicate material already published. I confirm all authors consent to publication of this Work and authorise the granting of this licence.

For peer review only

Automated detection of patients with dementia whose symptoms have been identified in primary care but have no formal diagnosis: a retrospective case-control study using electronic primary care records

Elizabeth Ford[1]
Joanne Sheppard [2,3]
Seb Oliver[2]
Philip Rooney[2]
Sube Banerjee[4]
Jackie Cassell[1]

1) Department of Primary Care and Public Health, Brighton and Sussex Medical School
2) Department of Physics and Astronomy, University of Sussex.
3) Medical Physics and Biomedical Engineering, University College London
4) Faculty of Health, University of Plymouth

Corresponding Author:
Elizabeth Ford
Department of Primary Care and Public Health, Brighton and Sussex Medical School
Watson Building, Village Way, Falmer
Brighton BN1 9PH
Tel: 01273 641974 email: e.m.ford@bsms.ac.uk

Keywords
Dementia; early diagnosis; primary care; electronic health records; machine learning; free text.

For peer review only

Abstract

Objectives: United Kingdom (UK) statistics suggest only two-thirds of patients with dementia get a diagnosis recorded in primary care. General practitioners (GPs) report barriers to formally diagnosing dementia, so some patients may be known by the GP to have dementia but missing a diagnosis in their patient record. We aimed to produce a method to identify these "known but unlabelled" patients with dementia using data from primary care patient records.

Design: Retrospective case-control study using routinely collected primary care patient records from CPRD.

Setting: UK general practice.

Participants: English patients aged >65y, with a coded diagnosis of dementia recorded 2000-2012 (cases); matched 1:1 to patients with no diagnosis code for dementia (controls).

Interventions: 8 coded and 9 keyword concepts indicating symptoms, screening tests, referrals and care for dementia recorded in the 5 years before diagnosis. We trialled machine learning classifiers to discriminate between cases and controls (logistic regression, naïve Bayes, random forest).

Primary and Secondary Outcomes: Outcome variable was dementia diagnosis code; accuracy of classifiers was assessed using Area Under the Receiver Operating Characteristic (AUC); order of features contributing to discrimination was examined.

Results: 93,426 patients were included; median age 83 years (64.8% female). Three classifiers achieved high discrimination and performed very similarly. AUCs were 0.87-0.90 with coded variables rising to 0.90-0.94 with keywords added. Feature prioritisation was different for each classifier, commonly prioritised features were Alzheimer's prescription, dementia annual review, memory loss, and dementia keywords.

Conclusions: It is possible to detect patients with dementia who are known to GPs but unlabelled with a diagnostic code, with a high degree of accuracy in electronic primary care record data. Using keywords from clinic notes and letters improves accuracy compared to coded data alone. This approach could improve identification of dementia cases for record-keeping, service planning, and delivery of good quality care.

For peer review only

For peer review only - http://bmjopen.bmj.com/site/about/guidelines.xhtml

Strengths and Limitations of this Study:

- Our study used all patients in the CPRD GOLD database who were over 65 years old and had a first recorded dementia diagnosis in 2000-2012 (N= 46,713). These were matched by age, sex and general practice to controls who had no diagnosis of dementia in their entire patient record.
- We trialled a number of binary classifiers which all performed similarly, but within which different features were prioritised as most important for classification; combining machine learning approaches might have increased the accuracy.
- The addition of keywords from clinic notes and text to coded information improved the accuracy of the classifiers but free text notes are routinely stripped out of research datasets and are hard to access.
- A limitation is that our model was trained on cases who all received a dementia diagnosis code at the end of the study period, who may differ systematically from those who never received a code.
- This model should be replicated in a cohort study using similar primary care patient data and then trialled in a primary care network, to improve the completeness of dementia registers and to aid service planning and care.

For peer review only

Introduction

Dementia is a term used to describe a group of conditions characterised by a progressive decline in cognitive function and consequent functional limitation.[1] In the United Kingdom (UK), 1.3% of people live with dementia, the majority of whom are over 65 years. Prevalence in all over 65s is 7.1%, rising from 1.8% in the 65-69 age group to over 33% in the over 90s.[2] The challenges of dementia affect individuals, their family carers, health and care services and systems and society as a whole, and addressing them has become a national and international priority due to population ageing. It is expected that the cost of the disease to society will double between 2014 and 2040, reaching £55bn per year in the UK.[2]

The UK government has recognised the benefits of an early diagnosis of dementia and has made improving rates of diagnosis a key focus of dementia policy.[3] Whilst the disease is incurable, a diagnosis gives the patient a better chance of achieving a good quality of life while living with dementia.[4] Individuals can plan and make their wishes known while they still have capacity. Services provided by health and social care professionals may help people with dementia to adapt their home and identify their care needs, and thus avoiding or delaying having to move into institutionalised care. Furthermore, diagnosis allows people with Alzheimer's disease to be prescribed medication and non-pharmaceutical treatments to maximise their cognitive function and quality of life. These treatments may have a particular benefit in the early stages of the illness.[5, 6]

In recognition of this, general practitioners (GPs) in the UK are incentivised to initiate the diagnosis of dementia and to keep a register of patients diagnosed with dementia so that they can provide care proactively.[7] They are then reimbursed for ensuring a face-to-face review for each patient with dementia every 12 months. Data collected in GP patient records about dementia cases (using a prescribed set of Dementia Read codes) is used by local Clinical Commissioning Groups (CCGs) as well as national bodies such as NHS England and Public Health England to monitor local and national diagnosis rates and services.[8]

Based on an epidemiological study conducted by the Medical Research Council in 1998 a true prevalence of 6.6% was estimated in people over the age of 65.[9] However, based on dementia registers in the UK, the observed prevalence was found to be 3%.[9] This gap has closed substantially, due to schemes like the National Dementia Strategy,[10, 11] but coded diagnoses of dementia in GP

For peer review only - http://bmjopen.bmj.com/site/about/guidelines.xhtml

patient records still only identify two-thirds of cases, compared to the expected prevalence.[8, 12] While this is higher than health services in many countries (for example Lang et al.,[13] found an average rate of undetected dementia of 62% across Europe, North America and Asia) a third of people with dementia may not be receiving appropriate care.

Qualitative studies with GPs have indicated that GPs are sometimes reluctant to diagnose dementia,[14-16] and describe dementia as “a complex condition that takes time to diagnose”.[17] GPs have reported that that diagnosing dementia early was not particularly important, and may in fact be harmful to some patients.[17] GPs tread carefully when initiating and communicating a diagnosis of dementia to their patients, as it is a stigmatised condition with what seems a bleak prognosis.[16, 18] In addition, as elderly patients often have multiple conditions, GPs report that it is difficult to integrate multiple clinical guidelines,[19] and that “dementia or memory problems [are] right at the bottom of the list”.[20] These findings suggest that patients with undiagnosed dementia could fall into two groups. First, those whose memory loss or cognitive symptoms have not been identified by the GP, possibly because they do not present to the GP very often, or they have not communicated any memory related symptoms. Second, there is likely to be a group of patients who are known by primary care clinicians to be experiencing memory problems, and may even have been screened for dementia or referred for memory assessment, but who have not, for many possible reasons, received a diagnostic code for dementia in their GP record.[14, 18, 21] For these patients who are “known but unlabelled”, there may be other indicators of dementia recognition and diagnosis in the patient record which could enable them to be detected using an algorithm which combines multiple indicators of the disease.

Reviews of data-driven dementia risk prediction models,[22, 23] show that the majority of work in the past has aimed at producing risk scores which estimate the patient’s risk of developing dementia at some future date. Only a few of these risk scores have been developed on routinely collected primary care data of which, a notable example is Walters et al.[24] Automated early detection of emergent cases has received less attention. Few attempts have been made to produce models which detect dementia which is current but unrecognised by the GP; the only examples using UK primary care data (known to the authors) are currently Jammeh et al.,[25] and Ford et al.[26] Other studies have approached this problem manually, for example Russell et al.,[27] aimed to improve recording in dementia registers by a hand-searching exercise, recognising the need to “‘clean up dementia coding and records”. This successful exercise increased the identification rate for dementia in participating GP practices by 8.8%.

For peer review only - http://bmjopen.bmj.com/site/about/guidelines.xhtml

Following success in detecting unrecognised patients with dementia,[26] in this study we aimed to identify the second group of “known but unlabelled” patients with dementia. A second aim was to evaluate whether the model could be improved by the use of keyword information in clinic notes and letters found in the patient records, rather than relying solely on information captured in clinical codes. Automatically detecting these patients would help with improving the quality of data in dementia registers, enabling ongoing care and support of individual patients with dementia, and giving commissioning bodies and service planners more accurate prevalence estimates.

Methods

Study Design

A retrospective case-control study using routinely collected primary care patient records from England.

Data Source

This study used data from the UK Clinical Practice Research Datalink (CPRD). It was established in 1987, and now contains anonymized healthcare records from more than 7 million current patients and represents up to 13% of the UK population.[28, 29] Patients are broadly representative of the UK general population in terms of age, sex and ethnicity. CPRD includes longitudinal observational data from general practitioner (GP) electronic health record systems in primary care practices, including medical diagnoses, referrals to specialists and to secondary care, testing and interventional procedures conducted in primary care, lifestyle information (e.g. smoking, exercise) and drugs prescribed in primary care.[29] Data are captured using a structured hierarchical vocabulary called Read Codes, these were developed by a UK GP, Dr James Read, in the 1980s.[30] They map to other nomenclatures such as ICD, SNOMED-CT, and International Classification of Primary Care (ICPC) codes. Each Read code represents a term or short phrase describing a health-related concept. There are over 200,000 different codes, which are sorted into categories (diagnoses, processes of care and medication) and subchapters.[31] Each clinical entity is represented by an alphanumeric code and a Read term which is the plain language description.

GPs also record clinical information in free text clinic notes and letters. CPRD held these clinic notes at the time of study data extraction, and made these available via either keyword search or release of full

text for small samples, following 3-stage manual de-identification of text and approval of a protocol. Since 2013 free text notes and letters have not been extracted into CPRD from GP practices, and historic clinic text became unavailable to researchers in 2016. CPRD also offers data linkages with other NHS and administrative datasets but no linked data were used in this study.

Study Population

CPRD provided us with longitudinal data from patients with a dementia code (cases) recorded between 2000-2012, as defined in a code list of dementia diagnostic codes (general dementia, vascular and Alzheimer's dementia codes) that we developed drawing on code lists in Russell et al.,[27] and Rait et al.,[32] and reported in Ford et al.[26] (Appendix 1) Patients were 65 years or older, and were required to have records available for at least three years prior to a first diagnosis code recorded between 2000 and 2012. All patients in the CPRD GOLD database meeting these criteria were selected to maximise sample size and reduce the possibility of selection bias. Control patients were randomly sampled from patients who had no dementia codes anywhere in their record (to reduce the likelihood that they just had a delayed diagnosis), who matched cases on age, sex, and general practice, and who also had a minimum of three years' of records before the diagnosis date of their matched case (known as the "index date"). This resulted in a near-perfect 1-to-1 match between cases and controls, a few cases did not receive a match. This resulted in 47,858 cases and 47,663 controls.

The entire coded patient record was extracted for each patient; in addition, CPRD ran a search through free text notes and letters for keywords for all patients (described below) in the 24 months prior to the index date. For the keyword search, CPRD returned structured data comprised of a count of the keywords in each keyword group, found in each free text entry in the record. The study team did not have access to the free text of any patient for this study.

Refining participants

From the extracted dataset, cases without matched controls, and all patients lacking five years of data prior to index date were removed along with their corresponding match. We removed cases for whom we could not find a dementia read code within the study periods, and controls who did have a dementia code, and removed these along with their matched patients. See Figure 1 for a flowchart of final participant selection.

For peer review only - http://bmjopen.bmj.com/site/about/guidelines.xhtml

- insert Figure 1 here -

Legend: Figure 1: Final Participant Selection.

Feature selection

Code lists:

Using an expert consensus approach, with an epidemiologist, a psychiatrist and a GP, we identified a list of 8 potential clinical concepts which would be indicators of the onset of dementia, initiation of the diagnostic pathway for dementia or evidence of continuing care for dementia, and which would be captured within the Read codes. Our final list of coded concepts were: cognitive decline, memory loss symptoms, mini-mental state examination (MMSE), other cognitive screening test, referral to memory clinic, referral to psychiatrist, neurologist or geriatrician, dementia annual review, and Alzheimer's specific prescription codes. Relevant code lists were sought from published papers, and where these were not available, lists were drawn up by author EF and checked by author PR (Appendix 2).

Keywords from clinic notes and letters:

We consulted with 17 GPs, asking them to suggest keywords representing terms that they might use when considering that a patient might be developing dementia. From these we identified 9 concepts: memory (suggested by 17 GPs); cognition (6 GPs); forgetful (14 GPs) MMSE or other screening test (6 GPs); confused (9 GPs); dementia (3 GPs); behaviour (5 GPs); family concern (6 GPs) and third party consultation (1 GP). We then generated a list of concept variations and spelling variations for each keyword group (Appendix 3).

Analysis

Only data from 5 years before the index date, up to the index date for each patient was used. Each patient's record was searched to find all instances of the Read codes referring to the features described above. If any codes were found for a feature within a patient's records, a dummy variable representing that feature was set to "1" otherwise they were defaulted to "0". Likewise, if evidence of keyword was found, the dummy variable for keywords were set to "1". A dummy variable for "any keyword" was also included. Other studies have also favoured binary features in order to reduce the influence on the data of the number of times a patient visits their GP.[25]

Random forest, logistic regression and naive Bayes algorithms were used as they are effective supervised machine learning methods for the analysis and classification of binary data. These were

For peer review only - http://bmjopen.bmj.com/site/about/guidelines.xhtml

implemented by using sci-kit learn with Python version 3.7.1 with the "LogisticRegression", "BernoulliNB" and "RandomForestClassifer" packages with the seed set to 42 for reproducibility. The dataset was split into a 70% set for training the classifiers and a 30% testing set to assess model accuracy.

To assess the accuracy of the models the Area Under the Receiver Operator Curve (AUC) was calculated, which plots the true positive rate against the false positive rate for every possible cut-off of the predictive model. In addition, sensitivity and specificity values were calculated for model cut-offs where these values were equally weighted. Positive predictive values (PPV) were also calculated, using the estimated true UK prevalence of dementia in people over 65 years of 7.1%, as our sample had an artificial prevalence of 50% dementia.[2]

To assess which features contributed most to the models, we generated relative indices, with the most predictive feature weighting set at 1, and other features shown relative to this. This was instead of reporting the exact outputs from each machine learning algorithm (such as β weights from logistic regression), because we wanted models to be directly compared.

Results

Study Participants

The final dataset consisted of 93,426 patients with a median age of 83 years old (range 65-110). There were 32,876 male participants (35.2%) with a median age of 81 years old (range 65-100), and 60,550 female participants (64.8%) with a median age of 83 years old (range 65-110). 46,713 were cases and 46,713 were controls. The mean number of codes recorded per patient in the 5 years prior to index date was 614 for the controls and 586 for the cases (range 0-7,281).

Numbers of features by case and control group

Cases had much higher rates of all chosen features, as can be seen in Table 1. The most common coded feature in dementia cases was memory loss codes (46%), followed by Dementia annual review codes (37%). Keywords were all common throughout cases, the most frequent were family concern (81%), memory (58%) and dementia (51%). All keywords were more common in cases than controls, except for

For peer review only - http://bmjopen.bmj.com/site/about/guidelines.xhtml

third party consultation, and family concern was also high in controls (51%), although not as high as in the cases.

Table 1: Number and proportion of cases and controls who had each feature.

Feature	N cases with features (%) (N = 46,713)	N controls with feature (%) (N = 46,713)
**Codes**		
Cognitive decline codes	3,276 (7.01)	240 (0.51)
Cognitive screening test	888 (1.90)	124 (0.27)
Dementia annual review	17,372 (37.19)	151 (0.32)
Memory loss codes	21,282 (45.56)	1,387 (2.97)
MMSE test	9,574 (20.32)	600 (1.28)
Referral to memory assessment service	3,464 (7.42)	117 (0.25)
Referral to old age psychiatrist, neurologist or geriatrician	12,683 (27.15)	2,227 (4.77)
Alzheimer's medication prescription	10,044 (21.50)	203 (0.43)
**Keywords**		
Memory	27,025 (57.85)	3,815 (8.17)
Cognition	8,873 (18.99)	2,178 (4.66)
Forgetful	14,272 (30.55)	3,397 (7.27)
MMSE or screening test	11,969 (25.62)	2,189 (4.69)
Confused	20,530 (43.95)	5,334 (11.42)
Dementia	23,935 (51.24)	2,848 (6.10)
Behaviour change/problems	5,388 (11.53)	2,132 (4.56)
Family concerned	37,779 (80.87)	23,718 (50.77)
Third party consultation	706 (1.51)	1,854 (3.97)

Model Accuracy

The eight coded clinical concepts were used to train the three classifiers to predict which patients' record would end with a dementia diagnosis, and which patients' would not. Trained models were then tested on the testing dataset and accuracy of discrimination was assessed using sensitivity and specificity. Using only coded variables, the three models produced a high level of correct discrimination between cases and controls. The three classifiers performed very similarly with AUCs of 0.87-0.90 (Table 2 and Figure 2).

We then added the nine keywords to the code-only models, and found that discrimination improved up to an AUC of 0.94 in the logistic regression and random forest and 0.90 in the Naïve Bayes classifier. It can be seen in Table 2 that the inclusion of the keywords increased the sensitivity of the models by around 8%, without any detrimental effect on specificity.

For peer review only - http://bmjopen.bmj.com/site/about/guidelines.xhtml

Table 2: Discrimination of three models between cases and controls

	Codes only				Codes and Keywords			
Classifier	AUC	Sensitivity	Specificity	PPV at 7.1% prevalence	AUC	Sensitivity	Specificity	PPV at 7.1% prevalence
Random Forest	0.90	0.77	0.92	0.46	0.94	0.85	0.92	0.46
Logistic Regression	0.90	0.76	0.93	0.49	0.94	0.84	0.93	0.51
Naive Bayes	0.87	0.78	0.91	0.45	0.90	0.80	0.91	0.43

- insert Figure 2 here -

Legend: Figure 2: ROC showing discrimination between dementia cases and controls by three machine learning models with codes only.

- insert Figure 3 here -

Legend: Figure 3: ROC showing discrimination between dementia cases and controls by three machine learning models with both codes and keywords.

Feature weighting

The relative weighting of each feature in the different models, with the most predictive feature weighting set at 1, and other features shown relative to this, is shown in Table 3, separated by models for the coded data only and for codes and keywords together. The table shows that the three different models had substantial differences in the weighting of the different features when using only keywords. The logistic regression weighted most highly dementia annual review codes and prescriptions of Alzheimer's medication, while the random forest weighted memory loss codes highest and the naïve Bayes classifier weighted referral for memory assessment as the most informative feature.

Looking at the combined codes and keywords, similar codes were most important in the three models, whereas the most important keyword was the word memory (rated highest feature in the random forest model), followed by keywords for dementia. Interestingly, the logistic regression classifier prioritised third party consultation, the only feature higher in the controls than the cases. The Naïve Bayes classifier prioritised coded variables higher than all keywords.

For peer review only - http://bmjopen.bmj.com/site/about/guidelines.xhtml

Table 3: Relative weighting of each feature in the three classifiers

	Coded data only			Codes and Keywords		
	Logistic Regression	Random Forest	Naive Bayes	Logistic Regression	Random Forest	Naive Bayes
Alzheimer's Medication	0.727	0.354	0.912	0.676	0.339	0.912
Cognitive Decline	0.378	0.066	0.888	0.260	0.061	0.880
Cognitive Screening Test	0.025	0.006	0.997	0.076	0.010	0.997
Dementia Annual Review	1	0.845	0.957	0.917	0.888	0.957
Memory Loss Codes	0.622	1	0.593	0.456	0.964	0.593
MMSE	0.344	0.187	0.729	0.199	0.157	0.729
Referral To Memory Assessment	0.312	0.049	1	0.143	0.033	1
Referral To Psych/Geri/Neuro	0.345	0.292	0.509	0.248	0.210	0.509
Keyword Dementia	-	-	-	0.494	0.975	0.473
Keyword Memory	-	-	-	0.251	1	0.422
Keyword Confusion	-	-	-	0.238	0.444	0.363
Keyword Behaviour	-	-	-	0.037	0.091	0.520
Keyword Cognition	-	-	-	0.031	0.078	0.515
Keyword Family	-	-	-	0.158	0.079	0.113
Keyword MMSE	-	-	-	0.019	0.119	0.515
Keyword Forget	-	-	-	0.051	0.087	0.439
Keyword Third party consultation	-	-	-	1	0.200	0.544

Discussion

We produced a set of binary coded variables that could be combined in a statistical model and which could accurately classify patients who went on to have a dementia diagnosis formally coded. This model could be take forward to the clinic to identify "known but unlabelled" patients with dementia in primary care, thus improving record keeping, prevalence estimates, and ongoing care for these patients. We also investigated whether adding in information from the clinic notes and letters to the model, in the form of a simple keyword extraction, could add further value to the approach. The addition of free text keywords improved the discrimination of the model to an excellent AUC of 0.94, suggesting this model would perform well in the clinic. The improved performance with the addition of free text keywords was achieved because of increased sensitivity of the model, with no loss of specificity, as has been shown in a range of other case-identification studies incorporating free text.[33] This study provides evidence that hidden data in these free text fields can substantially improve automated detection of cases, adding weight to arguments that free text data are valuable for research, if acceptable privacy preserving methods for data-sharing (such as automated information extraction behind a clinical firewall) can be agreed upon.

For peer review only - http://bmjopen.bmj.com/site/about/guidelines.xhtml

We tried three different machine learning approaches which are suitable for binary data and found all performed similarly, although they weighted the various features differently in their classifications. This is likely to be due to the different construction of the algorithms within each method, for example, random forest algorithms are thought to weight most highly the commonest features in the dataset, rather than features which are rarer but differ more substantially between the two groups. Future work could look at whether there is added value in combining the three approaches, as ensemble methods can sometimes outperform any of the single approaches alone, especially where the different methods are clearly prioritising different features to achieve their performance, although most have been trialled on multi-class rather than binary classification.[34]

Our study is a novel contribution to the literature. Studies have previously aimed to detect dementia that was unknown to primary care practitioners, by manual [35-37] and automated methods,[25] using data from primary care records, however, none have made the distinction between patients with dementia unknown to the GP and those known to the GP but nevertheless unrecorded with a formal diagnostic code. It is not clear how many of the “undiagnosed” cohort may be “known but unlabelled”, and it would be worth investigating this further to estimate the proportions of each group. Our team has also used social science methods, interviewing and surveying GPs and patients to understand what is happening in this apparent “diagnosis gap” for dementia in UK primary care. We suggest that this dual “data science and social science” approach to understanding and utilising data in patient records for clinical decision or diagnostic support is valuable, as it exposes the underlying human decision-making and behaviour which influences how the data are created. These insights can be drawn on to improve clinical prediction models and make them more relevant for key stakeholders, which is likely to improve implementation.

Strength and limitations

This study used a large sample of English patients who were all over the age of 65, and patients registered in CPRD are thought to be representative of patients in England as a whole. However, to ensure that this model is robust to different patient groups, it should now be evaluated in a new patient sample, perhaps in one which is produced from a different general practice computer system (CPRD Gold data is harvested from VISION software). A further strength is that we specified a very simple

For peer review only - http://bmjopen.bmj.com/site/about/guidelines.xhtml

model which dichotomised the presence of an indicator from a code list, this means the model can be readily operationalised in any GP record system and should be robust to number and order of code accrual in the patient record.

However, there are important limitations in our study design which could be addressed in future work. We used a matched case-control design, in which we had an artificial prevalence of 50% and in which the contribution of age and sex to the model was controlled for in the matching. The accuracy of the model should be assessed in a cohort study where the prevalence is lower and also where age can be added as a predictor. A second limitation is that our model was trained on cases who all received a dementia diagnosis code at the end of the study period. There are likely to be a number of dementia cases who never receive a dementia code, and it remains a possibility that these patients differ in systematic ways from cases who do go on to receive a code. The ability of our model to detect patients who never receive a code is therefore unknown. An evaluation in a GP practice, where the predictions of the model are assessed and validated by the practising GP, would give us further information about its performance in real-life clinic settings. Thirdly, we used a very simple keyword extraction to access the keyword data, and did not incorporate any natural language processing to identify negation, hedging, subject or timing of the words we identified. We may therefore have over-sampled instances of the clinical concepts being recorded about the patients, however this would have affected both cases and controls equally and is most likely to have resulted in a bias towards the null. It was important for our study design that we chose simple feature extraction methods which could potentially be recreated in a busy clinic.

Implications and future steps

Our model shows good accuracy in identifying dementia patients who have been detected as likely to have dementia but who currently have no diagnostic code for the condition. As outlined above, two further validation studies are now indicated; first, a replication in a cohort study using similar primary care patient data and second, a proof of concept trial in a local GP practice or primary care network. We hope if these two further pieces of work indicate the same excellent discrimination between patients with dementia and those without, and following steps to determine the threshold resulting in high specificity and sensitivity, this model could be adopted by GPs and CCGs to improve the quality of their dementia registers and to aid service planning.

For peer review only - http://bmjopen.bmj.com/site/about/guidelines.xhtml

Conclusions

We have shown that a simple model made up of indications of dementia symptoms, screening tests, referral and follow up care can identify primary care patients who have recognised dementia but no diagnostic code for it in their record. This model can be taken forward for further validation, and used by GPs and commissioners to improve the quality of data on patients with dementia and as a result, improve dementia care and services.

Supplementary Materials

Appendix 1: Dementia Code List

Appendix 2: Features Code Lists

Appendix 3: List of Keywords

Appendix 4: RECORD Checklist

Acknowledgements

This work uses data provided by patients and collected by the NHS as part of their care and support. #datasaveslives.

Funder

This project was funded by a grant from the Wellcome Trust ref. 202133/Z/16/Z. The funder had no role in study design, data collection and analysis, decision to publish, or preparation of the manuscript.

Ethics approval and consent to participate

This study was approved by the Independent Scientific Advisory Committee at the Medicines and Healthcare Products Regulatory Authority, UK, protocol number 15_111_RA. Following approval, administrative permissions to access and use the electronic patient records were granted by Clinical Practice Research Datalink (CPRD.com).

Consent for publication

For EHR data: Not applicable

Patient and Public Involvement

Prior to applying for funding a focus group was conducted with people with dementia and their carers to guide the development of the research question. Patients were not involved in the design of the study or the acquisition of data. Results have been disseminated through a series of public presentations to patient interest groups, through a study website (www.bsms.ac.uk/astrodem) and through social media.

For peer review only - http://bmjopen.bmj.com/site/about/guidelines.xhtml

Availability of data and materials'

The data that support the findings of this study are available from Clinical Practice Research Datalink (CPRD; www.cprd.com) but restrictions apply to the availability of these data, which were used under license for the current study, and so are not publicly available. For re-using these data, an application must be made directly to CPRD.

Authors' contributions

EF, SO and JC conceived and directed the study. EF and RH drew up code lists and PR checked these. PR managed the data. JS conducted the analyses. PR and SO gave data analysis advice. SB and JC gave clinical advice. EF wrote the manuscript. All authors provided critical feedback on the manuscript and approved the final version.

Competing interests

The authors declare that they have no competing interests.

References

1. Prince M, Jackson J, al. e. World Alzheimer Report 2009. London: Alzheimer's Disease International; 2009.

2. Prince M, Knapp M, Guerchet M, McCrone P, Prina M, Comas-Herrera A, et al. Dementia UK Update London, UK; 2014.

3. Department of Health. Prime Minister's challenge on dementia: Delivering major improvements in dementia care and research by 2015 2012 [Available from: https://www.gov.uk/government/uploads/system/uploads/attachment_data/file/215101/dh_133176.pdf.

4. Prince M, Bryce R, Ferri C. World Alzheimer Report 2011: The benefits of early diagnosis and intervention: Alzheimer's Disease International; 2011.

5. Leifer BP. Early diagnosis of Alzheimer's disease: clinical and economic benefits. *J Am Geriatr Soc*. 2003;51(5s2):S281-S8.

6. Banerjee S. Living well with dementia—development of the national dementia strategy for England. *Int J Geriatr Psychiatry*. 2010;25(9):917-22.

7. NHS England. Enhanced Service Specification: Dementia Identification Scheme 2014 [Available from: http://www.england.nhs.uk/wp-content/uploads/2014/10/dementia-ident-schm-fin.pdf.

8. NHS England. Dementia diagnosis rate workbooks 2017 [Available from: https://www.england.nhs.uk/publication/dementia-diagnosis-rate-workbook/.

For peer review only - http://bmjopen.bmj.com/site/about/guidelines.xhtml

9. Matthews FE, Stephan BC, Robinson L, Jagger C, Barnes LE, Arthur A, et al. A two decade dementia incidence comparison from the Cognitive Function and Ageing Studies I and II. *Nat Commun*. 2016;7:11398.

10. Department of Health. Living well with dementia: A National Dementia Strategy 2009 [Available from: https://www.gov.uk/government/uploads/system/uploads/attachment_data/file/168220/dh_094051.pdf.

11. Donegan K, Fox N, Black N, Livingston G, Banerjee S, Burns A. Trends in diagnosis and treatment for people with dementia in the UK from 2005 to 2015: a longitudinal retrospective cohort study. *Lancet Public Health*. 2017;2(3):e149-e56.

12. Low L-F, McGrath M, Swaffer K, Brodaty H. Communicating a diagnosis of dementia: A systematic mixed studies review of attitudes and practices of health practitioners. *Dementia*. 2019;18(7-8):2856-905.

13. Lang L, Clifford A, Wei L, Zhang D, Leung D, Augustine G, et al. Prevalence and determinants of undetected dementia in the community: a systematic literature review and a meta-analysis. *BMJ Open*. 2017;7(2):e011146.

14. Moore V, Cahill S. Diagnosis and disclosure of dementia—A comparative qualitative study of Irish and Swedish general practitioners. *Aging & mental health*. 2013;17(1):77-84.

15. Lahjibi-Paulet H, Dauffy A, Minard A, Gaxatte C, Saint-Jean O, Somme D. Attitudes toward Alzheimer's disease: a qualitative study of the role played by social representation on a convenient sample of French general practitioners. *Aging Clin Exp Res*. 2012;24(4):384-90.

16. Dhedhi SA, Swinglehurst D, Russell J. 'Timely'diagnosis of dementia: what does it mean? A narrative analysis of GPs' accounts. *BMJ Open*. 2014;4(3):e004439.

17. Hansen EC, Hughes C, Routley G, Robinson AL. General practitioners' experiences and understandings of diagnosing dementia: factors impacting on early diagnosis. *Soc Sci Med*. 2008;67(11):1776-83.

18. Pentzek M, Wollny A, Wiese B, Jessen F, Haller F, Maier W, et al. Apart from nihilism and stigma: what influences general practitioners' accuracy in identifying incident dementia? *The American journal of geriatric psychiatry : official journal of the American Association for Geriatric Psychiatry*. 2009;17(11):965-75.

For peer review only - http://bmjopen.bmj.com/site/about/guidelines.xhtml

19. Pimlott NJG, Persaud M, Drummond N, Cohen CA, Silvius JL, Seigel K, et al. Family physicians and dementia in Canada Part 2. Understanding the challenges of dementia care. *Can Fam Physician*. 2009;55(5):508-U64.

20. Boise L, Camicioli R, Morgan DL, Rose JH, Congleton L. Diagnosing dementia: Perspectives of primary care physicians. *Gerontologist*. 1999;39(4):457-64.

21. Iliffe S, Manthorpe J, Eden A. Sooner or later? Issues in the early diagnosis of dementia in general practice: a qualitative study. *Fam Pract*. 2003;20(4):376-81.

22. Stephan BC, Tang E, Muniz-Terrera G. Composite risk scores for predicting dementia. *Curr Opin Psychiatry*. 2016;29(2):174-80.

23. The PHG Foundation. Dementia Risk Prediction Models: What do policy makers need to know? Cambridge, UK: The University of Cambridge; 2019.

24. Walters K, Hardoon S, Petersen I, Iliffe S, Omar RZ, Nazareth I, et al. Predicting dementia risk in primary care: development and validation of the Dementia Risk Score using routinely collected data. *BMC Med*. 2016;14(1):1-12.

25. Jammeh EA, Camille BC, Stephen WP, Escudero J, Anastasiou A, Zhao P, et al. Machine-learning based identification of undiagnosed dementia in primary care: a feasibility study. *BJGP open*. 2018;2(2):bjgpopen18X101589.

26. Ford E, Rooney P, Oliver S, Hoile R, Hurley P, Banerjee S, et al. Identifying undetected dementia in UK primary care patients: a retrospective case-control study comparing machine-learning and standard epidemiological approaches. *BMC Med Inform Decis Mak*. 2019;19(1):248.

27. Russell P, Banerjee S, Watt J, Adleman R, Agoe B, Burnie N, et al. Improving the identification of people with dementia in primary care: evaluation of the impact of primary care dementia coding guidance on identified prevalence. *BMJ Open*. 2013;3(12).

28. Wolf A, Dedman D, Campbell J, Booth H, Lunn D, Chapman J, et al. Data resource profile: Clinical Practice Research Datalink (CPRD) Aurum. *Int J Epidemiol*. 2019.

29. Herrett E, Gallagher AM, Bhaskaran K, Forbes H, Mathur R, van Staa T, et al. Data resource profile: clinical practice research datalink (CPRD). *Int J Epidemiol*. 2015;44(3):827-36.

30. Chisholm J. The Read clinical classification. *Brit Med J*. 1990;300:1092.

31. Booth N. What are the Read Codes? *Health Libr Rev*. 1994;11(3):177-82.

32. Rait G, Walters K, Bottomley C, Petersen I, Iliffe S, Nazareth I. Survival of people with clinical diagnosis of dementia in primary care: cohort study. *Brit Med J*. 2010;341:c3584.

For peer review only

For peer review only - http://bmjopen.bmj.com/site/about/guidelines.xhtml

33. Ford E, Carroll JA, Smith H, Scott D, Cassell J. Extracting information from the text of electronic medical records to improve case detection: a systematic review. *J Am Med Inform Assoc*. 2016;doi: 10.1093/jamia/ocv180.

34. Galar M, Fernández A, Barrenechea E, Bustince H, Herrera F. An overview of ensemble methods for binary classifiers in multi-class problems: Experimental study on one-vs-one and one-vs-all schemes. *Pattern Recognition*. 2011;44(8):1761-76.

35. Banerjee S, Willis R, Matthews D, Contell F, Chan J, Murray J. Improving the quality of care for mild to moderate dementia: an evaluation of the Croydon Memory Service Model. *Int J Geriatr Psychiatry*. 2007;22(8):782-8.

36. Kallumpuram S, Kumar CS, Khan B, Gavins V, Khan A, Iliffe S. Targeted case finding for dementia in primary care: Surrey Downs dementia diagnosis project. *BMJ Open Qual*. 2015;4(1):u209827. w4086.

37. Wray LO, Wade M, Beehler GP, Hershey LA, Vair CL. A Program to Improve Detection of Undiagnosed Dementia in Primary Care and Its Association with Healthcare Utilization. *The American journal of geriatric psychiatry : official journal of the American Association for Geriatric Psychiatry*. 2014;22(11):1282-91.

For peer review only

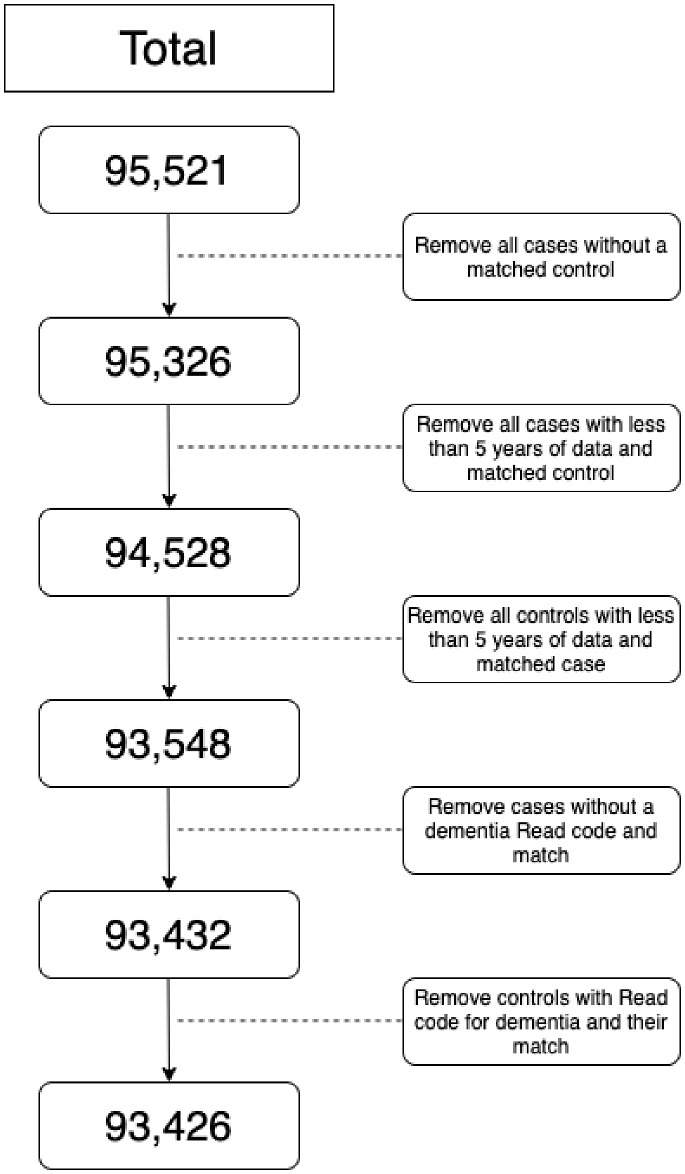

Figure 1: Final Participant Selection.

108x182mm (96 x 96 DPI)

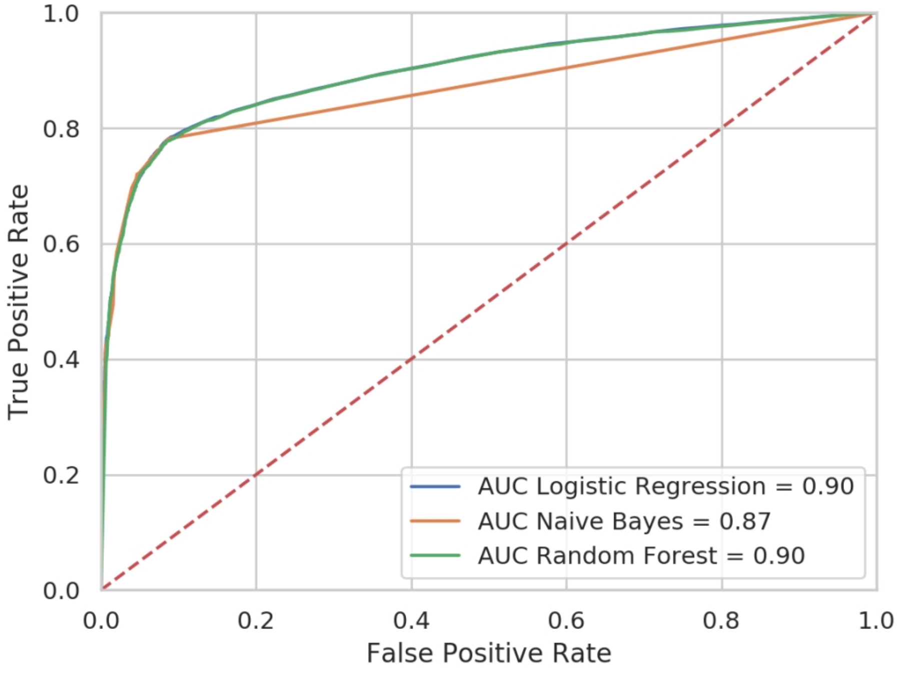

Figure 2: ROC showing discrimination between dementia cases and controls by three machine learning models with codes only.

338x233mm (96 x 96 DPI)

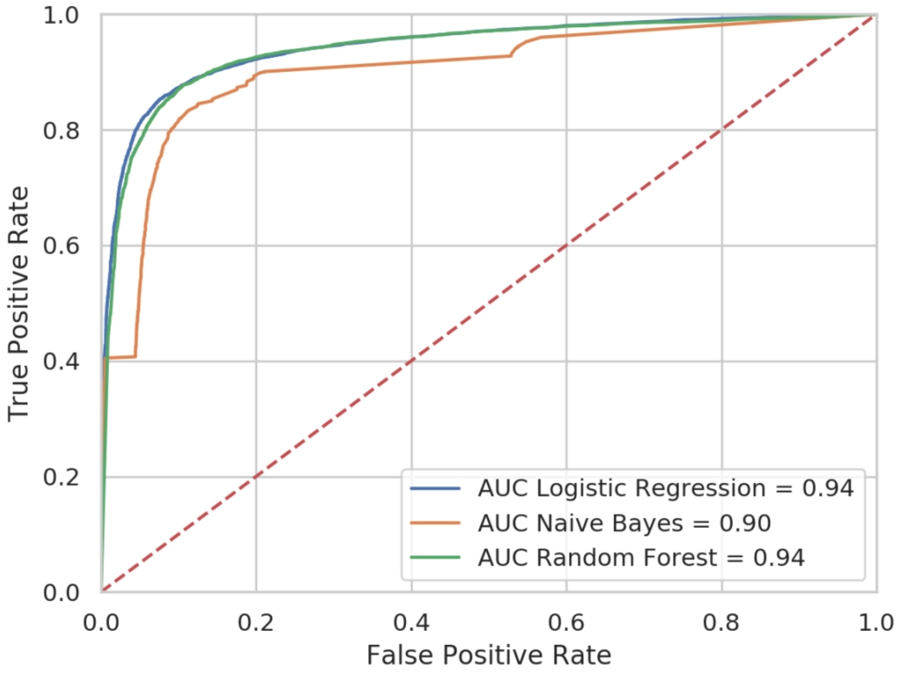

Figure 3: ROC showing discrimination between dementia cases and controls by three machine learning models with both codes and keywords.

338x233mm (96 x 96 DPI)

ANNEX 1 - DEMENTIA DIAGNOSTIC READ CODES

Read Description	Read Code	Medical Code
**Alzheimers codes**		
Dementia in Alzheimer's disease	Eu00	7664
Dementia in Alzheimer's disease with early onset	Eu000	49263
Dementia in Alzheimer's disease with late onset	Eu001	38678
Dementia in Alzheimer's disease, atypical or mixed type	Eu002	30706
Dementia in Alzheimer's disease unspecified	Eu00z	29386
Alzheimer's disease	F110	1917
Alzheimer's disease with early onset	F1100	16797
Alzheimer's disease with late onset	F1101	32057
[X]Presenile dementia,Alzheimer's type	Eu00011	25704
[X]Primary degen dementia, Alzheimer's type, presenile or	Eu00012	60059
[X]Alzheimer's disease type 2	Eu00013	61528
[X]Alzheimer's disease type 1	Eu00111	46762
[X]Senile dementia,Alzheimer's type	Eu00112	11379
[X]Primary degen dementia of Alzheimer's type, senile ons	Eu00113	43346
[X]Alzheimer's dementia unspec	Eu00z11	8195
	15	
**Vascular Dementia Codes**		
Vascular dementia	Eu01	6578
Arteriosclerotic dementia	E004	19477
Multi-infarct dementia	Eu011	8634
Subcortical vascular dementia	Eu012	8934
Mixed cortical and subcortical vascular dementia	Eu013	31016
Other vascular dementia	Eu01y	55313
Vascular dementia unspecified	Eu01z	19393
Uncomplicated arteriosclerotic dementia	Eu0040	43089
Arteriosclerotic dementia with delirium	Eu0041	56912
Arteriosclerotic dementia with paranoia	Eu0042	55467
Arteriosclerotic dementia with depression	Eu0043	43292
Arteriosclerotic dementia NOS	Eu004z	42279
[X]Vascular dementia of acute onset	Eu01000	46488
[X]Predominantly cortical dementia	Eu01111	55838
[X] Multi-infarct dementia	Eu01100	11175
	15	
**Dementia in other diseases**		
[X] Dementia in other diseases classified elsewhere	Eu02y00	64267
Dementia in Pick's disease	Eu020	28402
Circumscribed brain atrophy including Pick's disease	F111	?
Dementia in Creutzfeldt-Jackob disease	Eu021	54106
Dementia in Huntingdon's disease	Eu022	37014
Dementia in Parkinson's disease	Eu023	9509
Dementia in HIV disease	Eu024	4485
Dementia in conditions EC	E041.00	25386
Alcoholic dementia NOS	Eu10711	27342
[X] Alcoholic dementia NOS	Eu10711	26323
Other alcoholic dementia	E012	54505
Drug-induced dementia	E02y100	62132
Lewy body disease	F116	7572
Lewy body dementia	Eu025	26270
	14	
**Dementia not otherwise classified**		
Unspecified dementia	Eu02z	4693
Senile/Presenile dementia	Eu00..12	1350
Presenile dementia	E001	15165

For peer review only

Uncomplicated presenile dementia	E0010	46202
Presenile dementia with delirium	E0011	49513
Presenile dementia with paranoia	E0012	30032
Presenile dementia with depression	E0013	27677
Presenile dementia NOS	E0014	38438
[X] Presenile dementia NOS	Eu02z11	48501
Uncomplicated senile dementia	E000	7323
Senile dementia	E00..11	1916
Senile dementia with depressive or paranoid features	E002	44674
Senile dementia with paranoia	E0020	18386
Senile dementia with depression	E0021	21887
Senile dementia with depressive or paranoid features NOS	E002z	41089
Delirium superimposed on dementia	Eu041	53446
Senile dementia with delirium	E003	37015
Senile degeneration of the brain, not elsewhere classified	F112	29512
	18	
**Total N codes**	**62**	

For peer review only

Cognitive Decline

Read term	Read code	Medcode
Cognitive decline	28E..00	7674
GDS level 2 - very mild cognitive decline	3AE1.00	65856
GDS level 3 - mild cognitive decline	3AE2.00	60263
GDS level 4 - moderate cognitive decline	3AE3.00	60726
GDS level 5 - moderately severe cognitive decline	3AE4.00	70057
GDS level 6 - severe cognitive decline	3AE5.00	94717
GDS level 7 - very severe cognitive decline	3AE6.00	72520
[X]Mild cognitive disorder	Eu05700	11936
[X]Oth & unspec symptom/sign involv cognit funct/awareness	Ryu5100	52939
Health of the Nation Outcome Scale item 4 - cognitive probl	ZRLfE00	35194
Impaired cognition	Z7C1.00	10822
Cognitive function observations	Z7C..00	18906

Cognitive Screening Tests

Read term	Read code	Medcode
Cognitive assessment	311B.00	95853
GPCOG - general practitioner assessment of cognition	38Dv.00	101074
GPCOG (GP assessment of cognition) patient examination	38Dv000	103076
GPCOG (GP assessment of cognition) informant interview	38Dv100	101710
Six item cognitive impairment test	3AD3.00	35305
Cognitions questionnaire	ZR3a.00	36650
Cognitive failures questionnaire	ZR3b.00	66080
Microcog - assessment of cognitive function	ZRa2.00	22257
Neurobehavioural cognitive status examination	ZRaY.00	42059
Rancho scale - levels of cognitive functioning	ZRd..00	92756
Recognition memory test	ZRh6.00	96881

For peer review only

RMT - Recognition memory test	ZRh6.11	93236
Smith cognitive questionnaire	ZRkL.00	96828
Health of the Nation Outcome Scale item 4 - cognitive probl	ZRLfE00	35194
Kendrick cognitive tests for the elderly	ZRV9.11	63604
Lowenstein OT cognitive assessment	ZRVa.00	26264
LOTCA - Lowenstein OT cognitive assessment	ZRVa.11	98256
Measurement of cognitive linguistic ability	ZRVt.00	62114
MCLA - Measurement of cognitive linguistic ability	ZRVt.11	93194

Dementia Annual Review

**Read term**	**Read code**	**Medcode**
Dementia annual review	6AB..00	12710

Memory Loss Codes

**Read term**	**Read code**	**Medcode**
Memory loss - amnesia	1B1A.00	1993
Poor auditory sequential memory	1B1a.00	40821
Amnesia symptom	1B1A.11	3639
Memory loss symptom	1B1A.12	5777
Memory disturbance	1B1A.13	2908
Temporary loss of memory	1B1A000	27788
Short-term memory loss	1B1A100	103453
Transient global amnesia	1B1S.00	28278
Poor visual sequential memory	1B1Y.00	39507
Disturbance of memory for order of events	1S21.00	67163
Forgetful	28G..00	7742
Memory: own age not known	3A10.00	52947
Memory: present time not known	3A20.00	53146
Memory: present place not knwn	3A30.00	53014
Memory: present year not known	3A40.00	52948
Memory: own DOB not known	3A50.00	53125
Memory: present month not knwn	3A60.00	52825
Memory: important event not kn	3A70.00	52800

For peer review only

Memory: import.person not knwn	3A80.00	52801
Memory: count down unsuccess.	3A91.00	52805
Memory: address recall unsucc.	3AA1.00	53016
Patient forgets to take medication	8BIk.00	68703
Non-alcoholic amnestic syndrome	E040.00	27414
Hysterical amnesia	E201700	4269
Mild memory disturbance	E2A1000	6387
Organic memory impairment	E2A1100	6061
[X]Organic amnesic synd not induced alc/oth psychoact subs	Eu03.00	37072
[X]Dissociative amnesia	Eu44000	40994
Transient global amnesia	G655.00	6489
[D]Amnesia (retrograde)	R00z000	4284
[D]Memory deficit	R00z011	7711
[D]Anterograde amnesia	R00z500	54882
[X]Other amnesia	Ryu5000	70677
Memory disturbance (& amnesia (& symptom))	Z7CE400	51379
Memory loss symptom	Z7CE412	67838
Memory loss - amnesia	Z7CE413	103375
Memory disturbance	Z7CE414	105538
Loss of memory	Z7CE415	102880
Forgetful	Z7CE500	24952
Amnesia	Z7CE600	28406
Memory loss	Z7CE611	10123
Memory gone	Z7CE612	68230
Dysmnesia	Z7CE613	66001
Memory loss - amnesia	Z7CE614	12805
Loss of memory	Z7CE615	19297
LOM - Loss of memory	Z7CE616	12277
Transient global amnesia	Z7CE700	18996
TGA - Transient global amnesia	Z7CE711	19004
Anterograde amnesia	Z7CE800	36767
Antegrade amnesia	Z7CE811	43905
Retrograde amnesia	Z7CE900	35538
RA - Retrograde amnesia	Z7CE911	70703
Impairment of registration	Z7CEA00	49897
Impairment of working memory	Z7CEA11	32367
Impairment of immediate recall	Z7CEA12	59844
Impairment of primary memory	Z7CEA13	65696
Amnesia for remote events	Z7CEB00	66144

Poor memory for remote events	Z7CEB12	37191
Amnesia for recent events	Z7CEC00	61816
Loss of memory for recent events	Z7CEC11	9786
No memory for recent events	Z7CEC12	67802
Amnesia for day to day facts	Z7CED00	68392
Amnesia for important personal information	Z7CEE00	57812
Temporary loss of memory	Z7CEF00	67998
Transient memory loss	Z7CEG00	47882
Memory impairment	Z7CEH00	10514
Memory dysfunction	Z7CEH11	39915
Memory deficit	Z7CEH12	50418
Bad memory	Z7CEH13	26434
Memory problem	Z7CEH14	12057
Poor memory	Z7CEH15	12583
Mixes past with present	Z7CEI00	103922
Memory lapses	Z7CEJ00	19073
Minor memory lapses	Z7CEK00	53507
Mild memory disturbance	Z7CEL00	51724
Distortion of memory	Z7CEM00	51739
Invents experiences to compensate for loss of memory	Z7CEN11	64892
Poor short-term memory	Z7CF800	11410
Short-term memory loss	Z7CF811	10571
Unable to recall random address at five minutes	Z7CFA00	106055
Unable to recall five digit number at five minutes	Z7CFC00	104514
Unable to remember name of current prime minister	Z7CFe00	93856
Forgets what was going to do	Z7CFF00	59515
Forgets what was going to say	Z7CFG00	60600
Cannot remember names of intimates	Z7CFg00	105793
Forgets recent activities	Z7CFH00	41366
Cannot remember birth dates of children	Z7CFh00	54430
Forgets what has just done	Z7CFI00	40091
Cannot remember wedding anniversary	Z7CFi00	62749
Forgets what has just said	Z7CFJ00	93319
Forgets what has just read	Z7CFK00	70895
Forgets what has just seen	Z7CFL00	103479
Forgets what has just heard	Z7CFM00	47994
Poor long-term memory	Z7CFO00	53978
Long-term memory loss	Z7CFO11	47581

For peer review only

Unable to remember motor skills	Z7CFq00	46314
Unable to remember own date of birth	Z7CFQ00	63942
Unable to remember own age	Z7CFS00	32599
Unable to remember new motor skills	Z7CFs00	57750
Cannot remember own age	Z7CFS11	69118
Unable to remember day of the week	Z7CFU00	63849
Memory aided by use of diary	Z7CFw00	59830
Unable to remember today's date	Z7CFW00	66684
Memory aided by use of labels	Z7CFx00	98287
Memory aided by use of lists	Z7CFz00	67951
Delayed verbal memory	Z7CGP00	98798

MMSE

**Read term**	**Read code**	**Medcode**
Mini-mental state examination	388m.00	82481
MMSE score	388m.11	83484
Mini mental state score	388V.00	11862
Mini-mental state examination	ZRaA.00	10503
MMSE - Mini-mental state examination	ZRaA.11	10493
Modified mini-mental state 3MS examination	ZRaA100	59387
Modified mini-mental state examination	ZRaA200	36637

Referral to memory assessment services

**Read term**	**Read code**	**Medcode**
Referral to memory clinic	8HTY.00	82481
Seen in memory clinic	9Nk1.00	83484

Referral to Psychiatrist, Neurologist or Geriatrician

**Read term**	**Read code**	**Medcode**
Neurological referral	8H46.00	2964
Geriatric referral	8H47.00	1511
Referral to community geriatrician	8H47000	105235
Psychiatric referral	8H49.00	2189
Referral to psychogeriatrician	8H4D.00	5338
Referral to neurologist	8H4h.00	95859

For peer review only

Referral to neurology special interest general practitioner	8H4Y.00	55362
Psychiatric self-referral	8HJ3.00	27640
Geriatric self-referral	8HJ4.00	68924
Neurology self-referral	8HJE.00	40890
Urgent referral to psychiatrist	8HlB.00	95936
Private referral to neurologist	8HVL.00	13653
Private referral to geriatrician	8HVM.00	29200
Private referral to psychiatrist	8HVO.00	13677
Private referral to psychogeriatrician	8HVS.00	34532
Referral to care of the elderly physician	ZL5A200	12077
Referral to geriatrician	ZL5A211	11634
Referral to neurologist	ZL5AO00	10448
Referral to psychiatrist	ZL5B.00	10002

Alzheimer’s prescription Product Codes (specific to CPRD)

7329
36976
33007
56771
11751
53922
10255
38976
33008
33009
60723
56600
11752
11635
48443
53882
2931
6225
63217
11654
56631
39240
63951
11827
35088
60107
10187
58969
59871
2930

For peer review only

35179
7361
4597
61676
58947
14309
11837
58709
9786
37132
48442

For peer review only

**Keyword**	**Stem**	**misspellings**	
**Memory**	memory		
	memori*	memroy	
		memeory	
		memmory	
		meory	
		memort	
		memrory	
		memorry	
**cognition**	cognition	cogition	
	cognitive	cogntion	
	cognit*	cngition	
		congtion	
		cogniton	
		congit*	
		cgnit*	
**forgetfulness**	forgetting	foget*	
	forgetful*	forgot*	
	forget*	forgte	
		fforget*	
		froget*	
**confusion**	confusion	confudes	
	confused	condusef	
	confus*	cofnused	
**dementia**	dementia		
	dement*		
	Alzheimer's	alzheimers	
		alzhemer	
		alzhiemer	
		alzhiemers	
		alzhimer	
		alzhimers	
		alzimer	
		alz*	
**behaviour change**	behaviour	behavour	
	behavior	bahaviour	
	behav*	bahavior	
		bahavioe	
		behavoir	
		behaveour	
**family concerned**	family + concerned	**for family substitute:**	**for concerned substitute**
		relative	conserned
		wife	concern*
		husband	worried
		son	
		daughter	
		partner	
		spouse	
		niece	

For peer review only

		nephew
		sister
		brother
		relative
		neighbour
**3rd Party**	third party consultation	third party consulation
		3rd party consultation
		3rd party consulation
**MMSE**	MMSE	
	mini mental state	
	mini-mental state	
	GP Cog	

For peer review only

The RECORD statement – checklist of items, extended from the STROBE statement, that should be reported in observational studies using routinely collected health data.

	Item No.	STROBE items	Location in manuscript where items are reported	RECORD items	Location in manuscript where items are reported
**Title and abstract**					
	1	(a) Indicate the study's design with a commonly used term in the title or the abstract (b) Provide in the abstract an informative and balanced summary of what was done and what was found		RECORD 1.1: The type of data used should be specified in the title or abstract. When possible, the name of the databases used should be included.<br><br>RECORD 1.2: If applicable, the geographic region and timeframe within which the study took place should be reported in the title or abstract.<br><br>RECORD 1.3: If linkage between databases was conducted for the study, this should be clearly stated in the title or abstract.	Title and abstract<br><br>Abstract<br><br>N/A
**Introduction**					
Background rationale	2	Explain the scientific background and rationale for the investigation being reported			4
Objectives	3	State specific objectives, including any prespecified hypotheses			6
**Methods**					
Study Design	4	Present key elements of study design early in the paper			6
Setting	5	Describe the setting, locations, and relevant dates, including periods of recruitment, exposure, follow-up, and data collection			6

For peer review only

Participants	6	*(a) Cohort study* - Give the eligibility criteria, and the sources and methods of selection of participants. Describe methods of follow-up<br>*Case-control study* - Give the eligibility criteria, and the sources and methods of case ascertainment and control selection. Give the rationale for the choice of cases and controls<br>*Cross-sectional study* - Give the eligibility criteria, and the sources and methods of selection of participants<br><br>*(b) Cohort study* - For matched studies, give matching criteria and number of exposed and unexposed<br>*Case-control study* - For matched studies, give matching criteria and the number of controls per case	7<br><br>7	RECORD 6.1: The methods of study population selection (such as codes or algorithms used to identify subjects) should be listed in detail. If this is not possible, an explanation should be provided.<br><br>RECORD 6.2: Any validation studies of the codes or algorithms used to select the population should be referenced. If validation was conducted for this study and not published elsewhere, detailed methods and results should be provided.<br><br>RECORD 6.3: If the study involved linkage of databases, consider use of a flow diagram or other graphical display to demonstrate the data linkage process, including the number of individuals with linked data at each stage.	7 (Appendix 1)<br><br>N/A<br><br>N/A
Variables	7	Clearly define all outcomes, exposures, predictors, potential confounders, and effect modifiers. Give diagnostic criteria, if applicable.	8	RECORD 7.1: A complete list of codes and algorithms used to classify exposures, outcomes, confounders, and effect modifiers should be provided. If these cannot be reported, an explanation should be provided.	8 and Supplementary Materials (Appendix 2 & 3)
Data sources/ measurement	8	For each variable of interest, give sources of data and details of methods of assessment (measurement).<br>Describe comparability of assessment methods if there is more than one group	8		

For peer review only

Bias	9	Describe any efforts to address potential sources of bias	7		
Study size	10	Explain how the study size was arrived at	7		
Quantitative variables	11	Explain how quantitative variables were handled in the analyses. If applicable, describe which groupings were chosen, and why	8-9		
Statistical methods	12	(a) Describe all statistical methods, including those used to control for confounding<br>(b) Describe any methods used to examine subgroups and interactions<br>(c) Explain how missing data were addressed<br>(d) *Cohort study* - If applicable, explain how loss to follow-up was addressed<br>*Case-control study* - If applicable, explain how matching of cases and controls was addressed<br>*Cross-sectional study* - If applicable, describe analytical methods taking account of sampling strategy<br>(e) Describe any sensitivity analyses	8<br>N/A<br>Missing data were not addressed<br>N/A<br>7<br>N/A<br>No sensitivity analyses		
Data access and cleaning methods		..		RECORD 12.1: Authors should describe the extent to which the investigators had access to the database population used to create the study population.	6-7

For peer review only

				RECORD 12.2: Authors should provide information on the data cleaning methods used in the study.	7 and Figure 1
Linkage		..		RECORD 12.3: State whether the study included person-level, institutional-level, or other data linkage across two or more databases. The methods of linkage and methods of linkage quality evaluation should be provided.	7: No data linkage.
**Results**					
Participants	13	(a) Report the numbers of individuals at each stage of the study (*e.g.*, numbers potentially eligible, examined for eligibility, confirmed eligible, included in the study, completing follow-up, and analysed)<br>(b) Give reasons for non-participation at each stage.<br>(c) Consider use of a flow diagram	Figure 1 and page 7	RECORD 13.1: Describe in detail the selection of the persons included in the study (*i.e.,* study population selection) including filtering based on data quality, data availability and linkage. The selection of included persons can be described in the text and/or by means of the study flow diagram.	Figure 1 and page 7
Descriptive data	14	(a) Give characteristics of study participants (*e.g.*, demographic, clinical, social) and information on exposures and potential confounders<br>(b) Indicate the number of participants with missing data for each variable of interest<br>(c) *Cohort study* - summarise follow-up time (*e.g.*, average and total amount)	9<br><br>Missingness not assessed because of confounding of missing with negative values.		
Outcome data	15	*Cohort study* - Report numbers of outcome events or summary measures over time<br>*Case-control study* - Report numbers in each exposure	 <br>9 and Table 1		

For peer review only

		category, or summary measures of exposure<br>*Cross-sectional study* - Report numbers of outcome events or summary measures			
Main results	16	(a) Give unadjusted estimates and, if applicable, confounder-adjusted estimates and their precision (e.g., 95% confidence interval). Make clear which confounders were adjusted for and why they were included<br>(b) Report category boundaries when continuous variables were categorized<br>(c) If relevant, consider translating estimates of relative risk into absolute risk for a meaningful time period	a) Relative estimates of feature weighting in the classification given in Table 3.<br>b) N/A<br>c) N/A		
Other analyses	17	Report other analyses done—e.g., analyses of subgroups and interactions, and sensitivity analyses	N/A		
**Discussion**					
Key results	18	Summarise key results with reference to study objectives	12		
Limitations	19	Discuss limitations of the study, taking into account sources of potential bias or imprecision. Discuss both direction and magnitude of any potential bias	13-14	RECORD 19.1: Discuss the implications of using data that were not created or collected to answer the specific research question(s). Include discussion of misclassification bias, unmeasured confounding, missing data, and changing eligibility over time, as they pertain to the study being reported.	
Interpretation	20	Give a cautious overall interpretation of results considering objectives,	14-15		

For peer review only

		limitations, multiplicity of analyses, results from similar studies, and other relevant evidence			
Generalisability	21	Discuss the generalisability (external validity) of the study results	14		
**Other Information**					
Funding	22	Give the source of funding and the role of the funders for the present study and, if applicable, for the original study on which the present article is based	15		
Accessibility of protocol, raw data, and programming code		..		RECORD 22.1: Authors should provide information on how to access any supplemental information such as the study protocol, raw data, or programming code.	Protocol will be supplied on application to authors.

*Reference: Benchimol EI, Smeeth L, Guttmann A, Harron K, Moher D, Petersen I, Sørensen HT, von Elm E, Langan SM, the RECORD Working Committee. The REporting of studies Conducted using Observational Routinely-collected health Data (RECORD) Statement. *PLoS Medicine* 2015; in press.

*Checklist is protected under Creative Commons Attribution (CC BY) license.

For peer review only

BMJ Open

Automated detection of patients with dementia whose symptoms have been identified in primary care but have no formal diagnosis: a retrospective case-control study using electronic primary care records

Journal:	*BMJ Open*
Manuscript ID	bmjopen-2020-039248.R1
Article Type:	Original research
Date Submitted by the Author:	29-Sep-2020
Complete List of Authors:	Ford, Elizabeth; Brighton and Sussex Medical School, Department of Primary Care and Public Health<br>Sheppard, Joanne; University of Sussex School of Mathematical and Physical Sciences, Department of Physics and Astronomy; UCL, Medical Physics and Biomedical Engineering<br>Oliver, Seb; University of Sussex School of Mathematical and Physical Sciences, Department of Physics and Astronomy<br>Rooney, Philip; University of Sussex School of Mathematical and Physical Sciences, Department of Physics and Astronomy<br>Banerjee, Sube; University of Plymouth, Faculty of Health<br>Cassell, Jackie; Brighton and Sussex Medical School, Department of Primary Care and Public Health
**Primary Subject Heading**:	Health informatics
Secondary Subject Heading:	Diagnostics, General practice / Family practice, Geriatric medicine, Neurology
Keywords:	Dementia < NEUROLOGY, Health informatics < BIOTECHNOLOGY & BIOINFORMATICS, GERIATRIC MEDICINE, PRIMARY CARE, Delirium & cognitive disorders < PSYCHIATRY, Old age psychiatry < PSYCHIATRY

SCHOLARONE™
Manuscripts

I, the Submitting Author has the right to grant and does grant on behalf of all authors of the Work (as defined in the below author licence), an exclusive licence and/or a non-exclusive licence for contributions from authors who are: i) UK Crown employees; ii) where BMJ has agreed a CC-BY licence shall apply, and/or iii) in accordance with the terms applicable for US Federal Government officers or employees acting as part of their official duties; on a worldwide, perpetual, irrevocable, royalty-free basis to BMJ Publishing Group Ltd ("BMJ") its licensees and where the relevant Journal is co-owned by BMJ to the co-owners of the Journal, to publish the Work in this journal and any other BMJ products and to exploit all rights, as set out in our licence.

The Submitting Author accepts and understands that any supply made under these terms is made by BMJ to the Submitting Author unless you are acting as an employee on behalf of your employer or a postgraduate student of an affiliated institution which is paying any applicable article publishing charge ("APC") for Open Access articles. Where the Submitting Author wishes to make the Work available on an Open Access basis (and intends to pay the relevant APC), the terms of reuse of such Open Access shall be governed by a Creative Commons licence – details of these licences and which Creative Commons licence will apply to this Work are set out in our licence referred to above.

Other than as permitted in any relevant BMJ Author's Self Archiving Policies, I confirm this Work has not been accepted for publication elsewhere, is not being considered for publication elsewhere and does not duplicate material already published. I confirm all authors consent to publication of this Work and authorise the granting of this licence.

For peer review only

Automated detection of patients with dementia whose symptoms have been identified in primary care but have no formal diagnosis: a retrospective case-control study using electronic primary care records

Elizabeth Ford[1]
Joanne Sheppard [2,3]
Seb Oliver[2]
Philip Rooney[2]
Sube Banerjee[4]
Jackie Cassell[1]

1) Department of Primary Care and Public Health, Brighton and Sussex Medical School
2) Department of Physics and Astronomy, University of Sussex.
3) Medical Physics and Biomedical Engineering, University College London
4) Faculty of Health, University of Plymouth

Corresponding Author:
Elizabeth Ford
Department of Primary Care and Public Health, Brighton and Sussex Medical School
Watson Building, Village Way, Falmer
Brighton BN1 9PH
Tel: 01273 641974 email: e.m.ford@bsms.ac.uk

Keywords
Dementia; early diagnosis; primary care; electronic health records; machine learning; free text.

For peer review only

Abstract

Objectives: United Kingdom (UK) statistics suggest only two-thirds of patients with dementia get a diagnosis recorded in primary care. General practitioners (GPs) report barriers to formally diagnosing dementia, so some patients may be known by the GP to have dementia but missing a diagnosis in their patient record. We aimed to produce a method to identify these “known but unlabelled” patients with dementia using data from primary care patient records.

Design: Retrospective case-control study using routinely collected primary care patient records from CPRD.

Setting: UK general practice.

Participants: English patients aged >65y, with a coded diagnosis of dementia recorded 2000-2012 (cases); matched 1:1 to patients with no diagnosis code for dementia (controls).

Interventions: 8 coded and 9 keyword concepts indicating symptoms, screening tests, referrals and care for dementia recorded in the 5 years before diagnosis. We trialled machine learning classifiers to discriminate between cases and controls (logistic regression, naïve Bayes, random forest).

Primary and Secondary Outcomes: Outcome variable was dementia diagnosis code; accuracy of classifiers was assessed using Area Under the Receiver Operating Characteristic (AUC); order of features contributing to discrimination was examined.

Results: 93,426 patients were included; median age 83 years (64.8% female). Three classifiers achieved high discrimination and performed very similarly. AUCs were 0.87-0.90 with coded variables rising to 0.90-0.94 with keywords added. Feature prioritisation was different for each classifier, commonly prioritised features were Alzheimer’s prescription, dementia annual review, memory loss, and dementia keywords.

Conclusions: It is possible to detect patients with dementia who are known to GPs but unlabelled with a diagnostic code, with a high degree of accuracy in electronic primary care record data. Using keywords from clinic notes and letters improves accuracy compared to coded data alone. This approach could improve identification of dementia cases for record-keeping, service planning, and delivery of good quality care.

For peer review only

For peer review only - http://bmjopen.bmj.com/site/about/guidelines.xhtml

Strengths and Limitations of this Study:

- Our study was representative of the UK population, using all patients in the CPRD GOLD database who were over 65 years old and had a first recorded dementia diagnosis in 2000-2012 (N= 46,713).
- However, the data used is several years old and there may have been changes in clinical practice since.
- We trialled a number of binary classifiers, but combining machine learning approaches might have increased the accuracy achieved.
- The approach was additionally strengthened by the addition of keywords from clinic notes and text to coded information.
- A limitation is that our model was trained on cases who all received a dementia diagnosis code at the end of the study period, who may differ systematically from those who never receive a code.

For peer review only

Introduction

Dementia is a term used to describe a group of conditions characterised by a progressive decline in cognitive function and consequent functional limitation.[1] In the United Kingdom (UK), 1.3% of people live with dementia, the majority of whom are over 65 years. Prevalence in all over 65s is 7.1%, rising from 1.8% in the 65-69 age group to over 33% in the over 90s.[2] The challenges of dementia affect individuals, their family carers, health and care services and systems and society as a whole, and addressing them has become a national and international priority due to population ageing. It is expected that the cost of the disease to society will double between 2014 and 2040, reaching £55bn per year in the UK.[2]

The UK government has recognised the benefits of an early diagnosis of dementia and has made improving rates of diagnosis a key focus of dementia policy since 2009 with the National Dementia Strategy 2009 [3] and the Prime Minister's Challenges on Dementia in 2012 and 2020.[4, 5] Whilst the disease is incurable, a diagnosis gives the patient a better chance of achieving a good quality of life while living with dementia.[6] Individuals can plan and make their wishes known while they still have capacity. Services provided by health and social care professionals may help people with dementia to adapt their home and identify their care needs, and thus avoiding or delaying having to move into institutionalised care. Furthermore, diagnosis allows people with Alzheimer's disease to be prescribed medication and non-pharmaceutical treatments to maximise their cognitive function and quality of life. These treatments may have a particular benefit in the early stages of the illness.[7, 8]

In recognition of this, general practitioners (GPs) in the UK are incentivised to initiate the diagnosis of dementia and to keep a register of patients diagnosed with dementia so that they can provide care proactively.[9] GPs are likely to be the first to recognise or be consulted about dementia symptoms, as 98% of the UK population is registered with a GP to receive primary healthcare services. If GPs suspect dementia they refer the patient to a specialist memory assessment service where a full diagnostic screening is carried out. If the patient is diagnosed with dementia, this is communicated back to the GP who then continues the patient's care. GPs are reimbursed for ensuring a face-to-face review for each patient with dementia every 12 months. Data collected in GP patient records about dementia cases (using a prescribed set of Dementia Read codes) is used by local Clinical Commissioning Groups (CCGs) as well as national bodies such as NHS England and Public Health England to monitor local and national diagnosis rates and services.[10]

For peer review only - http://bmjopen.bmj.com/site/about/guidelines.xhtml

For peer review only

Based on an epidemiological study conducted by the Medical Research Council in 1998 a prevalence of 6.6% was estimated in people over the age of 65.[11] However, based on dementia registers in the UK, the observed prevalence was found to be 3%.[11] This gap has closed substantially, due to schemes like the National Dementia Strategy,[3, 12] but coded diagnoses of dementia in GP patient records still only identify two-thirds of cases, compared to the expected prevalence,[10, 13] and the estimated median time to diagnosis being recorded from dementia onset is 3.5 years.[14] While the diagnosis rate in the UK is higher than in many countries (for example Lang et al.,[15] found an average rate of undetected dementia of 62% across Europe, North America and Asia) a third of people with dementia may not be receiving appropriate care.

Qualitative studies with GPs have indicated that GPs are sometimes reluctant to diagnose dementia,[16-18] and describe dementia as "a complex condition that takes time to diagnose".[19] GPs have reported that diagnosing dementia early was not particularly important, and may in fact be harmful to some patients.[19] GPs tread carefully when initiating and communicating a diagnosis of dementia to their patients, as it is a stigmatised condition with what seems a bleak prognosis.[18, 20] Some researchers have argued that there is not enough evidence for any intervention that provides a positive change in prognosis or well-being for individuals with dementia and therefore a drive for earlier diagnosis is not justified.[21] In addition, as elderly patients often have multiple conditions, GPs report that it is difficult to integrate multiple clinical guidelines,[22] and that "dementia or memory problems [are] right at the bottom of the list".[23] These findings suggest that patients with undiagnosed dementia could fall into two groups. First, those whose memory loss or cognitive symptoms have not been identified by the GP, possibly because they do not present to the GP very often, or they have not communicated any memory related symptoms. Second, there is likely to be a group of patients who are known by primary care clinicians to be experiencing memory problems, and may even have been screened for dementia or referred for memory assessment, but who have not, for many possible reasons, received a diagnostic code for dementia in their GP record.[16, 20, 24] For these patients who are "known but unlabelled", there may be other indicators of dementia recognition and diagnosis in the patient record which could enable them to be detected using an algorithm which combines multiple indicators of the disease. Following identification, the GP could then decide if "labelling" these patients with a dementia code was appropriate.

For peer review only - http://bmjopen.bmj.com/site/about/guidelines.xhtml

Reviews of data-driven dementia risk prediction models,[25, 26] show that the majority of work in the past has aimed at producing risk scores which estimate the patient's risk of developing dementia at some future date. Only a few of these risk scores have been developed on routinely collected primary care data of which, a notable example is Walters et al.[27] Automated early detection of emergent cases has received less attention. Few attempts have been made to produce models which detect dementia which is current but unrecognised by the GP; the only examples using UK primary care data (known to the authors) are currently Jammeh et al.,[28] and Ford et al.[29, 30] Other studies have approached this problem manually, for example Russell et al.,[31] aimed to improve recording in dementia registers by a hand-searching exercise, recognising the need to "'clean up dementia coding and records". This successful exercise increased the identification rate for dementia in participating GP practices by 8.8%.

Following success in detecting unrecognised patients with dementia,[29] in this study we aimed to identify a range of elements in the primary care record which would identify the second group of "known but unlabelled" patients with dementia. A second aim was to evaluate whether the model could be improved by the use of keyword information in clinic notes and letters found in the patient records, rather than relying solely on information captured in clinical codes. Automatically detecting these patients may help with improving the quality of data in dementia registers, enabling ongoing care and support of individual patients with dementia, and giving commissioning bodies and service planners more accurate prevalence estimates.

Methods

Study Design

A retrospective case-control study using routinely collected primary care patient records from England, reported according to RECORD guidelines.[32]

Data Source

This study used data from the UK Clinical Practice Research Datalink (CPRD). It was established in 1987, and now contains anonymized healthcare records from more than 7 million current patients and represents up to 13% of the UK population.[33, 34] Patients are broadly representative of the UK general population in terms of age, sex and ethnicity. CPRD includes longitudinal observational data from general practitioner (GP) electronic health record systems in primary care practices, including

For peer review only - http://bmjopen.bmj.com/site/about/guidelines.xhtml

medical diagnoses, referrals to specialists and to secondary care, testing and interventional procedures conducted in primary care, lifestyle information (e.g. smoking, exercise) and drugs prescribed in primary care.[34] Data are captured using a structured hierarchical vocabulary called Read Codes, these were developed by a UK GP, Dr James Read, in the 1980s.[35] They map to other nomenclatures such as ICD, SNOMED-CT, and International Classification of Primary Care (ICPC) codes. Each Read code represents a term or short phrase describing a health-related concept. There are over 200,000 different codes, which are sorted into categories (diagnoses, processes of care and medication) and subchapters.[36] Each clinical entity is represented by an alphanumeric code and a Read term which is the plain language description. CPRD curates two primary care databases, one is formed of patient records derived from a GP patient record computer software called VISION (CPRD GOLD) and one is formed from records derived from a GP patient record computer software called EMIS (CPRD AURUM) [33]. This study used only CPRD GOLD data.

GPs also record clinical information in free text clinic notes and letters. CPRD held these clinic notes at the time of study data extraction, and made these available via either keyword search or release of full text for small samples, following 3-stage manual de-identification of text and approval of a protocol. Since 2013 free text notes and letters have not been extracted into CPRD from GP practices, and historic clinic text became unavailable to researchers in 2016. Due to this cessation of free text collection, the current study used data for patients who had a first dementia diagnosis recorded up to the end of 2012, so that free text was available for the full period prior to their diagnosis CPRD also offers data linkages with other NHS and administrative datasets but no linked data were used in this study.

Study Population

CPRD provided the study team with longitudinal data from patients with a dementia code (cases) recorded between 2000-2012, as defined in a code list of dementia diagnostic codes (general dementia, vascular and Alzheimer's dementia codes) that we developed drawing on code lists in Russell et al.,[31] and Rait et al.,[37] and reported in Ford et al.[29] (Appendix 1) For data extraction by the CPRD team, patients were required to be 65 years or older, and were required to have records available for at least three years prior to a first diagnosis code recorded between 2000 and 2012. All patients in the CPRD GOLD database meeting these criteria were selected to maximise sample size and reduce the possibility of selection bias. Control patients were randomly sampled from patients who had no dementia codes anywhere in their record (to reduce the likelihood that they just had a delayed diagnosis), who matched

For peer review only - http://bmjopen.bmj.com/site/about/guidelines.xhtml

cases on age, sex, and general practice, and who also had a minimum of three years' of records before the diagnosis date of their matched case (known as the "index date"). This resulted in a near-perfect 1-to-1 match between cases and controls, a few cases did not receive a match. No minimum duration of follow up after index date was required, as we did not want to bias the sample towards healthier individuals or exclude those who moved home or went into residential care (and thus changed GP practice) during follow up. Thus patients could have a date of death during the study as long as this was after the index date. Data collection on all patients was limited to November 2013 as this was the date of the final CPRD Gold database build which included free text. This resulted in 47,858 cases and 47,663 controls.

The entire coded patient record was extracted for each patient, including all clinical codes, from the patient's earliest registration date in CPRD until the date of data extraction (November 2013), or the patient's date or death or the date they left the practice, whichever was earlier. In addition, CPRD ran a search through free text notes and letters for keywords for all patients (described below) in the 24 months prior to the index date. For the keyword search, CPRD returned structured data comprised of a count of the keywords in each keyword group, found in each free text entry in the record. The study team did not have access to the free text of any patient for this study.

Refining participants

To standardise the dataset provided by CPRD, and to optimise it for the planned analysis, further refinements were made to the sample. From the extracted dataset, cases without matched controls, and all patients lacking five years of data prior to index date were removed along with their corresponding match. We removed cases for whom we could not find a dementia read code within the study period, and controls who did have a dementia code, and removed these along with their matched patients. See Figure 1 for a flowchart of final participant selection.

- insert Figure 1 here -

Legend: Figure 1: Final Participant Selection.

Feature selection

Code lists:

For peer review only - http://bmjopen.bmj.com/site/about/guidelines.xhtml

Using an expert consensus approach, with an epidemiologist, a psychiatrist and a GP, we identified a list of 8 potential clinical concepts which would be indicators of the onset of dementia, initiation of the diagnostic pathway for dementia or evidence of continuing care for dementia, and which would be captured within the Read codes. Our final list of eight coded concepts entered into each model were: cognitive decline, memory loss symptoms, mini-mental state examination (MMSE), other cognitive screening test, referral to memory clinic, referral to psychiatrist, neurologist or geriatrician, dementia annual review, and Alzheimer's specific prescription codes. Relevant code lists were sought from published papers, and where these were not available, lists were drawn up by author EF and checked by author PR (Appendix 2). Although MMSE scores are sometimes recorded in structured data, we found a majority of scores were missing in our dataset (56%). We decided to keep the model as simple as possible and therefore did not use MMSE scores in the analysis.

Keywords from clinic notes and letters:

We consulted with 17 GPs, asking them to suggest keywords representing terms that they might use when considering that a patient might be developing dementia. From these we identified 9 concepts: memory (suggested by 17 GPs); cognition (6 GPs); forgetful (14 GPs) MMSE or other screening test (6 GPs); confused (9 GPs); dementia (3 GPs); behaviour (5 GPs); family concern (6 GPs) and third party consultation (1 GP). We then generated a list of concept variations and spelling variations for each keyword group (Appendix 3).

Analysis

Only data from 5 years before the index date, up to the index date for each patient was used. Each patient's record was searched to find all instances of the Read codes referring to the eight features described above. If any codes were found for a feature within a patient's records, a dummy variable representing that feature was set to "1" otherwise they were defaulted to "0". Likewise, if evidence of keyword was found, the dummy variable for keywords were set to "1". A dummy variable for "any keyword" was also included. Other studies have also favoured binary features in order to reduce the influence on the data of the number of times a patient visits their GP.[28]

Logistic regression, random forest, and naive Bayes classifiers were used as they are effective supervised machine learning methods for the analysis and classification of binary data. Logistic regression uses the logit function to find the probability of a data point belonging to a (binary) group, using one or more nominal, ordinal, interval or ratio-level independent variables.[38] The addition of the LASSO function

For peer review only - http://bmjopen.bmj.com/site/about/guidelines.xhtml

selects the best predictors based on the residual sum of squares [39]. The random forest method is a non-parametric machine learning method for binary classification. It constructs a multitude of decision trees that best use the predictors to determine which category of output each case should be in. It holds the multitude of decision trees constant and the output for each individual is the mode or mean prediction class of the individual trees.[40] Naïve Bayes classifiers are based on Bayes Theorem.[41] Parameter estimation for naive Bayes models uses the method of maximum likelihood, and all predictive features are treated as contributing independently of each other (i.e. they are naïve to each other) to predict which category of the outcome each case belongs to.[42] These were implemented by using sci-kit learn with Python version 3.7.1 with the "LogisticRegression", "BernoulliNB" and "RandomForestClassifer" packages, with the seed set to 42 for reproducibility. The dataset was split into a 70% set for training the classifiers and a 30% testing set to assess model accuracy.

To assess the accuracy of the models the Area Under the Receiver Operator Curve (AUC) was calculated, which plots the true positive rate against the false positive rate for every possible cut-off of the predictive model. In addition, sensitivity and specificity values were calculated for model cut-offs where these values were equally weighted. Positive predictive values (PPV) were also calculated. While sensitivity and specificity are fixed parameters of the test, PPV values are conditional on the prevalence of the condition in the tested sample. Given the matched case-control design, our sample had a dementia prevalence of 50%, which would inflate PPV values artificially. We therefore separately calculated the PPV of the model using the estimated UK prevalence of dementia in people over 65 years of 7.1%.[2] This was achieved using the values of sensitivity and specificity calculated above, and a hypothetical sample of 100,000 patients where 7100 had dementia.

To assess which features contributed most to the models, we generated relative indices, with the most predictive feature weighting set at 1, and other features shown relative to this. This was instead of reporting the exact outputs from each machine learning algorithm (such as β weights from logistic regression), because we wanted models to be directly compared.

Results

Study Participants

For peer review only

The final dataset consisted of 93,426 patients with a median age of 83 years old (range 65-110). There were 32,876 male participants (35.2%) with a median age of 81 years old (range 65-100), and 60,550 female participants (64.8%) with a median age of 83 years old (range 65-110). 46,713 were cases and 46,713 were controls. The mean number of codes recorded per patient in the 5 years prior to index date was 614 for the controls and 586 for the cases (range 0-7,281).

Numbers of features by case and control group

Cases had much higher rates of all chosen features, as can be seen in Table 1. The most common coded feature in dementia cases was memory loss codes (46%), followed by dementia annual review codes (37%). Keywords were all common throughout cases, the most frequent were family concern (81%), memory (58%) and dementia (51%). All keywords were more common in cases than controls, except for third party consultation, and family concern was also high in controls (51%), although not as high as in the cases.

Table 1: Number and proportion of cases and controls who had each feature.

Feature	N cases with features (%) (N = 46,713)	N controls with feature (%) (N = 46,713)
**Codes**		
Cognitive decline codes	3,276 (7.01)	240 (0.51)
Cognitive screening test	888 (1.90)	124 (0.27)
Dementia annual review	17,372 (37.19)	151 (0.32)
Memory loss codes	21,282 (45.56)	1,387 (2.97)
MMSE test	9,574 (20.32)	600 (1.28)
Referral to memory assessment service	3,464 (7.42)	117 (0.25)
Referral to old age psychiatrist, neurologist or geriatrician	12,683 (27.15)	2,227 (4.77)
Alzheimer's medication prescription	10,044 (21.50)	203 (0.43)
**Keywords**		
Memory	27,025 (57.85)	3,815 (8.17)
Cognition	8,873 (18.99)	2,178 (4.66)
Forgetful	14,272 (30.55)	3,397 (7.27)
MMSE or screening test	11,969 (25.62)	2,189 (4.69)
Confused	20,530 (43.95)	5,334 (11.42)
Dementia	23,935 (51.24)	2,848 (6.10)
Behaviour change/problems	5,388 (11.53)	2,132 (4.56)
Family concerned	37,779 (80.87)	23,718 (50.77)
Third party consultation	706 (1.51)	1,854 (3.97)

Model Accuracy

For peer review only - http://bmjopen.bmj.com/site/about/guidelines.xhtml

The eight coded clinical concepts were used to train the three classifiers to predict which patients' record would end with a dementia diagnosis, and which patients' would not. Trained models were then tested on the testing dataset and accuracy of discrimination was assessed. Using only coded variables, the three models produced a high level of correct discrimination between cases and controls. The three classifiers performed very similarly with AUCs of 0.87-0.90 (Table 2 and Figure 2).

We then added the nine keywords to the code-only models, and found that discrimination improved up to an AUC of 0.94 in the logistic regression and random forest and 0.90 in the naïve Bayes classifier (Figure 3). It can be seen in Table 2 that the inclusion of the keywords increased the sensitivity of the models by around 8%, without any detrimental effect on specificity.

Table 2: Discrimination of three models between cases and controls

	Codes only				Codes and Keywords			
Classifier	AUC	Sensitivity	Specificity	PPV at 7.1% prevalence	AUC	Sensitivity	Specificity	PPV at 7.1% prevalence
Random Forest	0.90	0.77	0.92	0.46	0.94	0.85	0.92	0.46
Logistic Regression	0.90	0.76	0.93	0.49	0.94	0.84	0.93	0.51
Naive Bayes	0.87	0.78	0.91	0.45	0.90	0.80	0.91	0.43

- insert Figure 2 here -

Legend: Figure 2: ROC showing discrimination between dementia cases and controls by three machine learning models with codes only.

- insert Figure 3 here -

Legend: Figure 3: ROC showing discrimination between dementia cases and controls by three machine learning models with both codes and keywords.

Feature weighting

The relative weighting of each feature in the different models, with the most predictive feature weighting set at 1, and other features shown relative to this, is shown in Table 3, separated by models for the coded data only and for codes and keywords together. The table shows that the three different models had substantial differences in the weighting of the different features when using only keywords.

For peer review only - http://bmjopen.bmj.com/site/about/guidelines.xhtml

The logistic regression weighted most highly dementia annual review codes and prescriptions of Alzheimer's medication, while the random forest weighted memory loss codes highest and the naïve Bayes classifier weighted referral for memory assessment as the most informative feature.

Looking at the combined codes and keywords, similar codes were most important in the three models, whereas the most important keyword was the word memory (rated highest feature in the random forest model), followed by keywords for dementia. Interestingly, the logistic regression classifier prioritised third party consultation, the only feature higher in the controls than the cases. The naïve Bayes classifier prioritised coded variables higher than all keywords.

Table 3: Relative weighting of each feature in the three classifiers

	Coded data only			Codes and Keywords		
	Logistic Regression	Random Forest	Naive Bayes	Logistic Regression	Random Forest	Naive Bayes
Alzheimer's Medication	0.727	0.354	0.912	0.676	0.339	0.912
Cognitive Decline	0.378	0.066	0.888	0.260	0.061	0.880
Cognitive Screening Test	0.025	0.006	0.997	0.076	0.010	0.997
Dementia Annual Review	1	0.845	0.957	0.917	0.888	0.957
Memory Loss Codes	0.622	1	0.593	0.456	0.964	0.593
MMSE	0.344	0.187	0.729	0.199	0.157	0.729
Referral To Memory Assessment	0.312	0.049	1	0.143	0.033	1
Referral To Psych/Geri/Neuro	0.345	0.292	0.509	0.248	0.210	0.509
Keyword Dementia	-	-	-	0.494	0.975	0.473
Keyword Memory	-	-	-	0.251	1	0.422
Keyword Confusion	-	-	-	0.238	0.444	0.363
Keyword Behaviour	-	-	-	0.037	0.091	0.520
Keyword Cognition	-	-	-	0.031	0.078	0.515
Keyword Family	-	-	-	0.158	0.079	0.113
Keyword MMSE	-	-	-	0.019	0.119	0.515
Keyword Forget	-	-	-	0.051	0.087	0.439
Keyword Third party consultation	-	-	-	1	0.200	0.544

Discussion

We produced a set of binary coded variables that could be combined in a statistical model and which could accurately classify patients who went on to have a dementia diagnosis formally coded. This model could be take forward to the clinic to identify "known but unlabelled" patients with dementia in primary care, thus improving record keeping, prevalence estimates, and ongoing care for these patients. We

For peer review only - http://bmjopen.bmj.com/site/about/guidelines.xhtml

also investigated whether adding in information from the clinic notes and letters to the model, in the form of a simple keyword extraction, could add further value to the approach. The addition of free text keywords improved the discrimination of the model to an excellent AUC of 0.94, suggesting this model would perform well in the clinic. The improved performance with the addition of free text keywords was achieved because of increased sensitivity of the model, with no loss of specificity, as has been shown in a range of other case-identification studies incorporating free text.[43] This study provides evidence that hidden data in these free text fields can substantially improve automated detection of cases, adding weight to arguments that free text data are valuable for research, if acceptable privacy preserving methods for data-sharing (such as automated information extraction behind a clinical firewall) can be agreed upon.[44] We tried three different machine learning approaches which are suitable for binary data and found all performed similarly, although they weighted the various features differently in their classifications. This is likely to be due to the different construction of the algorithms within each method, for example, random forest algorithms are thought to weight most highly the commonest features in the dataset, rather than features which are rarer but differ more substantially between the two groups. Future work could look at whether there is added value in combining the three approaches, as ensemble methods can sometimes outperform any of the single approaches alone, especially where the different methods are clearly prioritising different features to achieve their performance. However, most have been trialled on multi-class rather than binary classification.[45]

Our study is a novel contribution to the literature. Studies have previously aimed to detect dementia that was unknown to primary care practitioners, by manual [46-48] and automated methods,[28] using data from primary care records. Additionally, a recent study by Aldus et al. [14] has explored rates and predictors of patients with dementia going undetected. However, to the authors' knowledge, no prediction or early detection approaches have made the distinction between patients with dementia unknown to the GP and those known to the GP but nevertheless unrecorded with a formal diagnostic code. The study by Aldus et al. gives the first indication of how many of the "undiagnosed" cohort may be "known but unlabelled", suggesting that both unknown and known but unlabelled groups decline over time, but that of patients with no diagnosis, around 66-71% were not known to have symptoms, while 29-34% had memory concerns or a referral noted in their patient record.[14] These figures were based on small numbers and it would be worth investigating this further to estimate the proportions of each group. Our team has also used social science methods, interviewing and surveying GPs and patients to understand what is happening in this apparent "diagnosis gap" for dementia in UK primary

For peer review only - http://bmjopen.bmj.com/site/about/guidelines.xhtml

care. We suggest that this dual "data science and social science" approach to understanding and utilising data in patient records for clinical decision or diagnostic support is valuable, as it exposes the underlying human decision-making and behaviour which influences how the data are created. These insights can be drawn on to improve clinical prediction models and make them more relevant for key stakeholders, which is likely to improve implementation. A further avenue for exploration with GPs, and other stakeholders such as service commissioners, is whether the results produced by this approach would be of value for either improving individual patient care, or for service and resource planning, or commissioning, due to more accurate prevalence rates in patient data.

Further work should also examine the ethics and desirability of such methods in practice. Previous studies have highlighted a range of potential ethical issues which need further exploration. Firstly, according to the principles of ethnical screening by Wilson and Jungner [49] any kind of screening programme is only ethical if there is an accepted treatment for patients with recognised disease. Weatherby and Agius argue that given no treatments that modify disease trajectory for dementia are available, and given the possibility of false positive results, population screening for dementia is ethically unjustifiable, although a stepwise process that becomes increasingly targeted might be appropriate.[50] A stepwise approach to detection may be especially important given the low PPVs found when assessing our model against a population prevalence. Ienca et al. have also argued that use of big data for early detection may lead to an inflation of the false positive risk, presumably because of algorithms being applied indiscriminately on whole populations.[51] They also draw attention to the need for clinicians or researchers to be cautious about the privacy and disclosure issues of personal data about dementia, as this would be highly sensitive information.[51] Other authors have discussed the ethical implications of genetic testing for Alzheimer risk in early detection or predictive models,[50, 52] as well as needing to maintain the "right not to know" for patients.[53] Drawing on these debates and other literature, Thyrian et al. argue that early recognition is wanted by a majority of patients if offered in primary care, that GPs are also largely in favour,[54] and that evidence-based case finding algorithms may be ethically acceptable in primary care if tailored to both diagnostic procedures and, importantly, a post-diagnostic support package.[55]

Strength and limitations

This study used a large sample of English patients who were all over the age of 65, and patients registered in CPRD are thought to be representative of patients in England as a whole. However, to

For peer review only - http://bmjopen.bmj.com/site/about/guidelines.xhtml

ensure that this model is robust to different patient groups, it should now be evaluated in a new patient sample, perhaps in one which is produced from a different general practice computer system (CPRD Gold data is harvested from VISION software). A further strength is that we specified a very simple model which dichotomised the presence of an indicator from a code list, this means the model can be readily operationalised in any GP record system and should be robust to number and order of code accrual in the patient record.

However, there are important limitations in our study design which could be assessed or addressed in future work. Notably, data collection was limited to cases identified before the end of 2012, and therefore it is unclear whether the same model would apply in current times, as the many government initiatives which have been implemented since our study's data collection may have changed clinical practice and thus recording. We plan future work to assess this, and to strengthen the analysis by using a cohort design and linked data from hospital records and death certificate data. We used a matched case-control design, in which the contribution of age and sex to the model was controlled for in the matching, and in which only one control for each dementia case was used. Using more controls per case would have increased statistical power and reduced the risk that controls were in fact undiagnosed dementia cases. We also chose not to clean up our control group for evidence of dementia medication prescribing as has been done in other studies [56], because we felt that doing so would have artificially inflated the performance of our models. We estimate the prevalence of dementia in the control group would be under 3.5% given the prevalence and rate of unidentified dementia during the study period (50% diagnoses assumed to be missing). We also chose not to use MMSE score, as we aimed to create simple models with binary predictors, and because the majority of MMSE scores were missing from structured data. A future cohort study where the prevalence is lower, where age can be added as a predictor, where possible false negatives can be removed from the control group, and which could explore more complex predictors such as MMSE score, will be a robust assessment of this model's replicability.

A further limitation is that our model was trained on cases who all received a dementia diagnosis code at the end of the study period. There are likely to be a number of dementia cases who never receive a dementia code, and it remains a possibility that these patients differ in systematic ways from cases who do go on to receive a code. The ability of our model to detect patients who never receive a code is therefore unknown. An evaluation in a GP practice, where the predictions of the model are assessed and validated by the practising GP, would give us further information about its performance in real-life clinic

For peer review only - http://bmjopen.bmj.com/site/about/guidelines.xhtml

settings. Finally, we used a very simple keyword extraction to access the keyword data, and did not incorporate any natural language processing to identify negation, hedging, subject or timing of the words we identified. We may therefore have over-sampled instances of the clinical concepts being recorded about the patients, however this would have affected both cases and controls equally and is most likely to have resulted in a bias towards the null. It was important for our study design that we chose simple feature extraction methods which could potentially be recreated in a busy clinic.

Implications and future steps

Our model shows good accuracy in identifying dementia patients who have been detected as likely to have dementia but who currently have no diagnostic code for the condition. As outlined above, two further validation studies are now indicated; first, a replication in a cohort study using similar primary care patient data and second, a proof of concept trial in a local GP practice or primary care network. We hope if these two further pieces of work indicate the same excellent discrimination between patients with dementia and those without, and following steps to determine the threshold resulting in high specificity and sensitivity, this model could be adopted by GPs and CCGs to improve the quality of their dementia registers and to aid service planning.

These findings also provide a potential way for EHR researchers to extend their dementia case identification when using primary care data for dementia research and potentially to improve research quality. Emerging evidence on the rates of undetected dementia [14] as well as novel methods for identifying probable but unlabelled cases such as those reported here, mean that researchers could run sensitivity analyses on a range of more and less sensitive dementia definitions, rather than rely on diagnosis codes alone. We suggest using memory loss symptoms, Alzheimer specific prescriptions, and dementia annual review codes to extend the case definition of dementia in EHR research. MMSE scores, where recorded, could be explored as an additional variable of particular value. As random forest and logistic regression models performed very similarly in this and other studies [29], we would suggest either analytical approach could be valuable for classification tasks in dementia EHR research.

Conclusions

We have shown that a simple model made up of indications of dementia symptoms, screening tests, referral and follow up care in coded and free text data can identify primary care patients who have recognised dementia but no diagnostic code for it in their record. This model can be taken forward for

For peer review only - http://bmjopen.bmj.com/site/about/guidelines.xhtml

further validation, and used by GPs and commissioners to improve the quality of data on patients with dementia and as a result, improve dementia care and services.

Supplementary Materials

Appendix 1: Dementia Code List

Appendix 2: Features Code Lists

Appendix 3: List of Keywords

Appendix 4: RECORD Checklist

Acknowledgements

This work uses data provided by patients and collected by the NHS as part of their care and support. #datasaveslives.

Funder

This project was funded by a grant from the Wellcome Trust ref. 202133/Z/16/Z. The funder had no role in study design, data collection and analysis, decision to publish, or preparation of the manuscript.

Ethics approval and consent to participate

This study was approved by the Independent Scientific Advisory Committee at the Medicines and Healthcare Products Regulatory Authority, UK, protocol number 15_111_RA. Following approval, administrative permissions to access and use the electronic patient records were granted by Clinical Practice Research Datalink (CPRD.com).

Consent for publication

For EHR data: Not applicable

Patient and Public Involvement

Prior to applying for funding a focus group was conducted with people with dementia and their carers to guide the development of the research question. Patients were not involved in the design of the study or the acquisition of data. Results have been disseminated through a series of public presentations to patient interest groups, through a study website (www.bsms.ac.uk/astrodem) and through social media.

For peer review only

Availability of data and materials

The data that support the findings of this study are available from Clinical Practice Research Datalink (CPRD; www.cprd.com) but restrictions apply to the availability of these data, which were used under license for the current study, and so are not publicly available. For re-using these data, an application must be made directly to CPRD.

Authors' contributions

EF, SO and JC conceived and directed the study. EF drew up code lists and PR checked these. PR managed the data. JS conducted the analyses. PR and SO gave data analysis advice. SB and JC gave clinical advice. EF and JS wrote the manuscript. All authors provided critical feedback on the manuscript and approved the final version.

Competing interests

The authors declare that they have no competing interests.

References

1. Prince M, Jackson J, eds. World Alzheimer Report 2009. London: Alzheimer's Disease International; 2009. [Available from: https://www.alz.co.uk/research/files/WorldAlzheimerReport.pdf]
2. Prince M, Knapp M, Guerchet M, McCrone P, Prina M, Comas-Herrera A, et al. Dementia UK Update London, UK; 2014. [Available from: https://kclpure.kcl.ac.uk/portal/files/35828472/P326_AS_Dementia_Report_WEB2.pdf]
3. Department of Health. Living well with dementia: A National Dementia Strategy (2009) [Available from: https://www.gov.uk/government/uploads/system/uploads/attachment_data/file/168220/dh_094051.pdf].
4. Department of Health. Prime Minister's challenge on dementia: Delivering major improvements in dementia care and research by 2015 (2012) [Available from: https://www.gov.uk/government/uploads/system/uploads/attachment_data/file/215101/dh_133176.pdf].
5. Department of Health. Prime Minister's Challenge on Dementia 2020 (2020) [Available from: https://assets.publishing.service.gov.uk/government/uploads/system/uploads/attachment_data/file/414344/pm-dementia2020.pdf].
6. Prince M, Bryce R, Ferri C. World Alzheimer Report 2011: The benefits of early diagnosis and intervention: Alzheimer's Disease International; 2011. [Available from: https://www.alz.co.uk/research/world-report-2011]
7. Leifer BP. Early diagnosis of Alzheimer's disease: clinical and economic benefits. *J Am Geriatr Soc*. 2003;51(5s2):S281-S8.
8. Banerjee S. Living well with dementia—development of the national dementia strategy for England. *Int J Geriatr Psychiatry*. 2010;25(9):917-22.
9. NHS England. Enhanced Service Specification: Dementia Identification Scheme 2014 [Available from: http://www.england.nhs.uk/wp-content/uploads/2014/10/dementia-ident-schm-fin.pdf].

For peer review only - http://bmjopen.bmj.com/site/about/guidelines.xhtml

10. NHS England. Dementia diagnosis rate workbooks 2017 [Available from: https://www.england.nhs.uk/publication/dementia-diagnosis-rate-workbook/].
11. Matthews FE, Stephan BC, Robinson L, Jagger C, Barnes LE, Arthur A, et al. A two decade dementia incidence comparison from the Cognitive Function and Ageing Studies I and II. *Nat Commun*. 2016;7:11398.
12. Donegan K, Fox N, Black N, Livingston G, Banerjee S, Burns A. Trends in diagnosis and treatment for people with dementia in the UK from 2005 to 2015: a longitudinal retrospective cohort study. *Lancet Public Health*. 2017;2(3):e149-e56.
13. Low L-F, McGrath M, Swaffer K, Brodaty H. Communicating a diagnosis of dementia: A systematic mixed studies review of attitudes and practices of health practitioners. *Dementia*. 2019;18(7-8):2856-905.
14. Aldus CF, Arthur A, Dennington-Price A, Millac P, Richmond P, Dening T, et al. Undiagnosed dementia in primary care: a record linkage study. *Health Services and Delivery Research*. 2020;8(20).
15. Lang L, Clifford A, Wei L, Zhang D, Leung D, Augustine G, et al. Prevalence and determinants of undetected dementia in the community: a systematic literature review and a meta-analysis. *BMJ Open*. 2017;7(2):e011146.
16. Moore V, Cahill S. Diagnosis and disclosure of dementia—A comparative qualitative study of Irish and Swedish general practitioners. *Aging & mental health*. 2013;17(1):77-84.
17. Lahjibi-Paulet H, Dauffy A, Minard A, Gaxatte C, Saint-Jean O, Somme D. Attitudes toward Alzheimer's disease: a qualitative study of the role played by social representation on a convenient sample of French general practitioners. *Aging Clin Exp Res*. 2012;24(4):384-90.
18. Dhedhi SA, Swinglehurst D, Russell J. 'Timely'diagnosis of dementia: what does it mean? A narrative analysis of GPs' accounts. *BMJ Open*. 2014;4(3):e004439.
19. Hansen EC, Hughes C, Routley G, Robinson AL. General practitioners' experiences and understandings of diagnosing dementia: factors impacting on early diagnosis. *Soc Sci Med*. 2008;67(11):1776-83.
20. Pentzek M, Wollny A, Wiese B, Jessen F, Haller F, Maier W, et al. Apart from nihilism and stigma: what influences general practitioners' accuracy in identifying incident dementia? *The American journal of geriatric psychiatry*. 2009;17(11):965-75.
21. Brayne C, Kelly S. Against the stream: early diagnosis of dementia, is it so desirable? *BJPsych Bulletin*. 2019;43(3):123-5.
22. Pimlott NJG, Persaud M, Drummond N, Cohen CA, Silvius JL, Seigel K, et al. Family physicians and dementia in Canada Part 2. Understanding the challenges of dementia care. *Can Fam Physician*. 2009;55(5):508-U64.
23. Boise L, Camicioli R, Morgan DL, Rose JH, Congleton L. Diagnosing dementia: Perspectives of primary care physicians. *Gerontologist*. 1999;39(4):457-64.
24. Iliffe S, Manthorpe J, Eden A. Sooner or later? Issues in the early diagnosis of dementia in general practice: a qualitative study. *Fam Pract*. 2003;20(4):376-81.
25. Stephan BC, Tang E, Muniz-Terrera G. Composite risk scores for predicting dementia. *Curr Opin Psychiatry*. 2016;29(2):174-80.
26. The PHG Foundation. Dementia Risk Prediction Models: What do policy makers need to know? Cambridge, UK: The University of Cambridge; 2019 [Available from: https://www.phgfoundation.org/documents/dementia-risk-prediction-models.pdf].
27. Walters K, Hardoon S, Petersen I, Iliffe S, Omar RZ, Nazareth I, et al. Predicting dementia risk in primary care: development and validation of the Dementia Risk Score using routinely collected data. *BMC Med*. 2016;14(1):1-12.

For peer review only - http://bmjopen.bmj.com/site/about/guidelines.xhtml

28. Jammeh EA, Camille BC, Stephen WP, Escudero J, Anastasiou A, Zhao P, et al. Machine-learning based identification of undiagnosed dementia in primary care: a feasibility study. *BJGP Open*. 2018;2(2):bjgpopen18X101589.
29. Ford E, Rooney P, Oliver S, Hoile R, Hurley P, Banerjee S, et al. Identifying undetected dementia in UK primary care patients: a retrospective case-control study comparing machine-learning and standard epidemiological approaches. *BMC Med Inform Decis Mak*. 2019;19(1):248.
30. Ford E, Starlinger J, Rooney P, Oliver S, Banerjee S, van Marwijk H, et al. Could dementia be detected from UK primary care patients' records by simple automated methods earlier than by the treating physician? A retrospective case-control study. *Wellcome Open Res*. 2020;5(120):120.
31. Russell P, Banerjee S, Watt J, Adleman R, Agoe B, Burnie N, et al. Improving the identification of people with dementia in primary care: evaluation of the impact of primary care dementia coding guidance on identified prevalence. *BMJ Open*. 2013;3(12).
32. Benchimol EI, Smeeth L, Guttmann A, Harron K, Moher D, Petersen I, et al. The REporting of studies Conducted using Observational Routinely-collected health Data (RECORD) Statement. *PLOS Medicine*. 2015;12(10):e1001885.
33. Wolf A, Dedman D, Campbell J, Booth H, Lunn D, Chapman J, et al. Data resource profile: Clinical Practice Research Datalink (CPRD) Aurum. *Int J Epidemiol*. 2019;48(6):1740-g.
34. Herrett E, Gallagher AM, Bhaskaran K, Forbes H, Mathur R, van Staa T, et al. Data resource profile: clinical practice research datalink (CPRD). *Int J Epidemiol*. 2015;44(3):827-36.
35. Chisholm J. The Read clinical classification. *Brit Med J*. 1990;300:1092.
36. Booth N. What are the Read Codes? *Health Libr Rev*. 1994;11(3):177-82.
37. Rait G, Walters K, Bottomley C, Petersen I, Iliffe S, Nazareth I. Survival of people with clinical diagnosis of dementia in primary care: cohort study. *Brit Med J*. 2010;341:c3584.
38. Wiest MM, Lee KJ, Carlin JB. Statistics for clinicians: An introduction to logistic regression. *Journal of paediatrics and child health*. 2015;51(7):670-3.
39. Tibshirani R. Regression shrinkage and selection via the LASSO. *Journal of the Royal Statistical Society Series B (Methodological)*. 1996:267-88.
40. Alam MZ, Rahman MS, Rahman MS. A Random Forest based predictor for medical data classification using feature ranking. *Informatics in Medicine Unlocked*. 2019;15:100180.
41. Stone JV. Bayes' rule: a tutorial introduction to Bayesian analysis: Sebtel Press; 2013.
42. Langarizadeh M, Moghbeli F. Applying Naive Bayesian Networks to Disease Prediction: a Systematic Review. *Acta Inform Med*. 2016;24(5):364-9.
43. Ford E, Carroll JA, Smith H, Scott D, Cassell J. Extracting information from the text of electronic medical records to improve case detection: a systematic review. *J Am Med Inform Assoc*. 2016;doi: 10.1093/jamia/ocv180.
44. Jones KH, Ford EM, Lea N, Griffiths LJ, Hassan L, Heys S, et al. Toward the Development of Data Governance Standards for Using Clinical Free-Text Data in Health Research: Position Paper. *J Med Internet Res*. 2020;22(6):e16760.
45. Galar M, Fernández A, Barrenechea E, Bustince H, Herrera F. An overview of ensemble methods for binary classifiers in multi-class problems: Experimental study on one-vs-one and one-vs-all schemes. *Pattern Recognition*. 2011;44(8):1761-76.
46. Banerjee S, Willis R, Matthews D, Contell F, Chan J, Murray J. Improving the quality of care for mild to moderate dementia: an evaluation of the Croydon Memory Service Model. *Int J Geriatr Psychiatry*. 2007;22(8):782-8.
47. Kallumpuram S, Kumar CS, Khan B, Gavins V, Khan A, Iliffe S. Targeted case finding for dementia in primary care: Surrey Downs dementia diagnosis project. *BMJ Open Qual*. 2015;4(1):u209827. w4086.

For peer review only

48. Wray LO, Wade M, Beehler GP, Hershey LA, Vair CL. A Program to Improve Detection of Undiagnosed Dementia in Primary Care and Its Association with Healthcare Utilization. *The American journal of geriatric psychiatry.* 2014;22(11):1282-91.

49. Wilson JMG, Jungner G.. Principles and practice of screening for disease. 1968. World Health Organisation, Geneva, Switzerland.

50. Weatherby TJ, Agius M. Ethical and Organisational considerations in screening for dementia. *Psychiatria Danubina*. 2018;30(Suppl 7):463-8.

51. Ienca M, Vayena E, Blasimme A. Big Data and Dementia: Charting the Route Ahead for Research, Ethics, and Policy. *Frontiers in Medicine*. 2018;5(13).

52. Arribas-Ayllon M. The ethics of disclosing genetic diagnosis for Alzheimers disease: Do we need a new paradigm? *British medical bulletin*. 2011;100:7-21.

53. Schweda M, Kögel A, Bartels C, Wiltfang J, Schneider A, Schicktanz S. Prediction and early detection of Alzheimer's dementia: professional disclosure practices and ethical attitudes. *Journal of Alzheimer's Disease*. 2018;62(1):145-55.

54. Thyrian JR, Eichler T, Pooch A, Albuerne K, Dreier A, Michalowsky B, et al. Systematic, early identification of dementia and dementia care management are highly appreciated by general physicians in primary care - results within a cluster-randomized-controlled trial (DelpHi). *J Multidiscip Healthc*. 2016;9:183-90.

55. Thyrian JR, Hoffmann W, Eichler T. Early Recognition of Dementia in Primary Care-Current Issues and Concepts. *Current Alzheimer Research*. 2018;15(1):2-4.

56. Ponjoan A, Garre-Olmo J, Blanch J, Fages E, Alves-Cabratosa L, Martí-Lluch R, et al. Epidemiology of dementia: prevalence and incidence estimates using validated electronic health records from primary care. *Clinical epidemiology*. 2019;11:217-28.

For peer review only

For peer review only - http://bmjopen.bmj.com/site/about/guidelines.xhtml

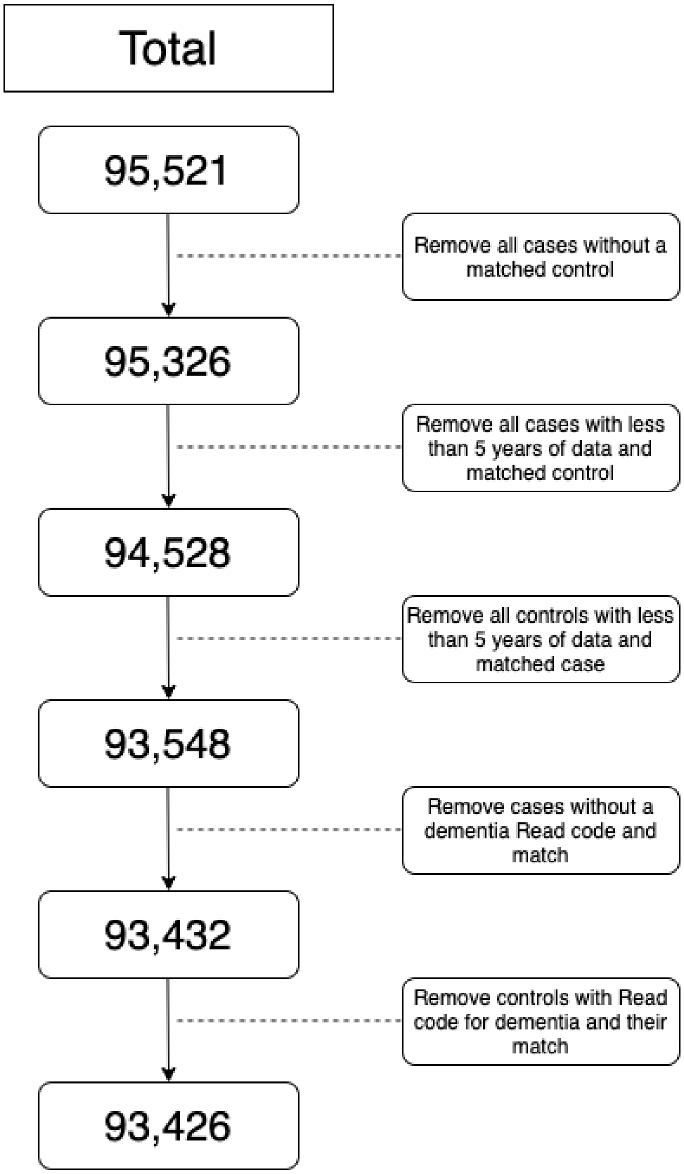

Figure 1: Final Participant Selection.

108x182mm (96 x 96 DPI)

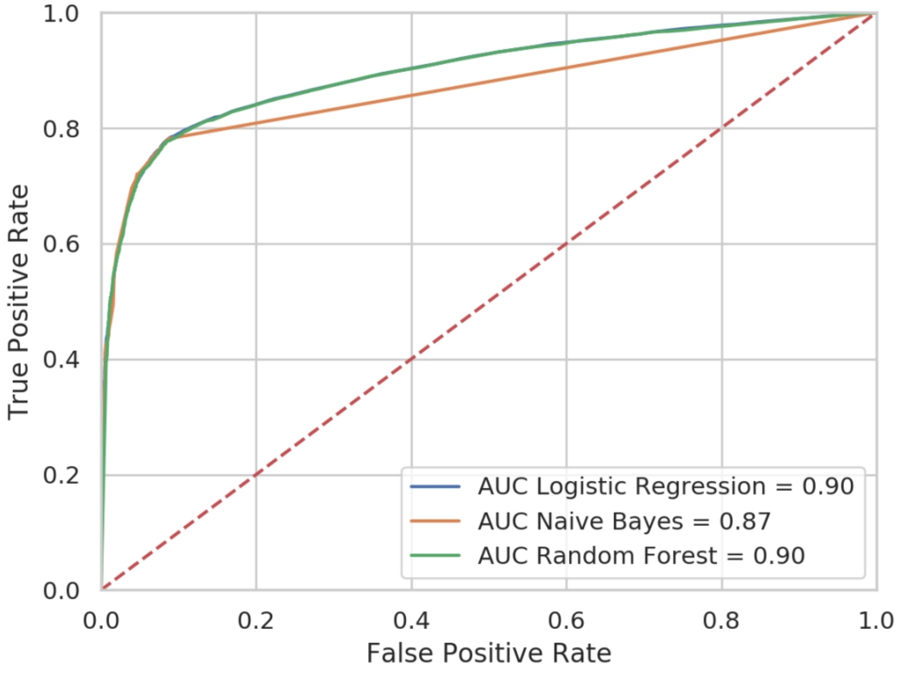

Figure 2: ROC showing discrimination between dementia cases and controls by three machine learning models with codes only.

338x233mm (96 x 96 DPI)

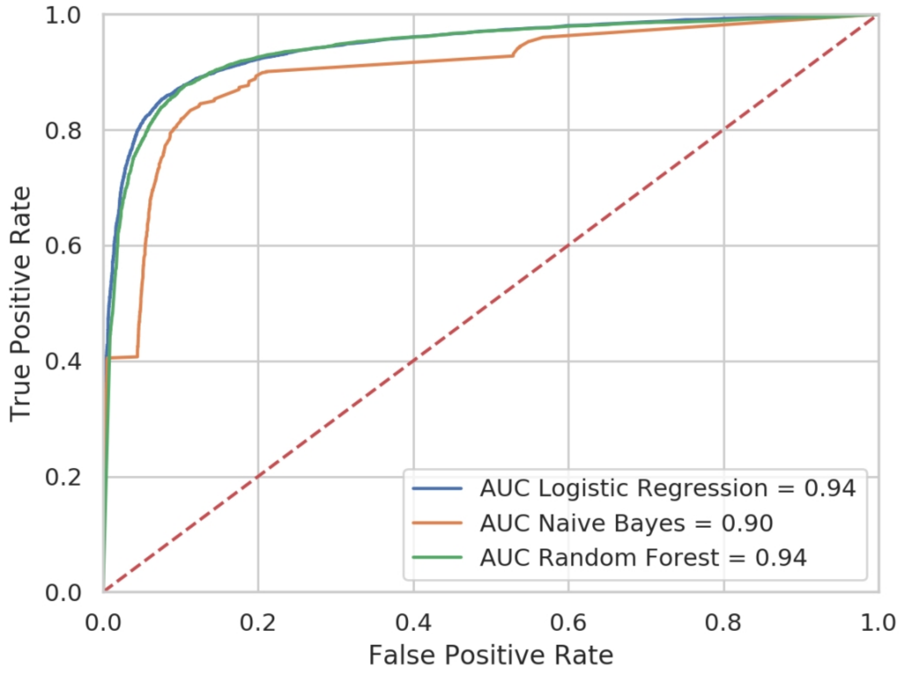

Figure 3: ROC showing discrimination between dementia cases and controls by three machine learning models with both codes and keywords.

338x233mm (96 x 96 DPI)

ANNEX 1 - DEMENTIA DIAGNOSTIC READ CODES

Read Description	Read Code	Medical Code
**Alzheimers codes**		
Dementia in Alzheimer's disease	Eu00	7664
Dementia in Alzheimer's disease with early onset	Eu000	49263
Dementia in Alzheimer's disease with late onset	Eu001	38678
Dementia in Alzheimer's disease, atypical or mixed type	Eu002	30706
Dementia in Alzheimer's disease unspecified	Eu00z	29386
Alzheimer's disease	F110	1917
Alzheimer's disease with early onset	F1100	16797
Alzheimer's disease with late onset	F1101	32057
[X]Presenile dementia,Alzheimer's type	Eu00011	25704
[X]Primary degen dementia, Alzheimer's type, presenile or	Eu00012	60059
[X]Alzheimer's disease type 2	Eu00013	61528
[X]Alzheimer's disease type 1	Eu00111	46762
[X]Senile dementia,Alzheimer's type	Eu00112	11379
[X]Primary degen dementia of Alzheimer's type, senile ons	Eu00113	43346
[X]Alzheimer's dementia unspec	Eu00z11	8195
	15	
**Vascular Dementia Codes**		
Vascular dementia	Eu01	6578
Arteriosclerotic dementia	E004	19477
Multi-infarct dementia	Eu011	8634
Subcortical vascular dementia	Eu012	8934
Mixed cortical and subcortical vascular dementia	Eu013	31016
Other vascular dementia	Eu01y	55313
Vascular dementia unspecified	Eu01z	19393
Uncomplicated arteriosclerotic dementia	Eu0040	43089
Arteriosclerotic dementia with delirium	Eu0041	56912
Arteriosclerotic dementia with paranoia	Eu0042	55467
Arteriosclerotic dementia with depression	Eu0043	43292
Arteriosclerotic dementia NOS	Eu004z	42279
[X]Vascular dementia of acute onset	Eu01000	46488
[X]Predominantly cortical dementia	Eu01111	55838
[X] Multi-infarct dementia	Eu01100	11175
	15	
**Dementia in other diseases**		
[X] Dementia in other diseases classified elsewhere	Eu02y00	64267
Dementia in Pick's disease	Eu020	28402
Circumscribed brain atrophy including Pick's disease	F111	?
Dementia in Creutzfeldt-Jackob disease	Eu021	54106
Dementia in Huntingdon's disease	Eu022	37014
Dementia in Parkinson's disease	Eu023	9509
Dementia in HIV disease	Eu024	4485
Dementia in conditions EC	E041.00	25386
Alcoholic dementia NOS	Eu10711	27342
[X] Alcoholic dementia NOS	Eu10711	26323
Other alcoholic dementia	E012	54505
Drug-induced dementia	E02y100	62132
Lewy body disease	F116	7572
Lewy body dementia	Eu025	26270
	14	
**Dementia not otherwise classified**		
Unspecified dementia	Eu02z	4693
Senile/Presenile dementia	Eu00..12	1350

For peer review only

Presenile dementia	E001	15165
Uncomplicated presenile dementia	E0010	46202
Presenile dementia with delirium	E0011	49513
Presenile dementia with paranoia	E0012	30032
Presenile dementia with depression	E0013	27677
Presenile dementia NOS	E0014	38438
[X] Presenile dementia NOS	Eu02z11	48501
Uncomplicated senile dementia	E000	7323
Senile dementia	E00..11	1916
Senile dementia with depressive or paranoid features	E002	44674
Senile dementia with paranoia	E0020	18386
Senile dementia with depression	E0021	21887
Senile dementia with depressive or paranoid features NOS	E002z	41089
Delirium superimposed on dementia	Eu041	53446
Senile dementia with delirium	E003	37015
Senile degeneration of the brain, not elsewhere classified	F112	29512
	18	
**Total N codes**	**62**	

For peer review only

Cognitive Decline

Read term	Read code	Medcode
Cognitive decline	28E..00	7674
GDS level 2 - very mild cognitive decline	3AE1.00	65856
GDS level 3 - mild cognitive decline	3AE2.00	60263
GDS level 4 - moderate cognitive decline	3AE3.00	60726
GDS level 5 - moderately severe cognitive decline	3AE4.00	70057
GDS level 6 - severe cognitive decline	3AE5.00	94717
GDS level 7 - very severe cognitive decline	3AE6.00	72520
[X]Mild cognitive disorder	Eu05700	11936
[X]Oth & unspec symptom/sign involv cognit funct/awareness	Ryu5100	52939
Health of the Nation Outcome Scale item 4 - cognitive probl	ZRLfE00	35194
Impaired cognition	Z7C1.00	10822
Cognitive function observations	Z7C..00	18906

Cognitive Screening Tests

Read term	Read code	Medcode
Cognitive assessment	311B.00	95853
GPCOG - general practitioner assessment of cognition	38Dv.00	101074
GPCOG (GP assessment of cognition) patient examination	38Dv000	103076
GPCOG (GP assessment of cognition) informant interview	38Dv100	101710
Six item cognitive impairment test	3AD3.00	35305
Cognitions questionnaire	ZR3a.00	36650
Cognitive failures questionnaire	ZR3b.00	66080
Microcog - assessment of cognitive function	ZRa2.00	22257
Neurobehavioural cognitive status examination	ZRaY.00	42059
Rancho scale - levels of cognitive functioning	ZRd..00	92756
Recognition memory test	ZRh6.00	96881

For peer review only

RMT - Recognition memory test	ZRh6.11	93236
Smith cognitive questionnaire	ZRkL.00	96828
Health of the Nation Outcome Scale item 4 - cognitive probl	ZRLfE00	35194
Kendrick cognitive tests for the elderly	ZRV9.11	63604
Lowenstein OT cognitive assessment	ZRVa.00	26264
LOTCA - Lowenstein OT cognitive assessment	ZRVa.11	98256
Measurement of cognitive linguistic ability	ZRVt.00	62114
MCLA - Measurement of cognitive linguistic ability	ZRVt.11	93194

Dementia Annual Review

Read term	Read code	Medcode
Dementia annual review	6AB..00	12710

Memory Loss Codes

Read term	Read code	Medcode
Memory loss - amnesia	1B1A.00	1993
Poor auditory sequential memory	1B1a.00	40821
Amnesia symptom	1B1A.11	3639
Memory loss symptom	1B1A.12	5777
Memory disturbance	1B1A.13	2908
Temporary loss of memory	1B1A000	27788
Short-term memory loss	1B1A100	103453
Transient global amnesia	1B1S.00	28278
Poor visual sequential memory	1B1Y.00	39507
Disturbance of memory for order of events	1S21.00	67163
Forgetful	28G..00	7742
Memory: own age not known	3A10.00	52947
Memory: present time not known	3A20.00	53146
Memory: present place not knwn	3A30.00	53014
Memory: present year not known	3A40.00	52948
Memory: own DOB not known	3A50.00	53125
Memory: present month not knwn	3A60.00	52825
Memory: important event not kn	3A70.00	52800

For peer review only

Memory: import.person not knwn	3A80.00	52801
Memory: count down unsuccess.	3A91.00	52805
Memory: address recall unsucc.	3AA1.00	53016
Patient forgets to take medication	8BIk.00	68703
Non-alcoholic amnestic syndrome	E040.00	27414
Hysterical amnesia	E201700	4269
Mild memory disturbance	E2A1000	6387
Organic memory impairment	E2A1100	6061
[X]Organic amnesic synd not induced alc/oth psychoact subs	Eu03.00	37072
[X]Dissociative amnesia	Eu44000	40994
Transient global amnesia	G655.00	6489
[D]Amnesia (retrograde)	R00z000	4284
[D]Memory deficit	R00z011	7711
[D]Anterograde amnesia	R00z500	54882
[X]Other amnesia	Ryu5000	70677
Memory disturbance (& amnesia (& symptom))	Z7CE400	51379
Memory loss symptom	Z7CE412	67838
Memory loss - amnesia	Z7CE413	103375
Memory disturbance	Z7CE414	105538
Loss of memory	Z7CE415	102880
Forgetful	Z7CE500	24952
Amnesia	Z7CE600	28406
Memory loss	Z7CE611	10123
Memory gone	Z7CE612	68230
Dysmnesia	Z7CE613	66001
Memory loss - amnesia	Z7CE614	12805
Loss of memory	Z7CE615	19297
LOM - Loss of memory	Z7CE616	12277
Transient global amnesia	Z7CE700	18996
TGA - Transient global amnesia	Z7CE711	19004
Anterograde amnesia	Z7CE800	36767
Antegrade amnesia	Z7CE811	43905
Retrograde amnesia	Z7CE900	35538
RA - Retrograde amnesia	Z7CE911	70703
Impairment of registration	Z7CEA00	49897
Impairment of working memory	Z7CEA11	32367
Impairment of immediate recall	Z7CEA12	59844
Impairment of primary memory	Z7CEA13	65696
Amnesia for remote events	Z7CEB00	66144

For peer review only

Poor memory for remote events	Z7CEB12	37191
Amnesia for recent events	Z7CEC00	61816
Loss of memory for recent events	Z7CEC11	9786
No memory for recent events	Z7CEC12	67802
Amnesia for day to day facts	Z7CED00	68392
Amnesia for important personal information	Z7CEE00	57812
Temporary loss of memory	Z7CEF00	67998
Transient memory loss	Z7CEG00	47882
Memory impairment	Z7CEH00	10514
Memory dysfunction	Z7CEH11	39915
Memory deficit	Z7CEH12	50418
Bad memory	Z7CEH13	26434
Memory problem	Z7CEH14	12057
Poor memory	Z7CEH15	12583
Mixes past with present	Z7CEI00	103922
Memory lapses	Z7CEJ00	19073
Minor memory lapses	Z7CEK00	53507
Mild memory disturbance	Z7CEL00	51724
Distortion of memory	Z7CEM00	51739
Invents experiences to compensate for loss of memory	Z7CEN11	64892
Poor short-term memory	Z7CF800	11410
Short-term memory loss	Z7CF811	10571
Unable to recall random address at five minutes	Z7CFA00	106055
Unable to recall five digit number at five minutes	Z7CFC00	104514
Unable to remember name of current prime minister	Z7CFe00	93856
Forgets what was going to do	Z7CFF00	59515
Forgets what was going to say	Z7CFG00	60600
Cannot remember names of intimates	Z7CFg00	105793
Forgets recent activities	Z7CFH00	41366
Cannot remember birth dates of children	Z7CFh00	54430
Forgets what has just done	Z7CFI00	40091
Cannot remember wedding anniversary	Z7CFi00	62749
Forgets what has just said	Z7CFJ00	93319
Forgets what has just read	Z7CFK00	70895
Forgets what has just seen	Z7CFL00	103479
Forgets what has just heard	Z7CFM00	47994
Poor long-term memory	Z7CFO00	53978
Long-term memory loss	Z7CFO11	47581

For peer review only

Unable to remember motor skills	Z7CFq00	46314
Unable to remember own date of birth	Z7CFQ00	63942
Unable to remember own age	Z7CFS00	32599
Unable to remember new motor skills	Z7CFs00	57750
Cannot remember own age	Z7CFS11	69118
Unable to remember day of the week	Z7CFU00	63849
Memory aided by use of diary	Z7CFw00	59830
Unable to remember today's date	Z7CFW00	66684
Memory aided by use of labels	Z7CFx00	98287
Memory aided by use of lists	Z7CFz00	67951
Delayed verbal memory	Z7CGP00	98798

MMSE

**Read term**	**Read code**	**Medcode**
Mini-mental state examination	388m.00	82481
MMSE score	388m.11	83484
Mini mental state score	388V.00	11862
Mini-mental state examination	ZRaA.00	10503
MMSE - Mini-mental state examination	ZRaA.11	10493
Modified mini-mental state 3MS examination	ZRaA100	59387
Modified mini-mental state examination	ZRaA200	36637

Referral to memory assessment services

**Read term**	**Read code**	**Medcode**
Referral to memory clinic	8HTY.00	82481
Seen in memory clinic	9Nk1.00	83484

Referral to Psychiatrist, Neurologist or Geriatrician

**Read term**	**Read code**	**Medcode**
Neurological referral	8H46.00	2964
Geriatric referral	8H47.00	1511
Referral to community geriatrician	8H47000	105235
Psychiatric referral	8H49.00	2189
Referral to psychogeriatrician	8H4D.00	5338
Referral to neurologist	8H4h.00	95859

For peer review only

Referral to neurology special interest general practitioner	8H4Y.00	55362
Psychiatric self-referral	8HJ3.00	27640
Geriatric self-referral	8HJ4.00	68924
Neurology self-referral	8HJE.00	40890
Urgent referral to psychiatrist	8HIB.00	95936
Private referral to neurologist	8HVL.00	13653
Private referral to geriatrician	8HVM.00	29200
Private referral to psychiatrist	8HVO.00	13677
Private referral to psychogeriatrician	8HVS.00	34532
Referral to care of the elderly physician	ZL5A200	12077
Referral to geriatrician	ZL5A211	11634
Referral to neurologist	ZL5AO00	10448
Referral to psychiatrist	ZL5B.00	10002

Alzheimer's prescription Product Codes (specific to CPRD)

Code	Product Description	Molecule	Dose, format and route
7329	Galantamine 20mg/5ml oral solution sugar free	Galantamine hydrobromide	4mg/1ml, Oral solution, Oral
36976	Rivastigmine 4.6mg/24hours transdermal patches	Rivastigmine	4.6mg/24 Hours, Transdermal Patch, Transdermal
33007	RIVASTIGMINE	Unknown	Unknown
56771	Rivastigmine 3mg capsules (Dr Reddy's Laboratories (UK) Ltd)	Rivastigmine hydrogen tartrate	3mg, Capsule, Oral
11751	Rivastigmine 3mg capsules	Rivastigmine hydrogen tartrate	3mg, Capsule, Oral
53922	Donepezil 10mg orodispersible tablets (Consilient Health Ltd)	Donepezil hydrochloride	10mg, Orodispersible tablet, Oral
10255	Galantamine 8mg modified-release capsules	Galantamine Hydrobromide	8mg, Modified Release Capsules, Oral
38976	Memantine 5mg+10mg+15mg+20mg Tablet	Memantine Hydrochloride	5mg+10mg+15mg+20mg, Tablet, Oral
33008	RIVASTIGMINE	Unknown	Unknown
33009	RIVASTIGMINE	Unknown	Unknown
60723	Rivastigmine 6mg capsules (Waymade Healthcare Plc)	Rivastigmine hydrogen tartrate	6mg, Capsule, Oral
56600	Donepezil 5mg tablets (Zentiva)	Donepezil hydrochloride	5mg, Tablet, Oral
11752	Rivastigmine 4.5mg capsules	Rivastigmine hydrogen tartrate	4.5mg, Capsule, Oral
11635	Galantamine 12mg tablets	Galantamine hydrobromide	12mg, Tablet, Oral
48443	Donepezil 10mg orodispersible tablets	Donepezil hydrochloride	10mg, Orodispersible tablet, Oral

For peer review only

53882	Rivastigmine 2mg/ml oral solution	Rivastigmine hydrogen tartrate	2mg/1ml, Oral solution, Oral
2931	Donepezil 10mg tablets	Donepezil hydrochloride	10mg, Tablet, Oral
6225	Memantine 10mg tablets	Memantine hydrochloride	10mg, Tablet, Oral
63217	Donepezil 5mg tablets (A A H Pharmaceuticals Ltd)	Donepezil hydrochloride	5mg, Tablet, Oral
11654	Galantamine 8mg tablets	Galantamine hydrobromide	8mg, Tablet, Oral
56631	Rivastigmine 13.3mg/24hours transdermal patches	Rivastigmine	13.3mg/24hour, Transdermal patch, Transdermal
39240	Memantine 20mg tablets	Memantine hydrochloride	20mg, Tablet, Oral
63951	Rivastigmine 9.5mg/24hours transdermal patches (Actavis UK Ltd)	Rivastigmine	9.5mg/24hour, Transdermal patch, Transdermal
11827	Rivastigmine 2mg/ml oral solution sugar free	Rivastigmine hydrogen tartrate	2mg/1ml, Oral solution, Oral
35088	Donepezil 10mg orodispersible tablets sugar free	Donepezil Hydrochloride	10mg, Orodispersible Tablet, Oral
60107	Donepezil 5mg tablets (Alliance Healthcare (Distribution) Ltd)	Donepezil Hydrochloride	5mg, tablet, Oral
10187	Galantamine 4mg tablets	Galantamine hydrobromide	4mg, Tablet, Oral
58969	Rivastigmine 4.6mg/24hours transdermal patches (A A H Pharmaceuticals Ltd)	Rivastigmine	4.6mg/24hour, Transdermal patch, Transdermal
59871	Donepezil 10mg/5ml oral suspension	Donepezil hydrochloride	2mg/1ml, Oral suspension, Oral
2930	Donepezil 5mg tablets	Donepezil hydrochloride	5mg, Tablet, Oral
35179	Donepezil 5mg orodispersible tablets sugar free	Donepezil hydrochloride	5mg, Orodispersible tablet, Oral
7361	Galantamine 24mg modified-release capsules	Galantamine hydrobromide	24mg, Modified-release capsule, Oral
4597	Rivastigmine 1.5mg capsules	Rivastigmine hydrogen tartrate	1.5mg, Capsule, Oral
61676	Donepezil 1mg/ml oral solution sugar free	Donepezil hydrochloride	1mg/1ml, Oral solution, Oral
58947	Donepezil 10mg tablets (Accord Healthcare Ltd)	Donepezil hydrochloride	10mg, Tablet, Oral
14309	Galantamine 16mg modified-release capsules	Galantamine Hydrobromide	16mg, Modified Release Capsules, Oral
11837	Memantine 10mg/ml oral solution sugar free	Memantine hydrochloride	10mg/1ml, Oral drops, Oral
58709	Donepezil 10mg tablets (A A H Pharmaceuticals Ltd)	Donepezil hydrochloride	10mg, Tablet, Oral

For peer review only

9786	Rivastigmine 6mg capsules	Rivastigmine hydrogen tartrate	6mg, Capsule, Oral
37132	Rivastigmine 9.5mg/24hours transdermal patches	Rivastigmine	9.5mg/24hour, Transdermal patch, Transdermal
48442	Donepezil 5mg orodispersible tablets	Donepezil hydrochloride	5mg, Orodispersible tablet, Oral -

For peer review only

Keyword	Stem	misspellings	
**Memory**	memory		
	memori*	memroy	
		memeory	
		memmory	
		meory	
		memort	
		memrory	
		memorry	
**cognition**	cognition	cogition	
	cognitive	cogntion	
	cognit*	cngition	
		congtion	
		cogniton	
		congit*	
		cgnit*	
**forgetfulness**	forgetting	foget*	
	forgetful*	forgot*	
	forget*	forgte	
		fforget*	
		froget*	
**confusion**	confusion	confudes	
	confused	condusef	
	confus*	cofnused	
**dementia**	dementia		
	dement*		
	Alzheimer's	alzheimers	
		alzhemer	
		alzhiemer	
		alzhiemers	
		alzhimer	
		alzhimers	
		alzimer	
		alz*	
**behaviour change**	behaviour	behavour	
	behavior	bahaviour	
	behav*	bahavior	
		bahavioe	
		behavoir	
		behaveour	
**family concerned**	family + concerned	**for family substitute:**	**for concerned substitute**
		relative	conserned
		wife	concern*
		husband	worried
		son	
		daughter	
		partner	
		spouse	
		niece	

For peer review only

		nephew
		sister
		brother
		relative
		neighbour
**3rd Party**	third party consultation	third party consulation
		3rd party consultation
		3rd party consulation
**MMSE**	MMSE	
	mini mental state	
	mini-mental state	
	GP Cog	

For peer review only

The RECORD statement – checklist of items, extended from the STROBE statement, that should be reported in observational studies using routinely collected health data.

	Item No.	STROBE items	Location in manuscript where items are reported	RECORD items	Location in manuscript where items are reported
**Title and abstract**					
	1	(a) Indicate the study's design with a commonly used term in the title or the abstract (b) Provide in the abstract an informative and balanced summary of what was done and what was found		RECORD 1.1: The type of data used should be specified in the title or abstract. When possible, the name of the databases used should be included.<br><br>RECORD 1.2: If applicable, the geographic region and timeframe within which the study took place should be reported in the title or abstract.<br><br>RECORD 1.3: If linkage between databases was conducted for the study, this should be clearly stated in the title or abstract.	Title and abstract<br><br>Abstract<br><br>N/A
**Introduction**					
Background rationale	2	Explain the scientific background and rationale for the investigation being reported			4-6
Objectives	3	State specific objectives, including any prespecified hypotheses			6
**Methods**					
Study Design	4	Present key elements of study design early in the paper			6
Setting	5	Describe the setting, locations, and relevant dates, including periods of recruitment, exposure, follow-up, and data collection			6-7

For peer review only

Participants	6	*(a) Cohort study* - Give the eligibility criteria, and the sources and methods of selection of participants. Describe methods of follow-up<br>*Case-control study* - Give the eligibility criteria, and the sources and methods of case ascertainment and control selection. Give the rationale for the choice of cases and controls<br>*Cross-sectional study* - Give the eligibility criteria, and the sources and methods of selection of participants<br><br>*(b) Cohort study* - For matched studies, give matching criteria and number of exposed and unexposed<br>*Case-control study* - For matched studies, give matching criteria and the number of controls per case	7-8<br><br>7-8	RECORD 6.1: The methods of study population selection (such as codes or algorithms used to identify subjects) should be listed in detail. If this is not possible, an explanation should be provided.<br><br>RECORD 6.2: Any validation studies of the codes or algorithms used to select the population should be referenced. If validation was conducted for this study and not published elsewhere, detailed methods and results should be provided.<br><br>RECORD 6.3: If the study involved linkage of databases, consider use of a flow diagram or other graphical display to demonstrate the data linkage process, including the number of individuals with linked data at each stage.	7 (Appendix 1)<br><br>N/A<br><br>N/A
Variables	7	Clearly define all outcomes, exposures, predictors, potential confounders, and effect modifiers. Give diagnostic criteria, if applicable.	8-9	RECORD 7.1: A complete list of codes and algorithms used to classify exposures, outcomes, confounders, and effect modifiers should be provided. If these cannot be reported, an explanation should be provided.	8-9 and Supplementary Materials (Appendix 2 & 3)
Data sources/ measurement	8	For each variable of interest, give sources of data and details of methods of assessment (measurement).<br>Describe comparability of assessment methods if there is more than one group	8-9		

For peer review only

Bias	9	Describe any efforts to address potential sources of bias	7		
Study size	10	Explain how the study size was arrived at	7		
Quantitative variables	11	Explain how quantitative variables were handled in the analyses. If applicable, describe which groupings were chosen, and why	8-9		
Statistical methods	12	(a) Describe all statistical methods, including those used to control for confounding<br>(b) Describe any methods used to examine subgroups and interactions<br>(c) Explain how missing data were addressed<br>(d) *Cohort study* - If applicable, explain how loss to follow-up was addressed<br>*Case-control study* - If applicable, explain how matching of cases and controls was addressed<br>*Cross-sectional study* - If applicable, describe analytical methods taking account of sampling strategy<br>(e) Describe any sensitivity analyses	9-10<br>N/A<br>Missing data were not addressed<br>N/A<br>7<br>N/A<br>No sensitivity analyses		
Data access and cleaning methods		..		RECORD 12.1: Authors should describe the extent to which the investigators had access to the database population used to create the study population.	7-8

For peer review only

				RECORD 12.2: Authors should provide information on the data cleaning methods used in the study.	8 and Figure 1
Linkage		..		RECORD 12.3: State whether the study included person-level, institutional-level, or other data linkage across two or more databases. The methods of linkage and methods of linkage quality evaluation should be provided.	7: No data linkage.
**Results**					
Participants	13	(a) Report the numbers of individuals at each stage of the study (*e.g.*, numbers potentially eligible, examined for eligibility, confirmed eligible, included in the study, completing follow-up, and analysed)<br>(b) Give reasons for non-participation at each stage.<br>(c) Consider use of a flow diagram	Figure 1 and page 7	RECORD 13.1: Describe in detail the selection of the persons included in the study (*i.e.,* study population selection) including filtering based on data quality, data availability and linkage. The selection of included persons can be described in the text and/or by means of the study flow diagram.	Figure 1 and page 7
Descriptive data	14	(a) Give characteristics of study participants (*e.g.*, demographic, clinical, social) and information on exposures and potential confounders<br>(b) Indicate the number of participants with missing data for each variable of interest<br>(c) *Cohort study* - summarise follow-up time (*e.g.*, average and total amount)	11<br><br>Missingness not assessed because of confounding of missing with negative values.		
Outcome data	15	*Cohort study* - Report numbers of outcome events or summary measures over time<br>*Case-control study* - Report numbers in each exposure	 11 and Table 1		

For peer review only - http://bmjopen.bmj.com/site/about/guidelines.xhtml

		category, or summary measures of exposure<br>*Cross-sectional study* - Report numbers of outcome events or summary measures			
Main results	16	(a) Give unadjusted estimates and, if applicable, confounder-adjusted estimates and their precision (e.g., 95% confidence interval). Make clear which confounders were adjusted for and why they were included<br>(b) Report category boundaries when continuous variables were categorized<br>(c) If relevant, consider translating estimates of relative risk into absolute risk for a meaningful time period	a) Relative estimates of feature weighting in the classification given in Table 3.<br>b) N/A<br>c) N/A		
Other analyses	17	Report other analyses done—e.g., analyses of subgroups and interactions, and sensitivity analyses	N/A		
**Discussion**					
Key results	18	Summarise key results with reference to study objectives	13-14		
Limitations	19	Discuss limitations of the study, taking into account sources of potential bias or imprecision. Discuss both direction and magnitude of any potential bias	16-17	RECORD 19.1: Discuss the implications of using data that were not created or collected to answer the specific research question(s). Include discussion of misclassification bias, unmeasured confounding, missing data, and changing eligibility over time, as they pertain to the study being reported.	16-17
Interpretation	20	Give a cautious overall interpretation of results considering objectives,	17		

For peer review only

		limitations, multiplicity of analyses, results from similar studies, and other relevant evidence			
Generalisability	21	Discuss the generalisability (external validity) of the study results	16		
**Other Information**					
Funding	22	Give the source of funding and the role of the funders for the present study and, if applicable, for the original study on which the present article is based	18		
Accessibility of protocol, raw data, and programming code		..		RECORD 22.1: Authors should provide information on how to access any supplemental information such as the study protocol, raw data, or programming code.	Protocol will be supplied on application to authors.

*Reference: Benchimol EI, Smeeth L, Guttmann A, Harron K, Moher D, Petersen I, Sørensen HT, von Elm E, Langan SM, the RECORD Working Committee. The REporting of studies Conducted using Observational Routinely-collected health Data (RECORD) Statement. *PLoS Medicine* 2015; in press.

*Checklist is protected under Creative Commons Attribution (CC BY) license.

For peer review only

BMJ Open

Automated detection of patients with dementia whose symptoms have been identified in primary care but have no formal diagnosis: a retrospective case-control study using electronic primary care records

Journal:	*BMJ Open*
Manuscript ID	bmjopen-2020-039248.R2
Article Type:	Original research
Date Submitted by the Author:	14-Dec-2020
Complete List of Authors:	Ford, Elizabeth; Brighton and Sussex Medical School, Department of Primary Care and Public Health<br>Sheppard, Joanne; University of Sussex School of Mathematical and Physical Sciences, Department of Physics and Astronomy; UCL, Medical Physics and Biomedical Engineering<br>Oliver, Seb; University of Sussex School of Mathematical and Physical Sciences, Department of Physics and Astronomy<br>Rooney, Philip; University of Sussex School of Mathematical and Physical Sciences, Department of Physics and Astronomy<br>Banerjee, Sube; University of Plymouth, Faculty of Health<br>Cassell, Jackie; Brighton and Sussex Medical School, Department of Primary Care and Public Health
**Primary Subject Heading**:	Health informatics
Secondary Subject Heading:	Diagnostics, General practice / Family practice, Geriatric medicine, Neurology
Keywords:	Dementia < NEUROLOGY, Health informatics < BIOTECHNOLOGY & BIOINFORMATICS, GERIATRIC MEDICINE, PRIMARY CARE, Delirium & cognitive disorders < PSYCHIATRY, Old age psychiatry < PSYCHIATRY

SCHOLARONE™
Manuscripts

I, the Submitting Author has the right to grant and does grant on behalf of all authors of the Work (as defined in the below author licence), an exclusive licence and/or a non-exclusive licence for contributions from authors who are: i) UK Crown employees; ii) where BMJ has agreed a CC-BY licence shall apply, and/or iii) in accordance with the terms applicable for US Federal Government officers or employees acting as part of their official duties; on a worldwide, perpetual, irrevocable, royalty-free basis to BMJ Publishing Group Ltd ("BMJ") its licensees and where the relevant Journal is co-owned by BMJ to the co-owners of the Journal, to publish the Work in this journal and any other BMJ products and to exploit all rights, as set out in our licence.

The Submitting Author accepts and understands that any supply made under these terms is made by BMJ to the Submitting Author unless you are acting as an employee on behalf of your employer or a postgraduate student of an affiliated institution which is paying any applicable article publishing charge ("APC") for Open Access articles. Where the Submitting Author wishes to make the Work available on an Open Access basis (and intends to pay the relevant APC), the terms of reuse of such Open Access shall be governed by a Creative Commons licence – details of these licences and which Creative Commons licence will apply to this Work are set out in our licence referred to above.

Other than as permitted in any relevant BMJ Author's Self Archiving Policies, I confirm this Work has not been accepted for publication elsewhere, is not being considered for publication elsewhere and does not duplicate material already published. I confirm all authors consent to publication of this Work and authorise the granting of this licence.

For peer review only

Automated detection of patients with dementia whose symptoms have been identified in primary care but have no formal diagnosis: a retrospective case-control study using electronic primary care records

Elizabeth Ford[1]
Joanne Sheppard [2,3]
Seb Oliver[2]
Philip Rooney[2]
Sube Banerjee[4]
Jackie Cassell[1]

1) Department of Primary Care and Public Health, Brighton and Sussex Medical School
2) Department of Physics and Astronomy, University of Sussex.
3) Medical Physics and Biomedical Engineering, University College London
4) Faculty of Health, University of Plymouth

Corresponding Author:
Elizabeth Ford
Department of Primary Care and Public Health, Brighton and Sussex Medical School
Watson Building, Village Way, Falmer
Brighton BN1 9PH
Tel: 01273 641974 email: e.m.ford@bsms.ac.uk

Keywords
Dementia; early diagnosis; primary care; electronic health records; machine learning; free text.

For peer review only

For peer review only - http://bmjopen.bmj.com/site/about/guidelines.xhtml

Abstract

Objectives: United Kingdom (UK) statistics suggest only two-thirds of patients with dementia get a diagnosis recorded in primary care. General practitioners (GPs) report barriers to formally diagnosing dementia, so some patients may be known by the GP to have dementia but missing a diagnosis in their patient record. We aimed to produce a method to identify these "known but unlabelled" patients with dementia using data from primary care patient records.

Design: Retrospective case-control study using routinely collected primary care patient records from CPRD.

Setting: UK general practice.

Participants: English patients aged >65y, with a coded diagnosis of dementia recorded 2000-2012 (cases); matched 1:1 to patients with no diagnosis code for dementia (controls).

Interventions: 8 coded and 9 keyword concepts indicating symptoms, screening tests, referrals and care for dementia recorded in the 5 years before diagnosis. We trialled machine learning classifiers to discriminate between cases and controls (logistic regression, naïve Bayes, random forest).

Primary and Secondary Outcomes: Outcome variable was dementia diagnosis code; accuracy of classifiers was assessed using Area Under the Receiver Operating Characteristic (AUC); order of features contributing to discrimination was examined.

Results: 93,426 patients were included; median age 83 years (64.8% female). Three classifiers achieved high discrimination and performed very similarly. AUCs were 0.87-0.90 with coded variables rising to 0.90-0.94 with keywords added. Feature prioritisation was different for each classifier, commonly prioritised features were Alzheimer's prescription, dementia annual review, memory loss, and dementia keywords.

Conclusions: It is possible to detect patients with dementia who are known to GPs but unlabelled with a diagnostic code, with a high degree of accuracy in electronic primary care record data. Using keywords from clinic notes and letters improves accuracy compared to coded data alone. This approach could improve identification of dementia cases for record-keeping, service planning, and delivery of good quality care.

For peer review only - http://bmjopen.bmj.com/site/about/guidelines.xhtml

Strengths and Limitations of this Study:

- Our study was representative of the UK population, using all patients in the CPRD GOLD database who were over 65 years old and had a first recorded dementia diagnosis in 2000-2012 (N= 46,713).
- However, the data used is several years old and there may have been changes in clinical practice since.
- We trialled a number of binary classifiers, but combining machine learning approaches might have increased the accuracy achieved.
- The approach was additionally strengthened by the addition of keywords from clinic notes and text to coded information.
- A limitation is that our model was trained on cases who all received a dementia diagnosis code at the end of the study period, who may differ systematically from those who never receive a code.

For peer review only

Introduction

Dementia is a term used to describe a group of conditions characterised by a progressive decline in cognitive function and consequent functional limitation.[1] In the United Kingdom (UK), 1.3% of people live with dementia, the majority of whom are over 65 years. Prevalence in all over 65s is 7.1%, rising from 1.8% in the 65-69 age group to over 33% in the over 90s.[2] The challenges of dementia affect individuals, their family carers, health and care services and systems and society as a whole, and addressing them has become a national and international priority due to population ageing. It is expected that the cost of the disease to society will double between 2014 and 2040, reaching £55bn per year in the UK.[2]

The UK government has recognised the benefits of an early diagnosis of dementia and has made improving rates of diagnosis a key focus of dementia policy since 2009 with the National Dementia Strategy 2009 [3] and the Prime Minister's Challenges on Dementia in 2012 and 2020.[4, 5] Whilst the disease is incurable, a diagnosis gives the patient a better chance of achieving a good quality of life while living with dementia.[6] Individuals can plan and make their wishes known while they still have capacity. Services provided by health and social care professionals may help people with dementia to adapt their home and identify their care needs, and thus avoiding or delaying having to move into institutionalised care. Furthermore, diagnosis allows people with Alzheimer's disease to be prescribed medication and non-pharmaceutical treatments to maximise their cognitive function and quality of life. These treatments may have a particular benefit in the early stages of the illness.[7, 8]

In recognition of this, general practitioners (GPs) in the UK are incentivised to initiate the diagnosis of dementia and to keep a register of patients diagnosed with dementia so that they can provide care proactively.[9] GPs are likely to be the first to recognise or be consulted about dementia symptoms, as 98% of the UK population is registered with a GP to receive primary healthcare services. If GPs suspect dementia they refer the patient to a specialist memory assessment service where a full diagnostic screening is carried out. If the patient is diagnosed with dementia, this is communicated back to the GP who then continues the patient's care. GPs are reimbursed for ensuring a face-to-face review for each patient with dementia every 12 months. Data collected in GP patient records about dementia cases (using a prescribed set of Dementia Read codes) is used by local Clinical Commissioning Groups (CCGs) as well as national bodies such as NHS England and Public Health England to monitor local and national diagnosis rates and services.[10]

For peer review only - http://bmjopen.bmj.com/site/about/guidelines.xhtml

Based on an epidemiological study conducted by the Medical Research Council in 1998 a prevalence of 6.6% was estimated in people over the age of 65.[11] However, based on dementia registers in the UK, the observed prevalence was found to be 3%.[11] This gap has closed substantially, due to schemes like the National Dementia Strategy,[3, 12] but coded diagnoses of dementia in GP patient records still only identify two-thirds of cases, compared to the expected prevalence,[10, 13] and the estimated median time to diagnosis being recorded from dementia onset is 3.5 years.[14] While the diagnosis rate in the UK is higher than in many countries (for example Lang et al.,[15] found an average rate of undetected dementia of 62% across Europe, North America and Asia) a third of people with dementia may not be receiving appropriate care.

Qualitative studies with GPs have indicated that GPs are sometimes reluctant to diagnose dementia,[16-18] and describe dementia as “a complex condition that takes time to diagnose”.[19] GPs have reported that diagnosing dementia early was not particularly important, and may in fact be harmful to some patients.[19] GPs tread carefully when initiating and communicating a diagnosis of dementia to their patients, as it is a stigmatised condition with what seems a bleak prognosis.[18, 20] Some researchers have argued that there is not enough evidence for any intervention that provides a positive change in prognosis or well-being for individuals with dementia and therefore a drive for earlier diagnosis is not justified.[21] In addition, as elderly patients often have multiple conditions, GPs report that it is difficult to integrate multiple clinical guidelines,[22] and that “dementia or memory problems [are] right at the bottom of the list”.[23] These findings suggest that patients with undiagnosed dementia could fall into two groups. First, those whose memory loss or cognitive symptoms have not been identified by the GP, possibly because they do not present to the GP very often, or they have not communicated any memory related symptoms. Second, there is likely to be a group of patients who are known by primary care clinicians to be experiencing memory problems, and may even have been screened for dementia or referred for memory assessment, but who have not, for many possible reasons, received a diagnostic code for dementia in their GP record.[16, 20, 24] For these patients who are “known but unlabelled”, there may be other indicators of dementia recognition and diagnosis in the patient record which could enable them to be detected using an algorithm which combines multiple indicators of the disease. Following identification, the GP could then decide if “labelling” these patients with a dementia code was appropriate.

For peer review only - http://bmjopen.bmj.com/site/about/guidelines.xhtml

Reviews of data-driven dementia risk prediction models,[25, 26] show that the majority of work in the past has aimed at producing risk scores which estimate the patient's risk of developing dementia at some future date. Only a few of these risk scores have been developed on routinely collected primary care data of which, a notable example is Walters et al.[27] Automated early detection of emergent cases has received less attention. Few attempts have been made to produce models which detect dementia which is current but unrecognised by the GP; the only examples using UK primary care data (known to the authors) are currently Jammeh et al.,[28] and Ford et al.[29, 30] Other studies have approached this problem manually, for example Russell et al.,[31] aimed to improve recording in dementia registers by a hand-searching exercise, recognising the need to "'clean up dementia coding and records". This successful exercise increased the identification rate for dementia in participating GP practices by 8.8%.

Following success in detecting unrecognised patients with dementia,[29] in this study we aimed to identify a range of elements in the primary care record which would identify the second group of "known but unlabelled" patients with dementia. A second aim was to evaluate whether the model could be improved by the use of keyword information in clinic notes and letters found in the patient records, rather than relying solely on information captured in clinical codes. Automatically detecting these patients may help with improving the quality of data in dementia registers, enabling ongoing care and support of individual patients with dementia, and giving commissioning bodies and service planners more accurate prevalence estimates.

Methods

Study Design

A retrospective case-control study using routinely collected primary care patient records from England, reported according to RECORD guidelines.[32]

Data Source

This study used data from the UK Clinical Practice Research Datalink (CPRD). It was established in 1987, and now contains anonymized healthcare records from more than 7 million current patients and represents up to 13% of the UK population.[33, 34] Patients are broadly representative of the UK general population in terms of age, sex and ethnicity. CPRD includes longitudinal observational data from general practitioner (GP) electronic health record systems in primary care practices, including

For peer review only

For peer review only - http://bmjopen.bmj.com/site/about/guidelines.xhtml

medical diagnoses, referrals to specialists and to secondary care, testing and interventional procedures conducted in primary care, lifestyle information (e.g. smoking, exercise) and drugs prescribed in primary care.[34] Data are captured using a structured hierarchical vocabulary called Read Codes, these were developed by a UK GP, Dr James Read, in the 1980s.[35] They map to other nomenclatures such as ICD, SNOMED-CT, and International Classification of Primary Care (ICPC) codes. Each Read code represents a term or short phrase describing a health-related concept. There are over 200,000 different codes, which are sorted into categories (diagnoses, processes of care and medication) and subchapters.[36] Each clinical entity is represented by an alphanumeric code and a Read term which is the plain language description. CPRD curates two primary care databases, one is formed of patient records derived from a GP patient record computer software called VISION (CPRD GOLD) and one is formed from records derived from a GP patient record computer software called EMIS (CPRD AURUM) [33]. This study used only CPRD GOLD data.

GPs also record clinical information in free text clinic notes and letters. CPRD held these clinic notes at the time of study data extraction, and made these available via either keyword search or release of full text for small samples, following 3-stage manual de-identification of text and approval of a protocol. Since 2013 free text notes and letters have not been extracted into CPRD from GP practices, and historic clinic text became unavailable to researchers in 2016. Due to this cessation of free text collection, the current study used data for patients who had a first dementia diagnosis recorded up to the end of 2012, so that free text was available for the full period prior to their diagnosis CPRD also offers data linkages with other NHS and administrative datasets but no linked data were used in this study.

Study Population

CPRD provided the study team with longitudinal data from patients with a dementia code (cases) recorded between 2000-2012, as defined in a code list of dementia diagnostic codes (general dementia, vascular and Alzheimer's dementia codes) that we developed drawing on code lists in Russell et al.,[31] and Rait et al.,[37] and reported in Ford et al.[29] (Appendix 1) For data extraction by the CPRD team, patients were required to be 65 years or older, and were required to have records available for at least three years prior to a first diagnosis code recorded between 2000 and 2012. All patients in the CPRD GOLD database meeting these criteria were selected to maximise sample size and reduce the possibility of selection bias. Control patients were randomly sampled from patients who had no dementia codes anywhere in their record (to reduce the likelihood that they just had a delayed diagnosis), who matched

For peer review only - http://bmjopen.bmj.com/site/about/guidelines.xhtml

cases on age, sex, and general practice, and who also had a minimum of three years' of records before the diagnosis date of their matched case (known as the "index date"). This resulted in a near-perfect 1-to-1 match between cases and controls, a few cases did not receive a match. No minimum duration of follow up after index date was required, as we did not want to bias the sample towards healthier individuals or exclude those who moved home or went into residential care (and thus changed GP practice) during follow up. Thus patients could have a date of death during the study as long as this was after the index date. Data collection on all patients was limited to November 2013 as this was the date of the final CPRD Gold database build which included free text. This resulted in 47,858 cases and 47,663 controls.

The entire coded patient record was extracted for each patient, including all clinical codes, from the patient's earliest registration date in CPRD until the date of data extraction (November 2013), or the patient's date or death or the date they left the practice, whichever was earlier. In addition, CPRD ran a search through free text notes and letters for keywords for all patients (described below) in the 24 months prior to the index date. For the keyword search, CPRD returned structured data comprised of a count of the keywords in each keyword group, found in each free text entry in the record. The study team did not have access to the free text of any patient for this study.

Refining participants

To standardise the dataset provided by CPRD, and to optimise it for the planned analysis, further refinements were made to the sample. From the extracted dataset, cases without matched controls, and all patients lacking five years of data prior to index date were removed along with their corresponding match. We removed cases for whom we could not find a dementia read code within the study period, and controls who did have a dementia code, and removed these along with their matched patients. See Figure 1 for a flowchart of final participant selection.

- insert Figure 1 here -

Legend: Figure 1: Final Participant Selection.

Feature selection

Code lists:

For peer review only - http://bmjopen.bmj.com/site/about/guidelines.xhtml

Using an expert consensus approach, with an epidemiologist, a psychiatrist and a GP, we identified a list of 8 potential clinical concepts which would be indicators of the onset of dementia, initiation of the diagnostic pathway for dementia or evidence of continuing care for dementia, and which would be captured within the Read codes. Our final list of eight coded concepts entered into each model were: cognitive decline, memory loss symptoms, mini-mental state examination (MMSE), other cognitive screening test, referral to memory clinic, referral to psychiatrist, neurologist or geriatrician, dementia annual review, and Alzheimer's specific prescription codes. Relevant code lists were sought from published papers, and where these were not available, lists were drawn up by author EF and checked by author PR (Appendix 2). Although MMSE scores are sometimes recorded in structured data, we found a majority of scores were missing in our dataset (56%). We decided to keep the model as simple as possible and therefore did not use MMSE scores in the analysis.

Keywords from clinic notes and letters:

We consulted with 17 GPs, asking them to suggest keywords representing terms that they might use when considering that a patient might be developing dementia. From these we identified 9 concepts: memory (suggested by 17 GPs); cognition (6 GPs); forgetful (14 GPs) MMSE or other screening test (6 GPs); confused (9 GPs); dementia (3 GPs); behaviour (5 GPs); family concern (6 GPs) and third party consultation (1 GP). We then generated a list of concept variations and spelling variations for each keyword group (Appendix 3).

Analysis

Only data from 5 years before the index date, up to the index date for each patient was used. Each patient's record was searched to find all instances of the Read codes referring to the eight features described above. If any codes were found for a feature within a patient's records, a dummy variable representing that feature was set to "1" otherwise they were defaulted to "0". Likewise, if evidence of keyword was found, the dummy variable for keywords were set to "1". A dummy variable for "any keyword" was also included. Other studies have also favoured binary features in order to reduce the influence on the data of the number of times a patient visits their GP.[28]

Logistic regression, random forest, and naive Bayes classifiers were used as they are effective supervised machine learning methods for the analysis and classification of binary data. Logistic regression uses the logit function to find the probability of a data point belonging to a (binary) group, using one or more nominal, ordinal, interval or ratio-level independent variables.[38] The addition of the LASSO function

For peer review only - http://bmjopen.bmj.com/site/about/guidelines.xhtml

selects the best predictors based on the residual sum of squares [39]. The random forest method is a non-parametric machine learning method for binary classification. It constructs a multitude of decision trees that best use the predictors to determine which category of output each case should be in. It holds the multitude of decision trees constant and the output for each individual is the mode or mean prediction class of the individual trees.[40] Naïve Bayes classifiers are based on Bayes Theorem.[41] Parameter estimation for naive Bayes models uses the method of maximum likelihood, and all predictive features are treated as contributing independently of each other (i.e. they are naïve to each other) to predict which category of the outcome each case belongs to.[42] These were implemented by using sci-kit learn with Python version 3.7.1 with the "LogisticRegression", "BernoulliNB" and "RandomForestClassifer" packages, with the seed set to 42 for reproducibility. The dataset was split into a 70% set for training the classifiers and a 30% testing set to assess model accuracy.

To assess the accuracy of the models the Area Under the Receiver Operator Curve (AUC) was calculated, which plots the true positive rate against the false positive rate for every possible cut-off of the predictive model. In addition, sensitivity and specificity values were calculated for model cut-offs where these values were equally weighted. Positive predictive values (PPV) were also calculated. While sensitivity and specificity are fixed parameters of the test, PPV values are conditional on the prevalence of the condition in the tested sample. Given the matched case-control design, our sample had a dementia prevalence of 50%, which would inflate PPV values artificially. We therefore separately calculated the PPV of the model using the estimated UK prevalence of dementia in people over 65 years of 7.1%.[2] This was achieved using the values of sensitivity and specificity calculated above, and a hypothetical sample of 100,000 patients where 7100 had dementia.

To assess which features contributed most to the models, we generated relative indices, with the most predictive feature weighting set at 1, and other features shown relative to this. This was instead of reporting the exact outputs from each machine learning algorithm (such as β weights from logistic regression), because we wanted models to be directly compared.

Results

Study Participants

For peer review only - http://bmjopen.bmj.com/site/about/guidelines.xhtml

The final dataset consisted of 93,426 patients with a median age of 83 years old (range 65-110). There were 32,876 male participants (35.2%) with a median age of 81 years old (range 65-100), and 60,550 female participants (64.8%) with a median age of 83 years old (range 65-110). 46,713 were cases and 46,713 were controls. The mean number of codes recorded per patient in the 5 years prior to index date was 614 for the controls and 586 for the cases (range 0-7,281).

Numbers of features by case and control group

Cases had much higher rates of all chosen features, as can be seen in Table 1. The most common coded feature in dementia cases was memory loss codes (46%), followed by dementia annual review codes (37%). Keywords were all common throughout cases, the most frequent were family concern (81%), memory (58%) and dementia (51%). All keywords were more common in cases than controls, except for third party consultation, and family concern was also high in controls (51%), although not as high as in the cases.

Table 1: Number and proportion of cases and controls who had each feature.

Feature	N cases with features (%) (N = 46,713)	N controls with feature (%) (N = 46,713)
**Codes**		
Cognitive decline codes	3,276 (7.01)	240 (0.51)
Cognitive screening test	888 (1.90)	124 (0.27)
Dementia annual review	17,372 (37.19)	151 (0.32)
Memory loss codes	21,282 (45.56)	1,387 (2.97)
MMSE test	9,574 (20.32)	600 (1.28)
Referral to memory assessment service	3,464 (7.42)	117 (0.25)
Referral to old age psychiatrist, neurologist or geriatrician	12,683 (27.15)	2,227 (4.77)
Alzheimer's medication prescription	10,044 (21.50)	203 (0.43)
**Keywords**		
Memory	27,025 (57.85)	3,815 (8.17)
Cognition	8,873 (18.99)	2,178 (4.66)
Forgetful	14,272 (30.55)	3,397 (7.27)
MMSE or screening test	11,969 (25.62)	2,189 (4.69)
Confused	20,530 (43.95)	5,334 (11.42)
Dementia	23,935 (51.24)	2,848 (6.10)
Behaviour change/problems	5,388 (11.53)	2,132 (4.56)
Family concerned	37,779 (80.87)	23,718 (50.77)
Third party consultation	706 (1.51)	1,854 (3.97)

Model Accuracy

For peer review only - http://bmjopen.bmj.com/site/about/guidelines.xhtml

The eight coded clinical concepts were used to train the three classifiers to predict which patients' record would end with a dementia diagnosis, and which patients' would not. Trained models were then tested on the testing dataset and accuracy of discrimination was assessed. Using only coded variables, the three models produced a high level of correct discrimination between cases and controls. The three classifiers performed very similarly with AUCs of 0.87-0.90 (Table 2 and Figure 2).

We then added the nine keywords to the code-only models, and found that discrimination improved up to an AUC of 0.94 in the logistic regression and random forest and 0.90 in the naïve Bayes classifier (Figure 3). It can be seen in Table 2 that the inclusion of the keywords increased the sensitivity of the models by around 8%, without any detrimental effect on specificity.

Table 2: Discrimination of three models between cases and controls

	Codes only				Codes and Keywords			
Classifier	AUC	Sensitivity	Specificity	PPV at 7.1% prevalence	AUC	Sensitivity	Specificity	PPV at 7.1% prevalence
Random Forest	0.90	0.77	0.92	0.46	0.94	0.85	0.92	0.46
Logistic Regression	0.90	0.76	0.93	0.49	0.94	0.84	0.93	0.51
Naive Bayes	0.87	0.78	0.91	0.45	0.90	0.80	0.91	0.43

- insert Figure 2 here -

Legend: Figure 2: ROC showing discrimination between dementia cases and controls by three machine learning models with codes only.

- insert Figure 3 here -

Legend: Figure 3: ROC showing discrimination between dementia cases and controls by three machine learning models with both codes and keywords.

Feature weighting

The relative weighting of each feature in the different models, with the most predictive feature weighting set at 1, and other features shown relative to this, is shown in Table 3, separated by models for the coded data only and for codes and keywords together. The table shows that the three different models had substantial differences in the weighting of the different features when using only keywords.

For peer review only - http://bmjopen.bmj.com/site/about/guidelines.xhtml

The logistic regression weighted most highly dementia annual review codes and prescriptions of Alzheimer's medication, while the random forest weighted memory loss codes highest and the naïve Bayes classifier weighted referral for memory assessment as the most informative feature.

Looking at the combined codes and keywords, similar codes were most important in the three models, whereas the most important keyword was the word memory (rated highest feature in the random forest model), followed by keywords for dementia. Interestingly, the logistic regression classifier prioritised third party consultation, the only feature higher in the controls than the cases. The naïve Bayes classifier prioritised coded variables higher than all keywords.

Sensitivity Analysis

We re-ran the models removing controls with Alzheimer's medication prescriptions and dementia annual review codes. We also removed these two code lists as predictors in the model. Overall model accuracy, measured by AUC, was reduced for the codes-only models (AUCs: Log Reg 0.83; Random Forest 0.83; Naïve Bayes 0.83); this reflects the high contribution these two predictors made in the main models. However, in these models, specificity increased for all codes-only models to 0.95, which also resulted in higher PPVs (Log Reg 0.61; Random Forest 0.61; Naïve Bayes 0.60). Results for all performance parameters for models using both codes and keywords remained similar (AUCs: Log Reg 0.93; Random Forest 0.93; Naïve Bayes 0.89). Full results of the sensitivity analysis are given in Appendix 4.

For peer review only - http://bmjopen.bmj.com/site/about/guidelines.xhtml

Table 3: Relative weighting of each feature in the three classifiers

	Coded data only			Codes and Keywords		
	Logistic Regression	Random Forest	Naive Bayes	Logistic Regression	Random Forest	Naive Bayes
Alzheimer's Medication	0.727	0.354	0.912	0.676	0.339	0.912
Cognitive Decline	0.378	0.066	0.888	0.260	0.061	0.880
Cognitive Screening Test	0.025	0.006	0.997	0.076	0.010	0.997
Dementia Annual Review	1	0.845	0.957	0.917	0.888	0.957
Memory Loss Codes	0.622	1	0.593	0.456	0.964	0.593
MMSE	0.344	0.187	0.729	0.199	0.157	0.729
Referral To Memory Assessment	0.312	0.049	1	0.143	0.033	1
Referral To Psych/Geri/Neuro	0.345	0.292	0.509	0.248	0.210	0.509
Keyword Dementia	-	-	-	0.494	0.975	0.473
Keyword Memory	-	-	-	0.251	1	0.422
Keyword Confusion	-	-	-	0.238	0.444	0.363
Keyword Behaviour	-	-	-	0.037	0.091	0.520
Keyword Cognition	-	-	-	0.031	0.078	0.515
Keyword Family	-	-	-	0.158	0.079	0.113
Keyword MMSE	-	-	-	0.019	0.119	0.515
Keyword Forget	-	-	-	0.051	0.087	0.439
Keyword Third party consultation	-	-	-	1	0.200	0.544

Discussion

We produced a set of binary coded variables that could be combined in a statistical model and which could accurately classify patients who went on to have a dementia diagnosis formally coded. This model could be take forward to the clinic to identify "known but unlabelled" patients with dementia in primary care, thus improving record keeping, prevalence estimates, and ongoing care for these patients. We also investigated whether adding in information from the clinic notes and letters to the model, in the form of a simple keyword extraction, could add further value to the approach. The addition of free text keywords improved the discrimination of the model to an excellent AUC of 0.94, suggesting this model would perform well in the clinic. The improved performance with the addition of free text keywords was achieved because of increased sensitivity of the model, with no loss of specificity, as has been shown in a range of other case-identification studies incorporating free text.[43] This study provides evidence that hidden data in these free text fields can substantially improve automated detection of cases, adding weight to arguments that free text data are valuable for research, if acceptable privacy preserving methods for data-sharing (such as automated information extraction behind a clinical firewall) can be agreed upon.[44] We tried three different machine learning approaches which are suitable for binary

For peer review only - http://bmjopen.bmj.com/site/about/guidelines.xhtml

data and found all performed similarly, although they weighted the various features differently in their classifications. This is likely to be due to the different construction of the algorithms within each method, for example, random forest algorithms are thought to weight most highly the commonest features in the dataset, rather than features which are rarer but differ more substantially between the two groups. Future work could look at whether there is added value in combining the three approaches, as ensemble methods can sometimes outperform any of the single approaches alone, especially where the different methods are clearly prioritising different features to achieve their performance. However, most have been trialled on multi-class rather than binary classification.[45]

We also chose not to clean up our control group for evidence of dementia medication prescribing as has been done in other studies, [46] because we felt that doing so would have artificially inflated the performance of our models. However, in the literature, multiple case definitions of dementia are used in primary care EHR research, some of which incorporate Alzheimer's medication prescriptions or dementia review codes. To test the impact of our case definition choice, we ran a sensitivity analysis, removing patients from the control group who had these codes, as well as taking the code lists out of models as predictors. As these two predictors had been highly weighted in the original models, model performance in the codes-only models dropped when they were removed, although sensitivity increased along with PPV. Interestingly however, there was very little drop in performance in the models which used both codes and keywords, suggesting the keywords could make up for the loss of these two sets of codes. The case definition most useful in practice will depend on how GPs make up the inclusion criteria for their dementia registers. Where GP practices use only dementia codes for populating their case register, an algorithm which searches for Alzheimer's medication prescriptions or dementia annual review will be helpful for spotting true positive cases among their unlabelled dementia patients.

Our study is a novel contribution to the literature. Studies have previously aimed to detect dementia that was unknown to primary care practitioners, by manual [47-49] and automated methods,[28] using data from primary care records. Additionally, a recent study by Aldus et al. [14] has explored rates and predictors of patients with dementia going undetected. However, to the authors' knowledge, no prediction or early detection approaches have made the distinction between patients with dementia unknown to the GP and those known to the GP but nevertheless unrecorded with a formal diagnostic code. The study by Aldus et al. gives the first indication of how many of the "undiagnosed" cohort may be "known but unlabelled", suggesting that both unknown and known but unlabelled groups decline over time, but that of patients with no diagnosis, around 66-71% were not known to have symptoms,

For peer review only - http://bmjopen.bmj.com/site/about/guidelines.xhtml

while 29-34% had memory concerns or a referral noted in their patient record.[14] These figures were based on small numbers and it would be worth investigating this further to estimate the proportions of each group. Our team has also used social science methods, interviewing and surveying GPs and patients to understand what is happening in this apparent “diagnosis gap” for dementia in UK primary care. We suggest that this dual “data science and social science” approach to understanding and utilising data in patient records for clinical decision or diagnostic support is valuable, as it exposes the underlying human decision-making and behaviour which influences how the data are created. These insights can be drawn on to improve clinical prediction models and make them more relevant for key stakeholders, which is likely to improve implementation. A further avenue for exploration with GPs, and other stakeholders such as service commissioners, is whether the results produced by this approach would be of value for either improving individual patient care, or for service and resource planning, or commissioning, due to more accurate prevalence rates in patient data.

Further work should also examine the ethics and desirability of such methods in practice. Previous studies have highlighted a range of potential ethical issues which need further exploration. Firstly, according to the principles of ethical screening by Wilson and Jungner [50] any kind of screening programme is only ethical if there is an accepted treatment for patients with recognised disease. Weatherby and Agius argue that given no treatments that modify disease trajectory for dementia are available, and given the possibility of false positive results, population screening for dementia is ethically unjustifiable, although a stepwise process that becomes increasingly targeted might be appropriate.[51] A stepwise approach to detection may be especially important given the low PPVs found when assessing our model against a population prevalence. Ienca et al. have also argued that use of big data for early detection may lead to an inflation of the false positive risk, presumably because of algorithms being applied indiscriminately on whole populations.[52] They also draw attention to the need for clinicians or researchers to be cautious about the privacy and disclosure issues of personal data about dementia, as this would be highly sensitive information.[52] Other authors have discussed the ethical implications of genetic testing for Alzheimer risk in early detection or predictive models,[51, 53] as well as needing to maintain the “right not to know” for patients.[54] Drawing on these debates and other literature, Thyrian et al. argue that early recognition is wanted by a majority of patients if offered in primary care, that GPs are also largely in favour,[55] and that evidence-based case finding algorithms may be ethically acceptable in primary care if tailored to both diagnostic procedures and, importantly, a post-diagnostic support package.[56]

For peer review only - http://bmjopen.bmj.com/site/about/guidelines.xhtml

Strength and limitations

This study used a large sample of English patients who were all over the age of 65, and patients registered in CPRD are thought to be representative of patients in England as a whole. However, to ensure that this model is robust to different patient groups, it should now be evaluated in a new patient sample, perhaps in one which is produced from a different general practice computer system (CPRD Gold data is harvested from VISION software). A further strength is that we specified a very simple model which dichotomised the presence of an indicator from a code list, this means the model can be readily operationalised in any GP record system and should be robust to number and order of code accrual in the patient record.

However, there are important limitations in our study design which could be assessed or addressed in future work. Notably, data collection was limited to cases identified before the end of 2012, and therefore it is unclear whether the same model would apply in current times, as the many government initiatives which have been implemented since our study's data collection may have changed clinical practice and thus recording. We plan future work to assess this, and to strengthen the analysis by using a cohort design and linked data from hospital records and death certificate data. We used a matched case-control design, in which the contribution of age and sex to the model was controlled for in the matching, and in which only one control for each dementia case was used. Using more controls per case would have increased statistical power and reduced the risk that controls were in fact undiagnosed dementia cases. We also chose not to use MMSE score, as we aimed to create simple models with binary predictors, and because the majority of MMSE scores were missing from structured data. A future cohort study where the prevalence is lower, where age can be added as a predictor, where possible false negatives can be removed from the control group, and which could explore more complex predictors such as MMSE score, will be a robust assessment of this model's replicability.

A further limitation is that our model was trained on cases who all received a dementia diagnosis code at the end of the study period. There are likely to be a number of dementia cases who never receive a dementia code, and it remains a possibility that these patients differ in systematic ways from cases who do go on to receive a code. The ability of our model to detect patients who never receive a code is therefore unknown. One possibility is that patients detected by our model will have mild cognitive impairment or another non-dementia memory complaint, this may especially be true given the low expected PPV in an unselected population of over 65s. An evaluation in a GP practice, where the

For peer review only - http://bmjopen.bmj.com/site/about/guidelines.xhtml

predictions of the model are assessed and validated by the practising GP, would give us further information about its performance in real-life clinic settings, and would help us understand more about potential false positives flagged by the model. Finally, we used a very simple keyword extraction to access the keyword data, and did not incorporate any natural language processing to identify negation, hedging, subject or timing of the words we identified. We may therefore have over-sampled instances of the clinical concepts being recorded about the patients, however this would have affected both cases and controls equally and is most likely to have resulted in a bias towards the null. It was important for our study design that we chose simple feature extraction methods which could potentially be recreated in a busy clinic.

Implications and future steps

Our model shows good accuracy in identifying dementia patients who have been detected as likely to have dementia but who currently have no diagnostic code for the condition. As outlined above, two further validation studies are now indicated; first, a replication in a cohort study using similar primary care patient data and second, a proof of concept trial in a local GP practice or primary care network. We hope if these two further pieces of work indicate the same excellent discrimination between patients with dementia and those without, and following steps to determine the threshold resulting in high specificity and sensitivity, and identifying reasons for potential false positives, this model could be adopted by GPs and CCGs to improve the quality of their dementia registers and to aid service planning.

These findings also provide a potential way for EHR researchers to extend their dementia case identification when using primary care data for dementia research and potentially to improve research quality. Emerging evidence on the rates of undetected dementia [14] as well as novel methods for identifying probable but unlabelled cases such as those reported here, mean that researchers could run sensitivity analyses on a range of more and less sensitive dementia definitions, rather than rely on diagnosis codes alone. We suggest exploring combinations of memory loss symptoms, Alzheimer specific prescriptions, and dementia annual review codes to extend the case definition of dementia in EHR research, and to conduct validation studies on these new case identification methods, and evaluate reasons for false positives. MMSE scores, where recorded, could be explored as an additional variable of particular value. As random forest and logistic regression models performed very similarly in this and other studies [29], we would suggest either analytical approach could be valuable for classification tasks in dementia EHR research.

For peer review only - http://bmjopen.bmj.com/site/about/guidelines.xhtml

Conclusions

We have shown that a simple model made up of indications of dementia symptoms, screening tests, referral and follow up care in coded and free text data can identify primary care patients who have recognised dementia but no diagnostic code for it in their record. This model can be taken forward for further validation, and used by GPs and commissioners to improve the quality of data on patients with dementia and as a result, improve dementia care and services.

Supplementary Materials

Appendix 1: Dementia Code List

Appendix 2: Features Code Lists

Appendix 3: List of Keywords

Appendix 4: RECORD Checklist

Acknowledgements

This work uses data provided by patients and collected by the NHS as part of their care and support. #datasaveslives.

Funder

This project was funded by a grant from the Wellcome Trust ref. 202133/Z/16/Z. The funder had no role in study design, data collection and analysis, decision to publish, or preparation of the manuscript.

Ethics approval and consent to participate

This study was approved by the Independent Scientific Advisory Committee at the Medicines and Healthcare Products Regulatory Authority, UK, protocol number 15_111_RA. Following approval, administrative permissions to access and use the electronic patient records were granted by Clinical Practice Research Datalink (CPRD.com).

Consent for publication

For EHR data: Not applicable

Patient and Public Involvement

Prior to applying for funding a focus group was conducted with people with dementia and their carers to guide the development of the research question. Patients were not involved in the design of the study or the acquisition of data. Results have been disseminated through a series of public presentations to patient interest groups, through a study website (www.bsms.ac.uk/astrodem) and through social media.

For peer review only

For peer review only - http://bmjopen.bmj.com/site/about/guidelines.xhtml

Availability of data and materials

The data that support the findings of this study are available from Clinical Practice Research Datalink (CPRD; www.cprd.com) but restrictions apply to the availability of these data, which were used under license for the current study, and so are not publicly available. For re-using these data, an application must be made directly to CPRD.

Authors' contributions

EF, SO and JC conceived and directed the study. EF drew up code lists and PR checked these. PR managed the data. JS conducted the analyses. PR and SO gave data analysis advice. SB and JC gave clinical advice. EF and JS wrote the manuscript. All authors provided critical feedback on the manuscript and approved the final version.

Competing interests

The authors declare that they have no competing interests.

References

1. Prince M, Jackson J, al. e. World Alzheimer Report 2009 London: Alzheimer's Disease International; 2009 [Available from: https://www.alz.co.uk/research/files/WorldAlzheimerReport.pdf].

2. Prince M, Knapp M, Guerchet M, McCrone P, Prina M, Comas-Herrera A, et al. Dementia UK Update London, UK2014 [Available from: https://www.alzheimers.org.uk/sites/default/files/migrate/downloads/dementia_uk_update.pdf].

3. Department of Health. Living well with dementia: A National Dementia Strategy 2009 [Available from: https://www.gov.uk/government/uploads/system/uploads/attachment_data/file/168220/dh_094051.pdf].

4. Department of Health. Prime Minister's challenge on dementia: Delivering major improvements in dementia care and research by 2015 2012 [Available from: https://www.gov.uk/government/uploads/system/uploads/attachment_data/file/215101/dh_133176.pdf].

5. Department of Health. Prime Minister's Challenge on Dementia 2020 [Available from: https://assets.publishing.service.gov.uk/government/uploads/system/uploads/attachment_data/file/414344/pm-dementia2020.pdf].

6. Prince M, Bryce R, Ferri C. World Alzheimer Report 2011: The benefits of early diagnosis and intervention: Alzheimer's Disease International; 2011.

For peer review only - http://bmjopen.bmj.com/site/about/guidelines.xhtml

7. Leifer BP. Early diagnosis of Alzheimer's disease: clinical and economic benefits. J Am Geriatr Soc. 2003;51(5s2):S281-S8.

8. Banerjee S. Living well with dementia—development of the national dementia strategy for England. Int J Geriatr Psychiatry. 2010;25(9):917-22.

9. NHS England. Enhanced Service Specification: Dementia Identification Scheme 2014 [Available from: http://www.england.nhs.uk/wp-content/uploads/2014/10/dementia-ident-schm-fin.pdf].

10. NHS England. Dementia diagnosis rate workbooks 2017 [Available from: https://www.england.nhs.uk/publication/dementia-diagnosis-rate-workbook/].

11. Matthews FE, Stephan BC, Robinson L, Jagger C, Barnes LE, Arthur A, et al. A two decade dementia incidence comparison from the Cognitive Function and Ageing Studies I and II. Nat Commun. 2016;7:11398.

12. Donegan K, Fox N, Black N, Livingston G, Banerjee S, Burns A. Trends in diagnosis and treatment for people with dementia in the UK from 2005 to 2015: a longitudinal retrospective cohort study. Lancet Public Health. 2017;2(3):e149-e56.

13. Low L-F, McGrath M, Swaffer K, Brodaty H. Communicating a diagnosis of dementia: A systematic mixed studies review of attitudes and practices of health practitioners. Dementia. 2019;18(7-8):2856-905.

14. Aldus CF, Arthur A, Dennington-Price A, Millac P, Richmond P, Dening T, et al. Undiagnosed dementia in primary care: a record linkage study. Health Services and Delivery Research. 2020;8(20).

15. Lang L, Clifford A, Wei L, Zhang D, Leung D, Augustine G, et al. Prevalence and determinants of undetected dementia in the community: a systematic literature review and a meta-analysis. BMJ Open. 2017;7(2):e011146.

16. Moore V, Cahill S. Diagnosis and disclosure of dementia—A comparative qualitative study of Irish and Swedish general practitioners. Aging & mental health. 2013;17(1):77-84.

17. Lahjibi-Paulet H, Dauffy A, Minard A, Gaxatte C, Saint-Jean O, Somme D. Attitudes toward Alzheimer's disease: a qualitative study of the role played by social representation on a convenient sample of French general practitioners. Aging Clin Exp Res. 2012;24(4):384-90.

18. Dhedhi SA, Swinglehurst D, Russell J. 'Timely' diagnosis of dementia: what does it mean? A narrative analysis of GPs' accounts. BMJ Open. 2014;4(3):e004439.

19. Hansen EC, Hughes C, Routley G, Robinson AL. General practitioners' experiences and understandings of diagnosing dementia: factors impacting on early diagnosis. Soc Sci Med. 2008;67(11):1776-83.

For peer review only - http://bmjopen.bmj.com/site/about/guidelines.xhtml

20. Pentzek M, Wollny A, Wiese B, Jessen F, Haller F, Maier W, et al. Apart from nihilism and stigma: what influences general practitioners' accuracy in identifying incident dementia? The American journal of geriatric psychiatry : official journal of the American Association for Geriatric Psychiatry. 2009;17(11):965-75.

21. Brayne C, Kelly S. Against the stream: early diagnosis of dementia, is it so desirable? BJPsych bulletin. 2019;43(3):123-5.

22. Pimlott NJG, Persaud M, Drummond N, Cohen CA, Silvius JL, Seigel K, et al. Family physicians and dementia in Canada Part 2. Understanding the challenges of dementia care. Can Fam Physician. 2009;55(5):508-U64.

23. Boise L, Camicioli R, Morgan DL, Rose JH, Congleton L. Diagnosing dementia: Perspectives of primary care physicians. Gerontologist. 1999;39(4):457-64.

24. Iliffe S, Manthorpe J, Eden A. Sooner or later? Issues in the early diagnosis of dementia in general practice: a qualitative study. Fam Pract. 2003;20(4):376-81.

25. Stephan BC, Tang E, Muniz-Terrera G. Composite risk scores for predicting dementia. Curr Opin Psychiatry. 2016;29(2):174-80.

26. The PHG Foundation. Dementia Risk Prediction Models: What do policy makers need to know? Cambridge, UK: The University of Cambridge; 2019 [Available from: https://www.phgfoundation.org/documents/dementia-risk-prediction-models.pdf].

27. Walters K, Hardoon S, Petersen I, Iliffe S, Omar RZ, Nazareth I, et al. Predicting dementia risk in primary care: development and validation of the Dementia Risk Score using routinely collected data. BMC Medicine. 2016;14(1):1-12.

28. Jammeh EA, Camille BC, Stephen WP, Escudero J, Anastasiou A, Zhao P, et al. Machine-learning based identification of undiagnosed dementia in primary care: a feasibility study. BJGP Open. 2018;2(2):bjgpopen18X101589.

29. Ford E, Rooney P, Oliver S, Hoile R, Hurley P, Banerjee S, et al. Identifying undetected dementia in UK primary care patients: a retrospective case-control study comparing machine-learning and standard epidemiological approaches. BMC Med Inform Decis Mak. 2019;19(1):248.

30. Ford E, Starlinger J, Rooney P, Oliver S, Banerjee S, van Marwijk H, et al. Could dementia be detected from UK primary care patients' records by simple automated methods earlier than by the treating physician? A retrospective case-control study. Wellcome Open Res. 2020;5(120):120.

For peer review only

31. Russell P, Banerjee S, Watt J, Adleman R, Agoe B, Burnie N, et al. Improving the identification of people with dementia in primary care: evaluation of the impact of primary care dementia coding guidance on identified prevalence. BMJ Open. 2013;3(12).

32. Benchimol EI, Smeeth L, Guttmann A, Harron K, Moher D, Petersen I, et al. The REporting of studies Conducted using Observational Routinely-collected health Data (RECORD) Statement. PLOS Medicine. 2015;12(10):e1001885.

33. Wolf A, Dedman D, Campbell J, Booth H, Lunn D, Chapman J, et al. Data resource profile: Clinical Practice Research Datalink (CPRD) Aurum. Int J Epidemiol. 2019;48(6):1740-g.

34. Herrett E, Gallagher AM, Bhaskaran K, Forbes H, Mathur R, van Staa T, et al. Data resource profile: clinical practice research datalink (CPRD). Int J Epidemiol. 2015;44(3):827-36.

35. Chisholm J. The Read clinical classification. Brit Med J. 1990;300:1092.

36. Booth N. What are the Read Codes? Health Libr Rev. 1994;11(3):177-82.

37. Rait G, Walters K, Bottomley C, Petersen I, Iliffe S, Nazareth I. Survival of people with clinical diagnosis of dementia in primary care: cohort study. Brit Med J. 2010;341:c3584.

38. Wiest MM, Lee KJ, Carlin JB. Statistics for clinicians: An introduction to logistic regression. J Paediatr Child Health. 2015;51(7):670-3.

39. Tibshirani R. Regression shrinkage and selection via the LASSO. J R Stat Soc Series B Stat Methodol. 1996:267-88.

40. Alam MZ, Rahman MS, Rahman MS. A Random Forest based predictor for medical data classification using feature ranking. Inform Med Unlocked. 2019;15:100180.

41. Stone JV. Bayes' rule: a tutorial introduction to Bayesian analysis: Sebtel Press; 2013.

42. Langarizadeh M, Moghbeli F. Applying Naive Bayesian Networks to Disease Prediction: a Systematic Review. Acta informatica medica. 2016;24(5):364-9.

43. Ford E, Carroll JA, Smith H, Scott D, Cassell J. Extracting information from the text of electronic medical records to improve case detection: a systematic review. J Am Med Inform Assoc. 2016;doi: 10.1093/jamia/ocv180.

44. Jones KH, Ford EM, Lea N, Griffiths LJ, Hassan L, Heys S, et al. Toward the Development of Data Governance Standards for Using Clinical Free-Text Data in Health Research: Position Paper. J Med Internet Res. 2020;22(6):e16760.

45. Galar M, Fernández A, Barrenechea E, Bustince H, Herrera F. An overview of ensemble methods for binary classifiers in multi-class problems: Experimental study on one-vs-one and one-vs-all schemes. Pattern Recognition. 2011;44(8):1761-76.

For peer review only - http://bmjopen.bmj.com/site/about/guidelines.xhtml

46. Ponjoan A, Garre-Olmo J, Blanch J, Fages E, Alves-Cabratosa L, Martí-Lluch R, et al. Epidemiology of dementia: prevalence and incidence estimates using validated electronic health records from primary care. Clin Epidemiol. 2019;11:217-28.

47. Banerjee S, Willis R, Matthews D, Contell F, Chan J, Murray J. Improving the quality of care for mild to moderate dementia: an evaluation of the Croydon Memory Service Model. Int J Geriatr Psychiatry. 2007;22(8):782-8.

48. Kallumpuram S, Kumar CS, Khan B, Gavins V, Khan A, Iliffe S. Targeted case finding for dementia in primary care: Surrey Downs dementia diagnosis project. BMJ Open Qual. 2015;4(1):u209827. w4086.

49. Wray LO, Wade M, Beehler GP, Hershey LA, Vair CL. A Program to Improve Detection of Undiagnosed Dementia in Primary Care and Its Association with Healthcare Utilization. The American journal of geriatric psychiatry : official journal of the American Association for Geriatric Psychiatry. 2014;22(11):1282-91.

50. Wilson JMG, Jungner G, Organization WH. Principles and practice of screening for disease. 1968.

51. Weatherby TJ, Agius M. Ethical and Organisational considerations in screening for dementia. Psychiatria Danubina. 2018;30(Suppl 7):463-8.

52. Ienca M, Vayena E, Blasimme A. Big Data and Dementia: Charting the Route Ahead for Research, Ethics, and Policy. Front Med. 2018;5(13).

53. Arribas-Ayllon M. The ethics of disclosing genetic diagnosis for Alzheimers disease: Do we need a new paradigm? Brit Med Bulletin. 2011;100:7-21.

54. Schweda M, Kögel A, Bartels C, Wiltfang J, Schneider A, Schicktanz S. Prediction and early detection of Alzheimer’s dementia: professional disclosure practices and ethical attitudes. J Alzheimers Dis. 2018;62(1):145-55.

55. Thyrian JR, Eichler T, Pooch A, Albuerne K, Dreier A, Michalowsky B, et al. Systematic, early identification of dementia and dementia care management are highly appreciated by general physicians in primary care - results within a cluster-randomized-controlled trial (DelpHi). J Multidiscip Healthc. 2016;9:183-90.

56. Thyrian JR, Hoffmann W, Eichler T. Early Recognition of Dementia in Primary Care-Current Issues and Concepts. Curr Alzheimer Res. 2018;15(1):2-4.

For peer review only

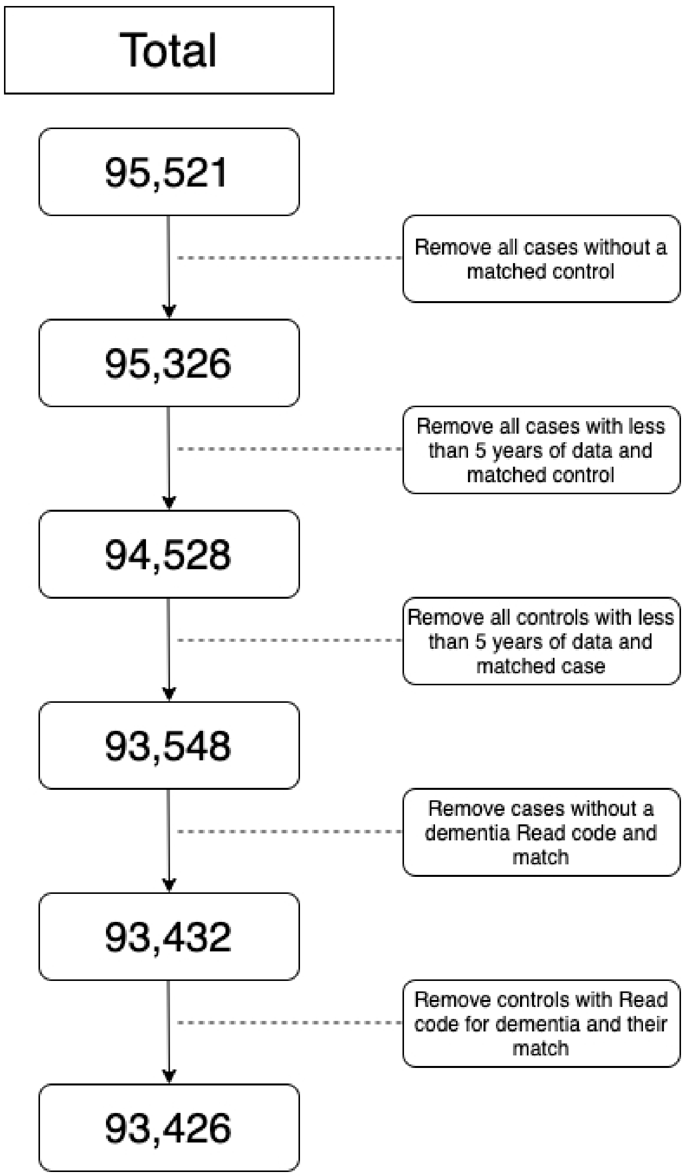

Figure 1: Final Participant Selection.

108x182mm (96 x 96 DPI)

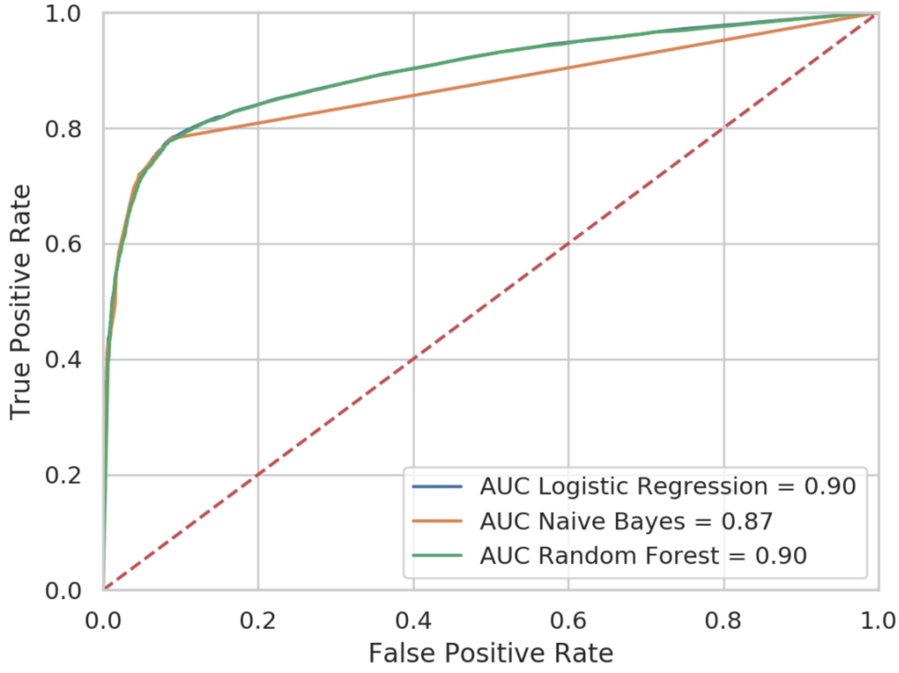

Figure 2: ROC showing discrimination between dementia cases and controls by three machine learning models with codes only.

338x233mm (96 x 96 DPI)

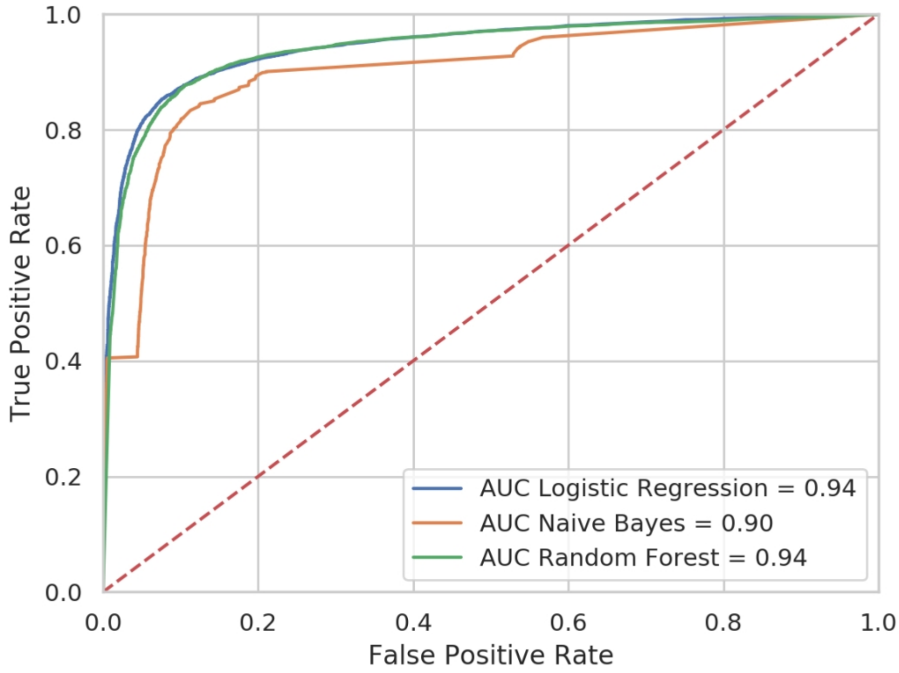

Figure 3: ROC showing discrimination between dementia cases and controls by three machine learning models with both codes and keywords.

338x233mm (96 x 96 DPI)

ANNEX 1 - DEMENTIA DIAGNOSTIC READ CODES

Read Description	Read Code	Medical Code
**Alzheimers codes**		
Dementia in Alzheimer's disease	Eu00	7664
Dementia in Alzheimer's disease with early onset	Eu000	49263
Dementia in Alzheimer's disease with late onset	Eu001	38678
Dementia in Alzheimer's disease, atypical or mixed type	Eu002	30706
Dementia in Alzheimer's disease unspecified	Eu00z	29386
Alzheimer's disease	F110	1917
Alzheimer's disease with early onset	F1100	16797
Alzheimer's disease with late onset	F1101	32057
[X]Presenile dementia,Alzheimer's type	Eu00011	25704
[X]Primary degen dementia, Alzheimer's type, presenile or	Eu00012	60059
[X]Alzheimer's disease type 2	Eu00013	61528
[X]Alzheimer's disease type 1	Eu00111	46762
[X]Senile dementia,Alzheimer's type	Eu00112	11379
[X]Primary degen dementia of Alzheimer's type, senile ons	Eu00113	43346
[X]Alzheimer's dementia unspec	Eu00z11	8195
	15	
**Vascular Dementia Codes**		
Vascular dementia	Eu01	6578
Arteriosclerotic dementia	E004	19477
Multi-infarct dementia	Eu011	8634
Subcortical vascular dementia	Eu012	8934
Mixed cortical and subcortical vascular dementia	Eu013	31016
Other vascular dementia	Eu01y	55313
Vascular dementia unspecified	Eu01z	19393
Uncomplicated arteriosclerotic dementia	Eu0040	43089
Arteriosclerotic dementia with delirium	Eu0041	56912
Arteriosclerotic dementia with paranoia	Eu0042	55467
Arteriosclerotic dementia with depression	Eu0043	43292
Arteriosclerotic dementia NOS	Eu004z	42279
[X]Vascular dementia of acute onset	Eu01000	46488
[X]Predominantly cortical dementia	Eu01111	55838
[X] Multi-infarct dementia	Eu01100	11175
	15	
**Dementia in other diseases**		
[X] Dementia in other diseases classified elsewhere	Eu02y00	64267
Dementia in Pick's disease	Eu020	28402
Circumscribed brain atrophy including Pick's disease	F111	?
Dementia in Creutzfeldt-Jackob disease	Eu021	54106
Dementia in Huntingdon's disease	Eu022	37014
Dementia in Parkinson's disease	Eu023	9509
Dementia in HIV disease	Eu024	4485
Dementia in conditions EC	E041.00	25386
Alcoholic dementia NOS	Eu10711	27342
[X] Alcoholic dementia NOS	Eu10711	26323
Other alcoholic dementia	E012	54505
Drug-induced dementia	E02y100	62132
Lewy body disease	F116	7572
Lewy body dementia	Eu025	26270
	14	
**Dementia not otherwise classified**		
Unspecified dementia	Eu02z	4693
Senile/Presenile dementia	Eu00..12	1350

For peer review only

Presenile dementia	E001	15165
Uncomplicated presenile dementia	E0010	46202
Presenile dementia with delirium	E0011	49513
Presenile dementia with paranoia	E0012	30032
Presenile dementia with depression	E0013	27677
Presenile dementia NOS	E0014	38438
[X] Presenile dementia NOS	Eu02z11	48501
Uncomplicated senile dementia	E000	7323
Senile dementia	E00..11	1916
Senile dementia with depressive or paranoid features	E002	44674
Senile dementia with paranoia	E0020	18386
Senile dementia with depression	E0021	21887
Senile dementia with depressive or paranoid features NOS	E002z	41089
Delirium superimposed on dementia	Eu041	53446
Senile dementia with delirium	E003	37015
Senile degeneration of the brain, not elsewhere classified	F112	29512
	18	
**Total N codes**	**62**	

For peer review only

Cognitive Decline

Read term	Read code	Medcode
Cognitive decline	28E..00	7674
GDS level 2 - very mild cognitive decline	3AE1.00	65856
GDS level 3 - mild cognitive decline	3AE2.00	60263
GDS level 4 - moderate cognitive decline	3AE3.00	60726
GDS level 5 - moderately severe cognitive decline	3AE4.00	70057
GDS level 6 - severe cognitive decline	3AE5.00	94717
GDS level 7 - very severe cognitive decline	3AE6.00	72520
[X]Mild cognitive disorder	Eu05700	11936
[X]Oth & unspec symptom/sign involv cognit funct/awareness	Ryu5100	52939
Health of the Nation Outcome Scale item 4 - cognitive probl	ZRLfE00	35194
Impaired cognition	Z7C1.00	10822
Cognitive function observations	Z7C..00	18906

Cognitive Screening Tests

Read term	Read code	Medcode
Cognitive assessment	311B.00	95853
GPCOG - general practitioner assessment of cognition	38Dv.00	101074
GPCOG (GP assessment of cognition) patient examination	38Dv000	103076
GPCOG (GP assessment of cognition) informant interview	38Dv100	101710
Six item cognitive impairment test	3AD3.00	35305
Cognitions questionnaire	ZR3a.00	36650
Cognitive failures questionnaire	ZR3b.00	66080
Microcog - assessment of cognitive function	ZRa2.00	22257
Neurobehavioural cognitive status examination	ZRaY.00	42059
Rancho scale - levels of cognitive functioning	ZRd..00	92756
Recognition memory test	ZRh6.00	96881

For peer review only

RMT - Recognition memory test	ZRh6.11	93236
Smith cognitive questionnaire	ZRkL.00	96828
Health of the Nation Outcome Scale item 4 - cognitive probl	ZRLfE00	35194
Kendrick cognitive tests for the elderly	ZRV9.11	63604
Lowenstein OT cognitive assessment	ZRVa.00	26264
LOTCA - Lowenstein OT cognitive assessment	ZRVa.11	98256
Measurement of cognitive linguistic ability	ZRVt.00	62114
MCLA - Measurement of cognitive linguistic ability	ZRVt.11	93194

Dementia Annual Review

**Read term**	**Read code**	**Medcode**
Dementia annual review	6AB..00	12710

Memory Loss Codes

**Read term**	**Read code**	**Medcode**
Memory loss - amnesia	1B1A.00	1993
Poor auditory sequential memory	1B1a.00	40821
Amnesia symptom	1B1A.11	3639
Memory loss symptom	1B1A.12	5777
Memory disturbance	1B1A.13	2908
Temporary loss of memory	1B1A000	27788
Short-term memory loss	1B1A100	103453
Transient global amnesia	1B1S.00	28278
Poor visual sequential memory	1B1Y.00	39507
Disturbance of memory for order of events	1S21.00	67163
Forgetful	28G..00	7742
Memory: own age not known	3A10.00	52947
Memory: present time not known	3A20.00	53146
Memory: present place not knwn	3A30.00	53014
Memory: present year not known	3A40.00	52948
Memory: own DOB not known	3A50.00	53125
Memory: present month not knwn	3A60.00	52825
Memory: important event not kn	3A70.00	52800

For peer review only

Memory: import.person not knwn	3A80.00	52801
Memory: count down unsuccess.	3A91.00	52805
Memory: address recall unsucc.	3AA1.00	53016
Patient forgets to take medication	8BIk.00	68703
Non-alcoholic amnestic syndrome	E040.00	27414
Hysterical amnesia	E201700	4269
Mild memory disturbance	E2A1000	6387
Organic memory impairment	E2A1100	6061
[X]Organic amnesic synd not induced alc/oth psychoact subs	Eu03.00	37072
[X]Dissociative amnesia	Eu44000	40994
Transient global amnesia	G655.00	6489
[D]Amnesia (retrograde)	R00z000	4284
[D]Memory deficit	R00z011	7711
[D]Anterograde amnesia	R00z500	54882
[X]Other amnesia	Ryu5000	70677
Memory disturbance (& amnesia (& symptom))	Z7CE400	51379
Memory loss symptom	Z7CE412	67838
Memory loss - amnesia	Z7CE413	103375
Memory disturbance	Z7CE414	105538
Loss of memory	Z7CE415	102880
Forgetful	Z7CE500	24952
Amnesia	Z7CE600	28406
Memory loss	Z7CE611	10123
Memory gone	Z7CE612	68230
Dysmnesia	Z7CE613	66001
Memory loss - amnesia	Z7CE614	12805
Loss of memory	Z7CE615	19297
LOM - Loss of memory	Z7CE616	12277
Transient global amnesia	Z7CE700	18996
TGA - Transient global amnesia	Z7CE711	19004
Anterograde amnesia	Z7CE800	36767
Antegrade amnesia	Z7CE811	43905
Retrograde amnesia	Z7CE900	35538
RA - Retrograde amnesia	Z7CE911	70703
Impairment of registration	Z7CEA00	49897
Impairment of working memory	Z7CEA11	32367
Impairment of immediate recall	Z7CEA12	59844
Impairment of primary memory	Z7CEA13	65696
Amnesia for remote events	Z7CEB00	66144

For peer review only

Poor memory for remote events	Z7CEB12	37191
Amnesia for recent events	Z7CEC00	61816
Loss of memory for recent events	Z7CEC11	9786
No memory for recent events	Z7CEC12	67802
Amnesia for day to day facts	Z7CED00	68392
Amnesia for important personal information	Z7CEE00	57812
Temporary loss of memory	Z7CEF00	67998
Transient memory loss	Z7CEG00	47882
Memory impairment	Z7CEH00	10514
Memory dysfunction	Z7CEH11	39915
Memory deficit	Z7CEH12	50418
Bad memory	Z7CEH13	26434
Memory problem	Z7CEH14	12057
Poor memory	Z7CEH15	12583
Mixes past with present	Z7CEI00	103922
Memory lapses	Z7CEJ00	19073
Minor memory lapses	Z7CEK00	53507
Mild memory disturbance	Z7CEL00	51724
Distortion of memory	Z7CEM00	51739
Invents experiences to compensate for loss of memory	Z7CEN11	64892
Poor short-term memory	Z7CF800	11410
Short-term memory loss	Z7CF811	10571
Unable to recall random address at five minutes	Z7CFA00	106055
Unable to recall five digit number at five minutes	Z7CFC00	104514
Unable to remember name of current prime minister	Z7CFe00	93856
Forgets what was going to do	Z7CFF00	59515
Forgets what was going to say	Z7CFG00	60600
Cannot remember names of intimates	Z7CFg00	105793
Forgets recent activities	Z7CFH00	41366
Cannot remember birth dates of children	Z7CFh00	54430
Forgets what has just done	Z7CFI00	40091
Cannot remember wedding anniversary	Z7CFi00	62749
Forgets what has just said	Z7CFJ00	93319
Forgets what has just read	Z7CFK00	70895
Forgets what has just seen	Z7CFL00	103479
Forgets what has just heard	Z7CFM00	47994
Poor long-term memory	Z7CFO00	53978
Long-term memory loss	Z7CFO11	47581

For peer review only

Unable to remember motor skills	Z7CFq00	46314
Unable to remember own date of birth	Z7CFQ00	63942
Unable to remember own age	Z7CFS00	32599
Unable to remember new motor skills	Z7CFs00	57750
Cannot remember own age	Z7CFS11	69118
Unable to remember day of the week	Z7CFU00	63849
Memory aided by use of diary	Z7CFw00	59830
Unable to remember today's date	Z7CFW00	66684
Memory aided by use of labels	Z7CFx00	98287
Memory aided by use of lists	Z7CFz00	67951
Delayed verbal memory	Z7CGP00	98798

MMSE

**Read term**	**Read code**	**Medcode**
Mini-mental state examination	388m.00	82481
MMSE score	388m.11	83484
Mini mental state score	388V.00	11862
Mini-mental state examination	ZRaA.00	10503
MMSE - Mini-mental state examination	ZRaA.11	10493
Modified mini-mental state 3MS examination	ZRaA100	59387
Modified mini-mental state examination	ZRaA200	36637

Referral to memory assessment services

**Read term**	**Read code**	**Medcode**
Referral to memory clinic	8HTY.00	82481
Seen in memory clinic	9Nk1.00	83484

Referral to Psychiatrist, Neurologist or Geriatrician

**Read term**	**Read code**	**Medcode**
Neurological referral	8H46.00	2964
Geriatric referral	8H47.00	1511
Referral to community geriatrician	8H47000	105235
Psychiatric referral	8H49.00	2189
Referral to psychogeriatrician	8H4D.00	5338
Referral to neurologist	8H4h.00	95859

For peer review only

Referral to neurology special interest general practitioner	8H4Y.00	55362
Psychiatric self-referral	8HJ3.00	27640
Geriatric self-referral	8HJ4.00	68924
Neurology self-referral	8HJE.00	40890
Urgent referral to psychiatrist	8HIB.00	95936
Private referral to neurologist	8HVL.00	13653
Private referral to geriatrician	8HVM.00	29200
Private referral to psychiatrist	8HVO.00	13677
Private referral to psychogeriatrician	8HVS.00	34532
Referral to care of the elderly physician	ZL5A200	12077
Referral to geriatrician	ZL5A211	11634
Referral to neurologist	ZL5AO00	10448
Referral to psychiatrist	ZL5B.00	10002

Alzheimer's prescription Product Codes (specific to CPRD)

Code	Product Description	Molecule	Dose, format and route
7329	Galantamine 20mg/5ml oral solution sugar free	Galantamine hydrobromide	4mg/1ml, Oral solution, Oral
36976	Rivastigmine 4.6mg/24hours transdermal patches	Rivastigmine	4.6mg/24 Hours, Transdermal Patch, Transdermal
33007	RIVASTIGMINE	Unknown	Unknown
56771	Rivastigmine 3mg capsules (Dr Reddy's Laboratories (UK) Ltd)	Rivastigmine hydrogen tartrate	3mg, Capsule, Oral
11751	Rivastigmine 3mg capsules	Rivastigmine hydrogen tartrate	3mg, Capsule, Oral
53922	Donepezil 10mg orodispersible tablets (Consilient Health Ltd)	Donepezil hydrochloride	10mg, Orodispersible tablet, Oral
10255	Galantamine 8mg modified-release capsules	Galantamine Hydrobromide	8mg, Modified Release Capsules, Oral
38976	Memantine 5mg+10mg+15mg+20mg Tablet	Memantine Hydrochloride	5mg+10mg+15mg+20mg, Tablet, Oral
33008	RIVASTIGMINE	Unknown	Unknown
33009	RIVASTIGMINE	Unknown	Unknown
60723	Rivastigmine 6mg capsules (Waymade Healthcare Plc)	Rivastigmine hydrogen tartrate	6mg, Capsule, Oral
56600	Donepezil 5mg tablets (Zentiva)	Donepezil hydrochloride	5mg, Tablet, Oral
11752	Rivastigmine 4.5mg capsules	Rivastigmine hydrogen tartrate	4.5mg, Capsule, Oral
11635	Galantamine 12mg tablets	Galantamine hydrobromide	12mg, Tablet, Oral
48443	Donepezil 10mg orodispersible tablets	Donepezil hydrochloride	10mg, Orodispersible tablet, Oral

For peer review only

53882	Rivastigmine 2mg/ml oral solution	Rivastigmine hydrogen tartrate	2mg/1ml, Oral solution, Oral
2931	Donepezil 10mg tablets	Donepezil hydrochloride	10mg, Tablet, Oral
6225	Memantine 10mg tablets	Memantine hydrochloride	10mg, Tablet, Oral
63217	Donepezil 5mg tablets (A A H Pharmaceuticals Ltd)	Donepezil hydrochloride	5mg, Tablet, Oral
11654	Galantamine 8mg tablets	Galantamine hydrobromide	8mg, Tablet, Oral
56631	Rivastigmine 13.3mg/24hours transdermal patches	Rivastigmine	13.3mg/24hour, Transdermal patch, Transdermal
39240	Memantine 20mg tablets	Memantine hydrochloride	20mg, Tablet, Oral
63951	Rivastigmine 9.5mg/24hours transdermal patches (Actavis UK Ltd)	Rivastigmine	9.5mg/24hour, Transdermal patch, Transdermal
11827	Rivastigmine 2mg/ml oral solution sugar free	Rivastigmine hydrogen tartrate	2mg/1ml, Oral solution, Oral
35088	Donepezil 10mg orodispersible tablets sugar free	Donepezil Hydrochloride	10mg, Orodispersible Tablet, Oral
60107	Donepezil 5mg tablets (Alliance Healthcare (Distribution) Ltd)	Donepezil Hydrochloride	5mg, tablet, Oral
10187	Galantamine 4mg tablets	Galantamine hydrobromide	4mg, Tablet, Oral
58969	Rivastigmine 4.6mg/24hours transdermal patches (A A H Pharmaceuticals Ltd)	Rivastigmine	4.6mg/24hour, Transdermal patch, Transdermal
59871	Donepezil 10mg/5ml oral suspension	Donepezil hydrochloride	2mg/1ml, Oral suspension, Oral
2930	Donepezil 5mg tablets	Donepezil hydrochloride	5mg, Tablet, Oral
35179	Donepezil 5mg orodispersible tablets sugar free	Donepezil hydrochloride	5mg, Orodispersible tablet, Oral
7361	Galantamine 24mg modified-release capsules	Galantamine hydrobromide	24mg, Modified-release capsule, Oral
4597	Rivastigmine 1.5mg capsules	Rivastigmine hydrogen tartrate	1.5mg, Capsule, Oral
61676	Donepezil 1mg/ml oral solution sugar free	Donepezil hydrochloride	1mg/1ml, Oral solution, Oral
58947	Donepezil 10mg tablets (Accord Healthcare Ltd)	Donepezil hydrochloride	10mg, Tablet, Oral
14309	Galantamine 16mg modified-release capsules	Galantamine Hydrobromide	16mg, Modified Release Capsules, Oral
11837	Memantine 10mg/ml oral solution sugar free	Memantine hydrochloride	10mg/1ml, Oral drops, Oral
58709	Donepezil 10mg tablets (A A H Pharmaceuticals Ltd)	Donepezil hydrochloride	10mg, Tablet, Oral

For peer review only

9786	Rivastigmine 6mg capsules	Rivastigmine hydrogen tartrate	6mg, Capsule, Oral
37132	Rivastigmine 9.5mg/24hours transdermal patches	Rivastigmine	9.5mg/24hour, Transdermal patch, Transdermal
48442	Donepezil 5mg orodispersible tablets	Donepezil hydrochloride	5mg, Orodispersible tablet, Oral -

For peer review only

Keyword	Stem	misspellings	
**Memory**	memory		
	memori*	memroy	
		memeory	
		memmory	
		meory	
		memort	
		memrory	
		memorry	
**cognition**	cognition	cogition	
	cognitive	cogntion	
	cognit*	cngition	
		congtion	
		cogniton	
		congit*	
		cgnit*	
**forgetfulness**	forgetting	foget*	
	forgetful*	forgot*	
	forget*	forgte	
		fforget*	
		froget*	
**confusion**	confusion	confudes	
	confused	condusef	
	confus*	cofnused	
**dementia**	dementia		
	dement*		
	Alzheimer's	alzheimers	
		alzhemer	
		alzhiemer	
		alzhiemers	
		alzhimer	
		alzhimers	
		alzimer	
		alz*	
**behaviour change**	behaviour	behavour	
	behavior	bahaviour	
	behav*	bahavior	
		bahavioe	
		behavoir	
		behaveour	
**family concerned**	family + concerned	**for family substitute:**	**for concerned substitute**
		relative	conserned
		wife	concern*
		husband	worried
		son	
		daughter	
		partner	
		spouse	
		niece	

For peer review only

		nephew
		sister
		brother
		relative
		neighbour
**3rd Party**	third party consultation	third party consulation
		3rd party consultation
		3rd party consulation
**MMSE**	MMSE	
	mini mental state	
	mini-mental state	
	GP Cog	

For peer review only

Appendix 4: Sensitivity analysis.

We removed controls with Dementia annual review codes (N = 146), and Alzheimer's medical prescription codes (154 additional patients).

We re-ran the models as before with the predictors of Alzheimer's medical prescription and dementia annual review removed. We show below that the model performance (based on AUC) dropped in the codes-only models, but was largely maintained in the codes and keywords models.

Table 4.1: Model performance with Alzheimer's medical prescription and Dementia annual review removed from control group and model

	Codes only				Codes and Keywords			
Classifier	AUC	Sensitivity	Specificity	PPV at 7.1% prevalence	AUC	Sensitivity	Specificity	PPV at 7.1% prevalence
Random Forest	0.83	0.69	0.95	0.61	0.93	0.85	0.93	0.49
Logistic Regression	0.83	0.69	0.95	0.61	0.93	0.84	0.92	0.48
Naive Bayes	0.83	0.69	0.95	0.60	0.89	0.79	0.90	0.41

Table 4.2: Feature weights with Alzheimer's medical prescription and Dementia annual review removed from control group and model

	Coded data only			Codes and Keywords		
	Logistic Regression	Random Forest	Naive Bayes	Logistic Regression	Random Forest	Naive Bayes
Cognitive Decline	0.320	0.091	0.529	0.244	0.071	0.529
Cognitive Screening Test	0.008	0.007	0.571	0.083	0.007	0.571
Memory Loss Codes	1	0.799	1	1	0.888	1
MMSE	0.380	1	0.321	0.286	0.994	0.321
Referral To Memory Assessment	0.263	0.269	0.420	0.147	0.132	0.42
Referral To Psych/Geri/Neuro	0.246	0.0776	0.578	0.104	0.022	0.578
Keyword Dementia	-	-	-	0.311	0.923	0.258
Keyword Memory	-	-	-	0.171	1	0.232
Keyword Confusion	-	-	-	0.161	0.34	0.202
Keyword Behaviour	-	-	-	0.028	0.085	0.281
Keyword Cognition	-	-	-	0.025	0.071	0.279
Keyword Family	-	-	-	0.093	0.066	0.064
Keyword MMSE	-	-	-	0.025	0.108	0.280
Keyword Forget	-	-	-	0.033	0.089	0.242
Keyword Third party consultation	-	-	-	0.714	0.251	0.292

For peer review only

Figure 4.1: ROC curves for model with Alzheimer's medical prescription and Dementia annual review removed, codes only.

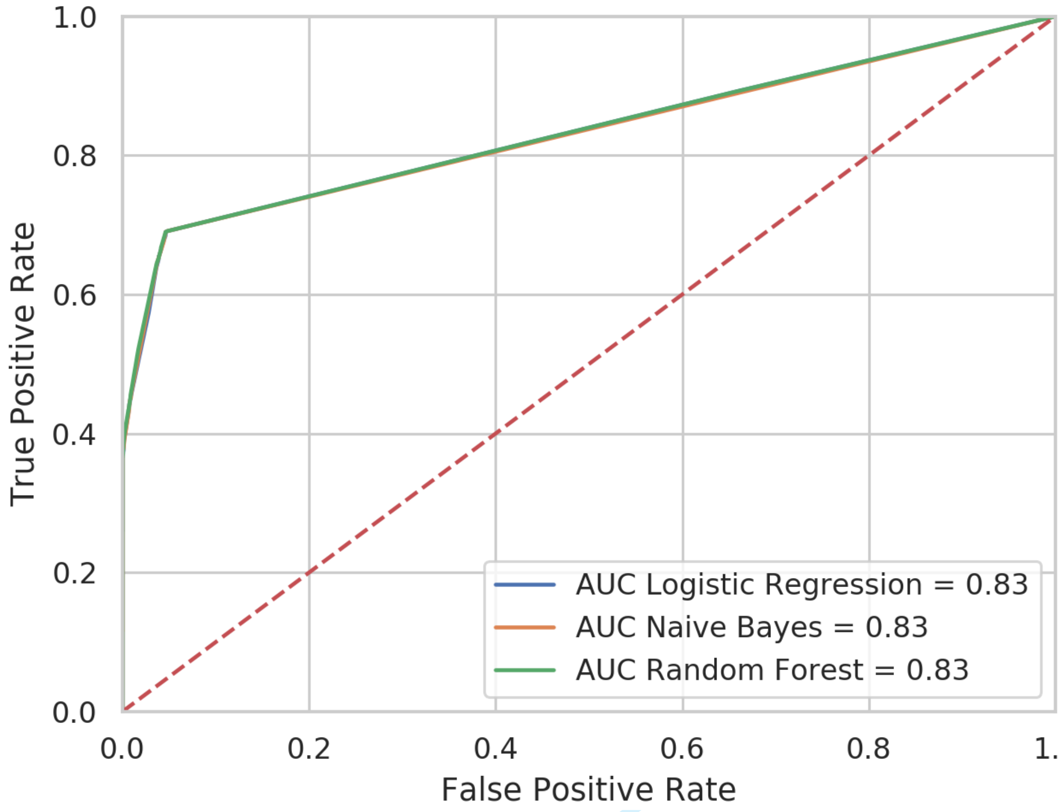

Figure 4.2: ROC curves for model with Alzheimer's medical prescription and Dementia annual review removed, codes and keywords.

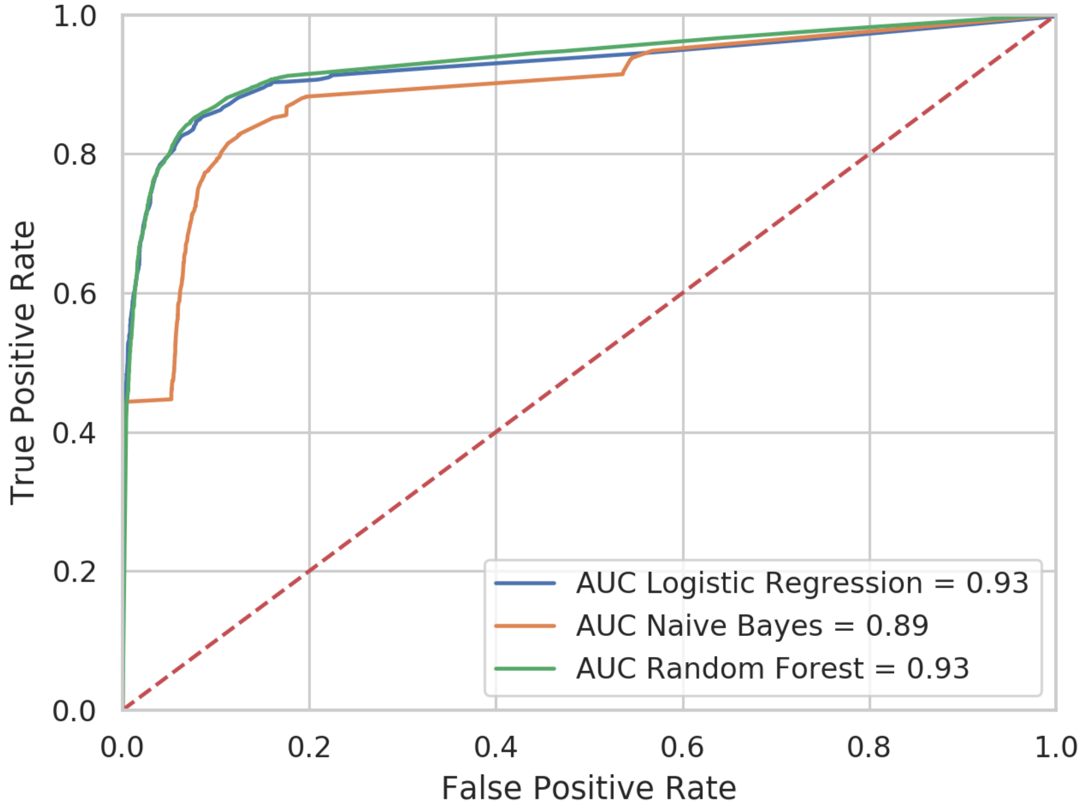

The RECORD statement – checklist of items, extended from the STROBE statement, that should be reported in observational studies using routinely collected health data.

	Item No.	STROBE items	Location in manuscript where items are reported	RECORD items	Location in manuscript where items are reported
**Title and abstract**					
	1	(a) Indicate the study's design with a commonly used term in the title or the abstract (b) Provide in the abstract an informative and balanced summary of what was done and what was found		RECORD 1.1: The type of data used should be specified in the title or abstract. When possible, the name of the databases used should be included.<br><br>RECORD 1.2: If applicable, the geographic region and timeframe within which the study took place should be reported in the title or abstract.<br><br>RECORD 1.3: If linkage between databases was conducted for the study, this should be clearly stated in the title or abstract.	Title and abstract<br><br>Abstract<br><br>N/A
**Introduction**					
Background rationale	2	Explain the scientific background and rationale for the investigation being reported			4-6
Objectives	3	State specific objectives, including any prespecified hypotheses			6
**Methods**					
Study Design	4	Present key elements of study design early in the paper			6
Setting	5	Describe the setting, locations, and relevant dates, including periods of recruitment, exposure, follow-up, and data collection			6-7

For peer review only

Participants	6	*(a) Cohort study* - Give the eligibility criteria, and the sources and methods of selection of participants. Describe methods of follow-up<br>*Case-control study* - Give the eligibility criteria, and the sources and methods of case ascertainment and control selection. Give the rationale for the choice of cases and controls<br>*Cross-sectional study* - Give the eligibility criteria, and the sources and methods of selection of participants<br><br>*(b) Cohort study* - For matched studies, give matching criteria and number of exposed and unexposed<br>*Case-control study* - For matched studies, give matching criteria and the number of controls per case	7-8<br><br>7-8	RECORD 6.1: The methods of study population selection (such as codes or algorithms used to identify subjects) should be listed in detail. If this is not possible, an explanation should be provided.<br><br>RECORD 6.2: Any validation studies of the codes or algorithms used to select the population should be referenced. If validation was conducted for this study and not published elsewhere, detailed methods and results should be provided.<br><br>RECORD 6.3: If the study involved linkage of databases, consider use of a flow diagram or other graphical display to demonstrate the data linkage process, including the number of individuals with linked data at each stage.	7 (Appendix 1)<br><br>N/A<br><br>N/A
Variables	7	Clearly define all outcomes, exposures, predictors, potential confounders, and effect modifiers. Give diagnostic criteria, if applicable.	8-9	RECORD 7.1: A complete list of codes and algorithms used to classify exposures, outcomes, confounders, and effect modifiers should be provided. If these cannot be reported, an explanation should be provided.	8-9 and Supplementary Materials (Appendix 2 & 3)
Data sources/ measurement	8	For each variable of interest, give sources of data and details of methods of assessment (measurement).<br>Describe comparability of assessment methods if there is more than one group	8-9		

For peer review only

Bias	9	Describe any efforts to address potential sources of bias	7		
Study size	10	Explain how the study size was arrived at	7		
Quantitative variables	11	Explain how quantitative variables were handled in the analyses. If applicable, describe which groupings were chosen, and why	8-9		
Statistical methods	12	(a) Describe all statistical methods, including those used to control for confounding<br>(b) Describe any methods used to examine subgroups and interactions<br>(c) Explain how missing data were addressed<br>(d) *Cohort study* - If applicable, explain how loss to follow-up was addressed<br>*Case-control study* - If applicable, explain how matching of cases and controls was addressed<br>*Cross-sectional study* - If applicable, describe analytical methods taking account of sampling strategy<br>(e) Describe any sensitivity analyses	9-10<br>N/A<br>Missing data were not addressed<br>N/A<br>7<br>N/A<br>No sensitivity analyses		
Data access and cleaning methods		..		RECORD 12.1: Authors should describe the extent to which the investigators had access to the database population used to create the study population.	7-8

For peer review only

				RECORD 12.2: Authors should provide information on the data cleaning methods used in the study.	8 and Figure 1
Linkage		..		RECORD 12.3: State whether the study included person-level, institutional-level, or other data linkage across two or more databases. The methods of linkage and methods of linkage quality evaluation should be provided.	7: No data linkage.
**Results**					
Participants	13	(a) Report the numbers of individuals at each stage of the study (*e.g.*, numbers potentially eligible, examined for eligibility, confirmed eligible, included in the study, completing follow-up, and analysed)<br>(b) Give reasons for non-participation at each stage.<br>(c) Consider use of a flow diagram	Figure 1 and page 7	RECORD 13.1: Describe in detail the selection of the persons included in the study (*i.e.,* study population selection) including filtering based on data quality, data availability and linkage. The selection of included persons can be described in the text and/or by means of the study flow diagram.	Figure 1 and page 7
Descriptive data	14	(a) Give characteristics of study participants (*e.g.*, demographic, clinical, social) and information on exposures and potential confounders<br>(b) Indicate the number of participants with missing data for each variable of interest<br>(c) *Cohort study* - summarise follow-up time (*e.g.*, average and total amount)	11<br><br>Missingness not assessed because of confounding of missing with negative values.		
Outcome data	15	*Cohort study* - Report numbers of outcome events or summary measures over time<br>*Case-control study* - Report numbers in each exposure	11 and Table 1		

For peer review only - http://bmjopen.bmj.com/site/about/guidelines.xhtml

		category, or summary measures of exposure<br>*Cross-sectional study* - Report numbers of outcome events or summary measures			
Main results	16	(a) Give unadjusted estimates and, if applicable, confounder-adjusted estimates and their precision (e.g., 95% confidence interval). Make clear which confounders were adjusted for and why they were included<br>(b) Report category boundaries when continuous variables were categorized<br>(c) If relevant, consider translating estimates of relative risk into absolute risk for a meaningful time period	a) Relative estimates of feature weighting in the classification given in Table 3.<br>b) N/A<br>c) N/A		
Other analyses	17	Report other analyses done—e.g., analyses of subgroups and interactions, and sensitivity analyses	N/A		
**Discussion**					
Key results	18	Summarise key results with reference to study objectives	13-14		
Limitations	19	Discuss limitations of the study, taking into account sources of potential bias or imprecision. Discuss both direction and magnitude of any potential bias	16-17	RECORD 19.1: Discuss the implications of using data that were not created or collected to answer the specific research question(s). Include discussion of misclassification bias, unmeasured confounding, missing data, and changing eligibility over time, as they pertain to the study being reported.	16-17
Interpretation	20	Give a cautious overall interpretation of results considering objectives,	17		

For peer review only

		limitations, multiplicity of analyses, results from similar studies, and other relevant evidence			
Generalisability	21	Discuss the generalisability (external validity) of the study results	16		
**Other Information**					
Funding	22	Give the source of funding and the role of the funders for the present study and, if applicable, for the original study on which the present article is based	18		
Accessibility of protocol, raw data, and programming code		..		RECORD 22.1: Authors should provide information on how to access any supplemental information such as the study protocol, raw data, or programming code.	Protocol will be supplied on application to authors.

*Reference: Benchimol EI, Smeeth L, Guttmann A, Harron K, Moher D, Petersen I, Sørensen HT, von Elm E, Langan SM, the RECORD Working Committee. The REporting of studies Conducted using Observational Routinely-collected health Data (RECORD) Statement. *PLoS Medicine* 2015; in press.

*Checklist is protected under Creative Commons Attribution (CC BY) license.
